# Resorcylic Acid Lactones and Isocoumarin Derivatives from the Marine-Associated Polar Fungus *Penicillium* sp. OUCMDZ-4014

**DOI:** 10.3390/md24080256

**Published:** 2026-07-24

**Authors:** Deng Yu, Rongtian Du, Xuehan Yu, Chengze An, Chenyang Ding, Jiapeng Wang, Weiming Zhu, Yi Wang

**Affiliations:** 1 School of Medicine and Pharmacy, Ocean University of China, Qingdao 266003, China; yudeng233@126.com (D.Y.); hoshikawaren@163.com (R.D.); 19556165540@163.com (X.Y.); 13342482972@163.com (C.A.); dcy@ouc.edu.cn (C.D.); 18764752210@163.com (J.W.); 2 Laboratory for Marine Drugs and Bioproducts, Qingdao Marine Science and Technology Centre, Qingdao 266237, China; 3 Key Laboratory of Marine Drugs, Ministry of Education of China, Ocean University of China, Qingdao 266003, China

**Keywords:** marine-associated fungus, *Penicillium* sp., resorcylic acid lactones, isocoumarins, α-glucosidase inhibition

## Abstract

Marine-associated polar fungi are promising sources of structurally diverse natural products. To investigate polyketide metabolites from the Antarctic moss-derived fungus *Penicillium* sp. OUCMDZ-4014, HSQC NMR combined with DeepSAT analysis was used to prioritize fractions enriched in cyclic and aromatic polyketides, and chromane-like metabolites. Chromatographic separation yielded ten resorcylic acid lactone-derived aromatic polyketides, including RAL macrolides, ring-opened esters, and isocoumarins, among which compounds **4**–**7** were new. Their planar structures were established by HRESIMS and 1D/2D NMR analyses. Their chemical structures including configurations were established by HRESIMS, 1D/2D NMR, ECD calculations, 13C NMR calculations, and DP4+ analysis. Isocoumarins **7**–**10** displayed diverse levels of α-glucosidase inhibition, with compound **9** being the most active (IC_50_ = 47.0 ± 3.83 μM). Kinetic analysis indicated noncompetitive inhibition by **9** and mixed-type inhibition by **10**. Genome mining revealed a putative *res* biosynthetic gene cluster containing HR-PKS, NR-PKS, and tailoring-enzyme genes. Compound **1** underwent nonenzymatic conversion into **9** under culture-medium conditions, while trace conversion was also observed during concentration, revealing a chemical link between the RAL macrolide and isocoumarin scaffolds, and highlighting the contribution of post-biosynthetic chemical transformation to fungal polyketide diversification.

## 1. Introduction

Natural products have long served as important sources for drug discovery and lead compound development, and marine natural products occupy a particularly important position because of their unique ecological origins and remarkable structural diversity [1,2]. Marine microorganisms, especially marine-derived or marine-associated fungi, are capable of producing a wide range of structurally novel and biologically diverse secondary metabolites, making them promising resources for marine drug discovery [3,4]. Compared with ordinary terrestrial environments, polar and marine-influenced habitats are characterized by low temperature, strong ultraviolet radiation, oligotrophic conditions, and pronounced environmental fluctuations. These ecological pressures may drive microorganisms to develop unique metabolic adaptation mechanisms and produce natural products with novel scaffolds and potential bioactivities [5]. Therefore, systematic investigation of secondary metabolites from marine-associated polar fungi is not only important for expanding the chemical space of marine natural products but also provides valuable opportunities for discovering new bioactive molecules with pharmaceutical potential.

Fungal polyketides represent one of the most important classes of secondary metabolites from marine fungi. Among them, resorcylic acid lactones (RALs) and related derivatives have attracted considerable attention because of their characteristic macrolactone scaffolds and diverse biological activities [6]. A variety of RALs and resorcylic acid derivatives have been reported from marine-derived or marine-associated fungi, such as zearalanone-type RAL derivatives from marine-derived *Penicillium* sp., cochliomycin-type compounds from the sea anemone-derived fungus *Cochliobolus lunatus*, and resorcylic acid derivatives and related ring-opened congeners from the mangrove-derived fungus *Penicillium brocae* [7,8,9]. From a biosynthetic perspective, fungal RAL scaffolds are generally assembled through the collaborative action of a highly reducing polyketide synthase (HR-PKS) and a nonreducing polyketide synthase (NR-PKS), in which the HR-PKS is responsible for generating the reduced aliphatic polyketide chain, whereas the NR-PKS participates in the construction of the aromatic polyketide core and subsequent cyclization processes [10,11]. This HR-PKS/NR-PKS cooperative assembly mode provides a biosynthetic basis for chain-length variation, redox diversity, and scaffold diversification in RAL-type metabolites.

Isocoumarins are another important class of aromatic polyketide derivatives and are widely distributed in fungi, plants, and marine microorganisms. These compounds exhibit a broad range of biological activities, including antibacterial, anti-inflammatory, antioxidant, and enzyme inhibitory effects [12]. In recent years, increasing evidence has shown that fungal isocoumarins, coumarins, and other aromatic polyketides can act as α-glucosidase inhibitors, providing important chemical sources for the discovery of antidiabetic lead compounds [13]. For example, isocoumarin and α-pyranone derivatives recently isolated from a mangrove-associated fungus were also reported to possess α-glucosidase inhibitory activity [14]. Therefore, investigation of the structural and biosynthetic relationships among RAL macrolides and isocoumarin derivatives may not only clarify the origin of structural diversity in fungal aromatic polyketides but also provide a chemical basis for the discovery of new bioactive lead compounds.

In complex natural product extracts or fractions, rapid recognition of target compound classes with potential novelty is a key issue for improving discovery efficiency. In recent years, NMR-based dereplication and structural classification strategies have been widely applied in natural product research. Minimal NMR datasets, including ^1^H NMR, ^1^H–^13^C HSQC, and COSY data, can assist in dereplication and structural fragment recognition of natural products [15]. In addition, two-dimensional NMR-based compound networking has been used for rapid identification of fungal metabolites and has been successfully applied to the dereplication of RALs and other fungal natural products [16]. DeepSAT is a deep learning tool based on ^1^H–^13^C HSQC spectral information, which can predict molecular fingerprints, molecular weights, and chemical classes according to HSQC chemical shift distributions, thereby accelerating small-molecule structural annotation and analogue discovery [17]. Therefore, the combination of HSQC spectral analysis and DeepSAT-assisted classification can serve as an efficient strategy for the rapid recognition and prioritization of structurally related polyketide metabolites in complex fractions.

In addition, the development of genome mining has provided an important tool for elucidating the biosynthetic origins of natural products. Biosynthetic genes responsible for fungal secondary metabolites are generally organized in gene clusters, and many fungal genomes still contain substantial unexplored biosynthetic potential [18]. By integrating chemical structural information, PKS domain organization, and biosynthetic gene cluster (BGC) functional annotation, reasonable hypotheses can be proposed for the biosynthetic origins and structural diversification mechanisms of natural products. In particular, for structural systems such as RAL macrolides and isocoumarin derivatives that may share a common polyketide origin, the combination of genome mining and chemical transformation experiments is useful for understanding the relationship between enzyme-catalyzed biosynthesis and post-biosynthetic nonenzymatic transformation.

As part of our continuing investigation of secondary metabolites from marine-associated polar fungi, *Penicillium* sp. OUCMDZ-4014, isolated from Antarctic moss, was investigated for its aromatic polyketide metabolites. In this study, HSQC NMR combined with DeepSAT analysis was used to evaluate the chemical features of the crude fractions and revealed an enrichment of cyclic polyketides, aromatic polyketides, and chromane-like metabolites. Subsequent chromatographic separation and structural elucidation yielded ten resorcylic acid lactone-derived aromatic polyketides, including RAL macrolides, ring-opened esters, and isocoumarins, among which compounds **4**–**7** were identified as new metabolites. Compounds **7**–**10** exhibited measurable α-glucosidase inhibition, with compound **9** being the most active one. Genome mining identified a putative *res* biosynthetic gene cluster containing HR-PKS, NR-PKS, and tailoring-enzyme genes, and a tentative HR-PKS/NR-PKS collaborative pathway was proposed for the formation and diversification of compounds **1**–**10**. Notably, chemical communication experiments demonstrated that the RAL macrolide **1** could nonenzymatically transform into the isocoumarin derivative 9. These findings expand the chemical diversity of RAL-derived polyketides from polar fungi and provide new insights into the combined roles of biosynthesis and post-biosynthetic chemical transformation in the diversification of fungal polyketide scaffolds.

## 2. Results and Discussion

### 2.1. Isolation and Structural Elucidation

**Figure 1:**
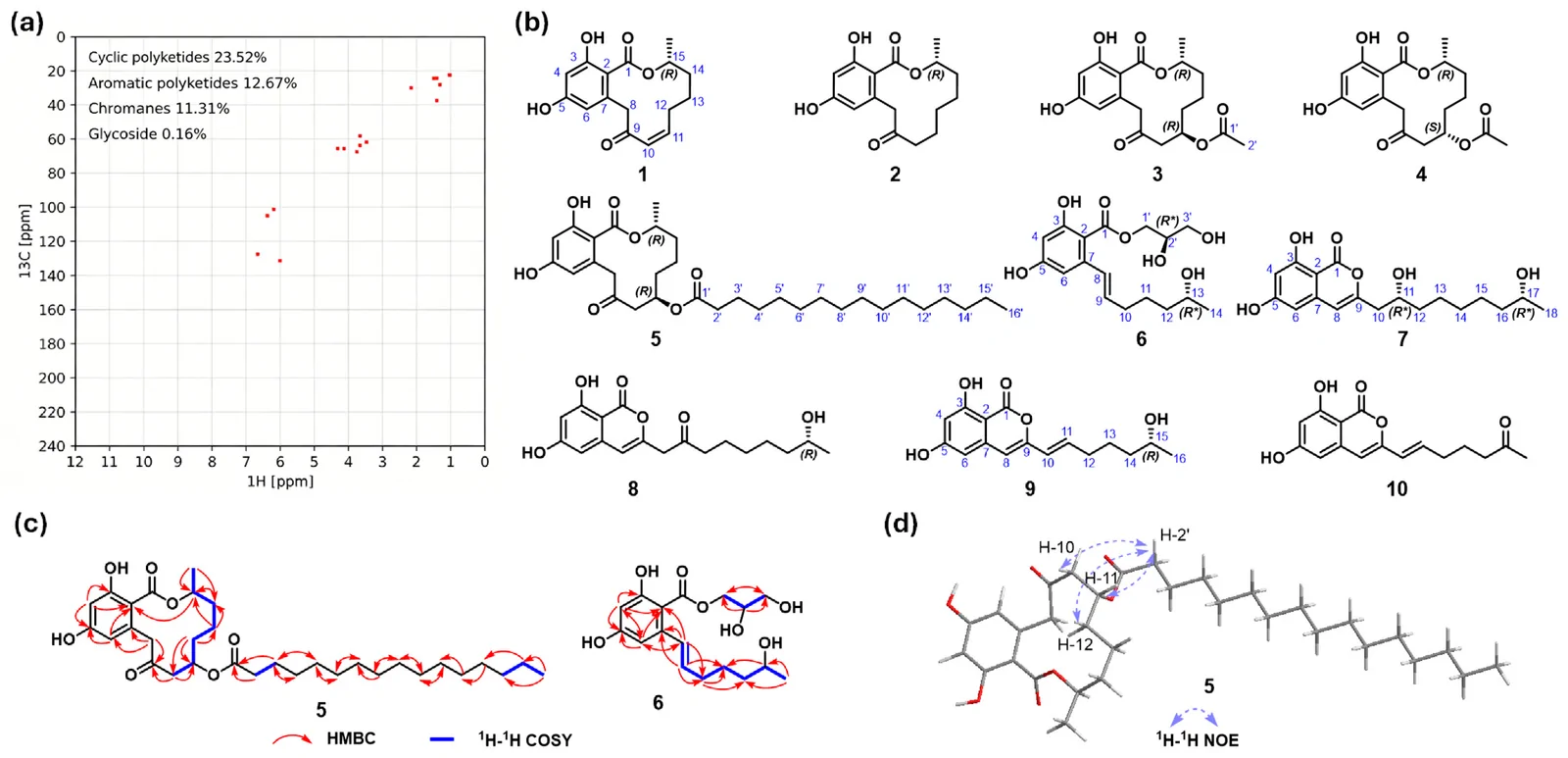
DeepSAT-assisted discovery and structural elucidation of resorcylic acid lactone-derived polyketides from *Penicillium* sp. OUCMDZ-4014. (**a**) DeepSAT-assisted HSQC analysis of the crude fractions, showing the predicted chemical class distribution of the major metabolites; (**b**) chemical structures of compounds **1**–**10** isolated from *Penicillium* sp. OUCMDZ-4014; (**c**) key HMBC and ^1^H–^1^H COSY correlations of compounds **5** and **6**; (**d**) key ^1^H–^1^H NOE correlations of compound **5**.

To rapidly evaluate the chemical features of the metabolites produced by *Penicillium* sp. OUCMDZ-4014, the crude fractions were first analyzed by HSQC NMR spectroscopy and further classified using the DeepSAT approach. The HSQC-based chemical shift distribution suggested that these fractions were enriched with cyclic polyketides, aromatic polyketides, and chromane-like metabolites (Figure 1a), indicating the presence of a series of structurally related polyketide congeners. Guided by this NMR-based dereplication and prioritization strategy, systematic chromatographic separation of the target fractions led to the isolation of ten resorcylic acid lactone-derived aromatic polyketides, including compounds **1**–**10** (Figure 1b). Among them, compounds **4**–**7** were identified as new metabolites, whereas compounds **1**–**3** and **8**–**10** were assigned as known analogues by comparison with reported spectroscopic data.

Compound **4** was obtained as a colorless oily solid. Its molecular formula was established as C_18_H_22_O_7_ based on the HRESIMS ion peak at *m*/*z* 351.1435 [M + H]^+^, corresponding to eight degrees of unsaturation. Analysis of the ^1^H and ^13^C NMR data revealed two aromatic proton signals at *δ*_H_ 6.21 and 6.10, indicating the presence of a substituted benzene ring. The ^13^C NMR spectrum showed six aromatic carbon resonances and three carbonyl carbon signals at *δ*_C_ 169.9, 169.9, and 204.9. Comprehensive comparison of the NMR data of **4** with those of penicimenolide B (**3**) revealed that they shared almost identical spectroscopic features and the same molecular formula (Figures S7–S9 and Table S4), suggesting that **4** was a stereoisomer of **3**. In the ECD spectrum, compound **4** exhibited a positive Cotton effect at 260 nm, by comparison with structurally related analogues, suggested an *R* configuration at C-15 [19,20,21,22,23]. Compound **4** showed an optical rotation of [α]^20^_D_ −24.4 (*c* 0.1, MeOH), opposite in sign to that of the co-metabolite **3**. Given their identical molecular formulas, highly similar NMR data, and plausible biogenetic relationship, this difference suggested that **4** may be the C-11 epimer of **3**. Accordingly, the absolute configuration of **4** was tentatively assigned as *11S*,*15R*.

Compound **5** was isolated as a pale-yellow oily solid. The molecular formula of **5** was determined to be C_32_H_50_O_7_ by HRESIMS, which showed protonated and sodiated molecular ion peaks at *m*/*z* 547.3621 [M + H]^+^ and 569.3439 [M + Na]^+^, respectively, corresponding to eight degrees of unsaturation. Analysis of the ^1^H and ^13^C NMR data revealed two aromatic proton signals at *δ*_H_ 6.37 and 6.10, six aromatic carbon resonances, and three carbonyl carbon signals at *δ*_C_ 171.1, 173.9, and 204.8, indicating a resorcylic acid lactone-type macrolide framework. The presence of a long aliphatic chain was suggested by the clustered methylene carbon resonances at *δ*_C_ 29.3–29.8. The HMBC correlations from H-2′ and H-3′ to C-1′ (*δ*_C_ 173.9) established the fatty acyl unit, while the correlation from H-11 to C-1′ (Figure 1c), together with the NOESY correlation between H-2′ and H-11 (Figure 1d), indicated that this fatty acyl chain was esterified at the C-11 hydroxy group (Figures S10–S17 and Table S5). Thus, the planar structure of **5** was assigned.

**Figure 2:**
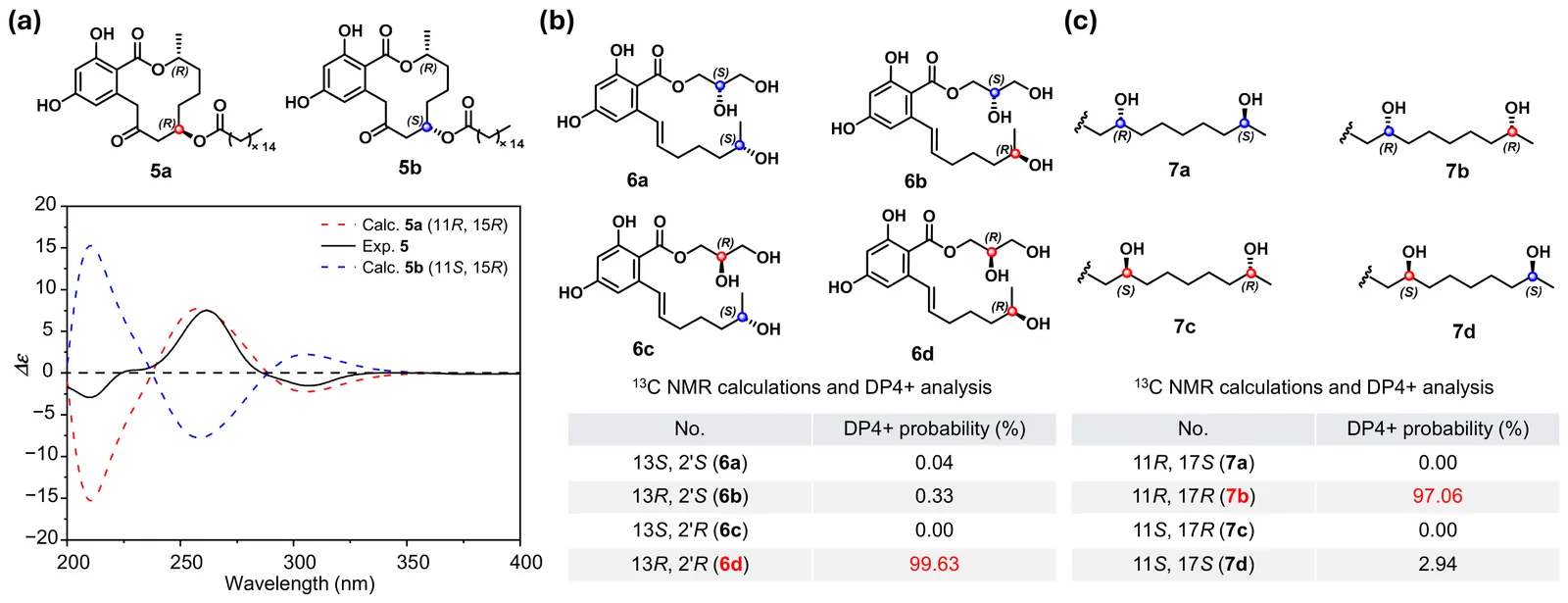
Stereochemical analyses of compounds **5**–**7**. (**a**) Experimental ECD spectrum of compound **5** and calculated ECD spectra of the candidate stereoisomers **5a** (11*R*,15*R*) and **5b** (11*S*,15*R*); (**b**) candidate stereoisomers of compound **6** and their DP4+ probabilities based on ^13^C NMR chemical shift calculations; (**c**) candidate stereoisomers of compound **7** and their DP4+ probabilities based on ^13^C NMR chemical shift calculations. The stereochemical relationships shown for compounds **6** and **7** represent tentative relative assignments.

The positive Cotton effect of **5** at 260 nm in the experimental ECD spectrum supported the *R* configuration at C-15. The absolute configuration of C-11 was further determined by comparison of the experimental ECD spectrum with the calculated ECD spectra of **5a** (11*R*,15*R*) and **5b** (11*S*,15*R*). The ECD calculations were performed at the B3LYP/6-31G(d) level. The calculated spectrum of **5a** matched well with the experimental curve, allowing the absolute configuration of **5** to be determined as 11*R*,15*R* (Figure 2a).

Compound **6** was obtained as a colorless oily solid. Its molecular formula was determined to be C_17_H_24_O_7_ by HRESIMS, which showed protonated and sodiated molecular ion peaks at *m*/*z* 341.1594 [M + H]^+^ and 363.1411 [M + Na]^+^, respectively, requiring six degrees of unsaturation. The ^1^H NMR spectrum displayed two aromatic proton signals at *δ*_H_ 6.37 and 6.17, two olefinic proton resonances at *δ*_H_ 6.60 and 5.99, and one methyl signal at *δ*_H_ 1.03. The ^13^C NMR spectrum exhibited six aromatic carbon signals, including two oxygenated aromatic carbons at *δ*_C_ 159.6 and 161.0, suggesting the presence of a highly oxygenated resorcinol-type benzene ring.

The three carbon resonances at *δ*_C_ 62.7, 69.2, and 65.7 indicated the presence of three oxygenated aliphatic carbons. The HMBC correlations from the oxymethylene protons at *δ*_H_ 4.12 and 4.23 to the carbonyl carbon at *δ*_C_ 169.0 suggested the presence of an ester group. The HMBC correlations from the two olefinic protons to the aromatic carbon at *δ*_C_ 139.9 indicated that the double bond was directly connected to the aromatic ring, forming a conjugated side chain (Figures S18–S22 and Table S6). In addition, the ^1^H–^1^H COSY correlations of H-1′/H-2′/H-3′ established a three-carbon oxygenated spin system connected to the ester moiety. The COSY correlations of H-9/H-10, H-10/H-11, H-11/H-12, and H-12/H-13 further defined a C-10–C-11–C-12–C-13 aliphatic fragment attached to the olefinic unit (Figure 1c). Based on these spectroscopic data, the planar structure of **6** was established.

Because compound **6** was obtained in limited quantity, its stereochemistry could not be independently determined by chemical derivatization. Therefore, ^13^C NMR chemical shift calculations at the mPW1PW91/6-311+G(d,p) level, combined with DP4+ probability analysis, were performed for four candidate stereoisomers: **6a** (*13S*,*2′S*), **6b** (*13R*,*2′S*), **6c** (*13S*,*2′R*), and **6d** (*13R*,*2′R*). However, because enantiomeric pairs cannot be distinguished by conventional NMR chemical shifts in an achiral medium, and because C-13 and C-2′ are remotely located on flexible acyclic fragments, the DP4+ results were interpreted cautiously and were not considered sufficient to establish the absolute configuration. The calculations provided tentative support for the *13R*,*2′R*/*13S*,*2′S* diastereomeric relationship over the *13R*,*2′S*/*13S*,*2′R* relationship. In addition, the proposed biogenetic relationship of **6** with structurally related co-metabolites provided supplementary support for an *R* configuration at the terminal stereogenic center C-13. On the basis of the combined DP4+ and biogenetic considerations (see Section 2.3), the *13R*,*2′R* stereoisomer was regarded as the most plausible candidate. Nevertheless, this assignment remains supportive and speculative rather than definitive. Accordingly, the relative configuration of **6** was tentatively assigned as 13R*,2′R*, whereas its absolute configuration remains undetermined (Figure 2b and Table S11).

Compound **7** was obtained as a yellow amorphous solid. Its molecular formula was determined to be C_18_H_24_O_6_ by HRESIMS, which showed protonated and sodiated molecular ion peaks at *m*/*z* 337.1653 [M + H]^+^ and 359.1470 [M + Na]^+^, respectively, requiring seven degrees of unsaturation. The ^1^H NMR spectrum displayed two aromatic proton signals at *δ*_H_ 6.26 and 6.21, and one olefinic proton resonance at *δ*_H_ 6.40. The ^13^C NMR spectrum exhibited six aromatic carbon signals and one carbonyl carbon resonance at *δ*_C_ 165.7, suggesting the presence of an isocoumarin-type aromatic polyketide skeleton.

Detailed comparison of the NMR data of **7** with those of **8**, which showed similar UV absorption features, revealed that the main difference between them occurred at C-11. The ketone carbonyl signal at *δ*_C_ 205.4 in **8** was replaced by an oxygenated methine carbon at *δ*_C_ 67.6 in **7**, indicating that C-11 in **7** was hydroxylated. Further comparison with the reported data of 3-(2,8-dihydroxynonyl)-6,8-dihydroxy-1H-isochromen-1-one showed that **7** possessed the same planar structure (Figures S23–S28 and Table S7) [24]. Because the two stereogenic centers at C-11 and C-17 are located at the two flexible termini of the aliphatic chain and are spatially remote from each other, their relative configuration could not be reliably established by NOESY correlations.

Because compound **7** was obtained in limited quantity, its stereochemistry could not be independently determined by chemical derivatization. Therefore, ^13^C NMR chemical shift calculations at the mPW1PW91/6-311+G(d,p) level, combined with DP4+ probability analysis, were performed for four candidate stereoisomers: **7a** (*11R*,*17S*), **7b** (*11R*,*17R*), **7c** (*11S*,*17R*), and **7d** (*11S*,*17S*). However, because enantiomeric pairs cannot be distinguished by conventional NMR chemical shifts in an achiral medium, and because C-11 and C-17 are remotely located on a flexible acyclic chain, the DP4+ results were interpreted cautiously and were not considered sufficient to establish the absolute configuration. The calculations provided tentative support for the *11R*,*17R*/*11S*,*17S* diastereomeric relationship over the *11R*,*17S*/*11S*,*17R* relationship. In addition, compound **7** was proposed to arise from reduction of the C-11 ketone group of the known co-metabolite **8**. If this proposed biogenetic relationship is correct, the transformation would not affect the terminal stereogenic center corresponding to C-17, providing supplementary support for an R configuration at C-17. On the basis of the combined DP4+ and biogenetic considerations (see Section 2.3), the *11R*,*17R* stereoisomer was regarded as the most plausible candidate. Nevertheless, this assignment remains supportive and speculative rather than definitive, because the proposed biogenetic relationship has not been experimentally verified and does not independently establish the configuration at C-11. Accordingly, the relative configuration of **7** was tentatively assigned as 11R*,17R*, whereas its absolute configuration remains undetermined (Figure 2c and Table S12).

The structures of the known compounds were identified as (*R*)-*cis*-resorcylide (**1**) (Figures S1 and S2 and Table S1) [25], dihydroresorcylide (**2**) (Figures S3 and S4 and Table S2) [26], penicimenolide B (**3**) (Figures S5 and S6 and Table S3) [19], 6,8-dihydroxy-3-[(8*R*)-8-hydroxy-2-oxononyl]-1*H*-2-benzopyran-1-one (**8**) (Figures S29 and S30 and Table S8) [26], 6,8-dihydroxy-3-[(1*E*,6*S*)-6-hydroxy-1-hepten-1-yl]-1*H*-2-benzopyran-1-one (**9**) (Figures S31 and S32 and Table S9) [26], and 6,8-dihydroxy-3-[(1*E*)-6-oxo-1-hepten-1-yl]-1*H*-2-benzopyran-1-one (**10**) (Figures S33 and S34 and Table S10) [23] by careful comparison of their spectroscopic and chiroptical data with those reported in the literature.

### 2.2. α-Glucosidase Inhibitory Activity and Enzyme Kinetic Analysis

Following the isolation and structural elucidation of compounds **1**–**10**, selected metabolites were evaluated for α-glucosidase inhibition, as oxygenated aromatic polyketides, particularly isocoumarin derivatives, have been reported as α-glucosidase inhibitors [14,27]. Since compound **1** was found to undergo nonenzymatic conversion into compound **9**, any inhibitory response observed for **1** in the absence of simultaneous chemical monitoring could not be unequivocally attributed to the intact macrolide. Therefore, no specific bioactivity conclusion was drawn for compound **1**, and the quantitative activity comparison presented here was restricted to the isocoumarin derivatives **7**–**10**. Compounds **7**–**10** share a closely related isocoumarin-type scaffold but differ in the length and oxidation state of their side chains, allowing a limited comparison of their inhibitory effects.

**Figure 3:**
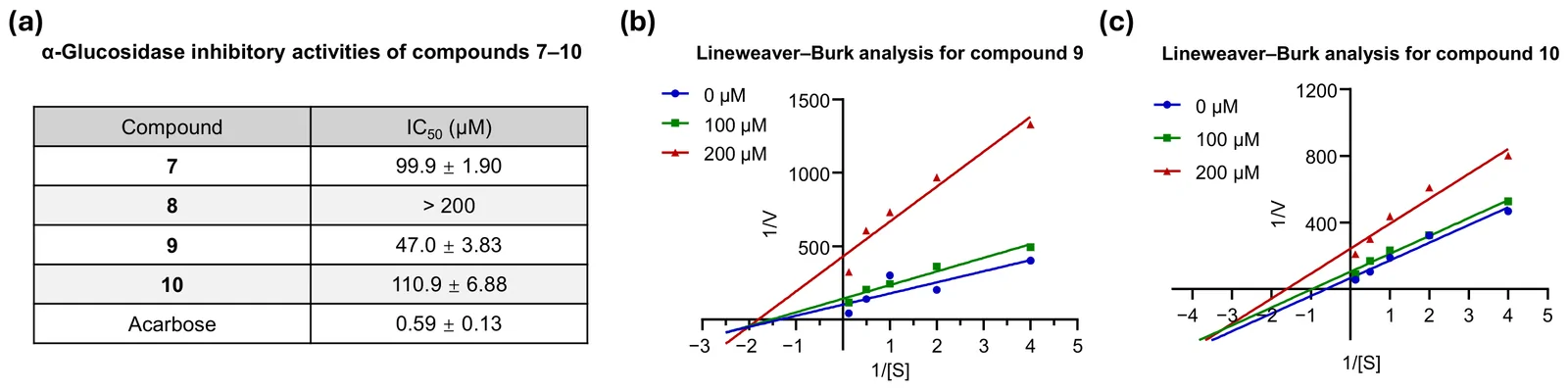
α-Glucosidase inhibitory activities and enzyme kinetic analysis of compounds **7**–**10**. (**a**) IC_50_ values of compounds **7**–**10** against α-glucosidase, with acarbose used as the positive control; (**b**) Lineweaver–Burk plots for the inhibition of α-glucosidase by compound **9** at different inhibitor concentrations; (**c**) Lineweaver–Burk plots for the inhibition of α-glucosidase by compound **10** at different inhibitor concentrations.

Due to the limited quantities of the isolated metabolites, representative resorcylic acid lactones and isocoumarin derivatives with sufficient yields were first subjected to preliminary α-glucosidase inhibitory screening at 200 μM. Among them, compound **9** showed the most pronounced inhibitory effect, with an inhibition rate of 96.00%, and was therefore selected for further evaluation together with related isocoumarin analogues. As shown in Figure 3a, compounds **7**–**10** displayed different levels of α-glucosidase inhibitory activity. Compound **9** exhibited the strongest activity, with an IC_50_ value of 47.0 ± 3.83 μM, followed by compound **7** with an IC_50_ value of 99.9 ± 1.90 μM. Compound **10** showed weaker inhibition, with an IC_50_ value of 110.9 ± 6.88 μM, whereas compound **8** was almost inactive at the tested concentration, with an IC_50_ value of >200 μM. All tested compounds were substantially less potent than the positive control acarbose (IC_50_ 0.59 ± 0.134 μM). These results indicate that compounds **7**–**10** exhibit measurable but limited α-glucosidase inhibition under the tested conditions.

Within the tested isocoumarin series **7**–**10**, compound **9** showed the strongest inhibition. The differences observed among these compounds suggest that side-chain length and oxidation state may influence their inhibitory effects. Although the limited number of compounds precluded a definitive structure–activity relationship analysis, the observed differences provide preliminary information on the effects of side-chain length and oxidation state within this isocoumarin series.

To further clarify the inhibition modes of compounds **9** and **10**, enzyme kinetic studies were performed using Lineweaver–Burk double-reciprocal plots. For compound **9**, the lines obtained at different inhibitor concentrations intersected approximately on the x-axis, while the y-intercept increased with increasing inhibitor concentration (Figure 3b), indicating that *V*_max_ decreased whereas *K*_m_ remained nearly unchanged. These results suggested that compound **9** inhibited α-glucosidase in a noncompetitive manner. In contrast, the Lineweaver–Burk plots of compound **10** intersected in the third quadrant (Figure 3c), indicating that both *K*_m_ and *V*_max_ were affected by increasing inhibitor concentrations. Therefore, compound **10** was characterized as a mixed-type α-glucosidase inhibitor.

### 2.3. Proposed Biosynthetic Pathway and Chemical Transformation

To further understand the origins and structural relationships of these metabolites, genome mining and chemical transformation studies were conducted to investigate the biosynthetic and post-biosynthetic diversification of compounds **1**–**10**.

**Figure 4:**
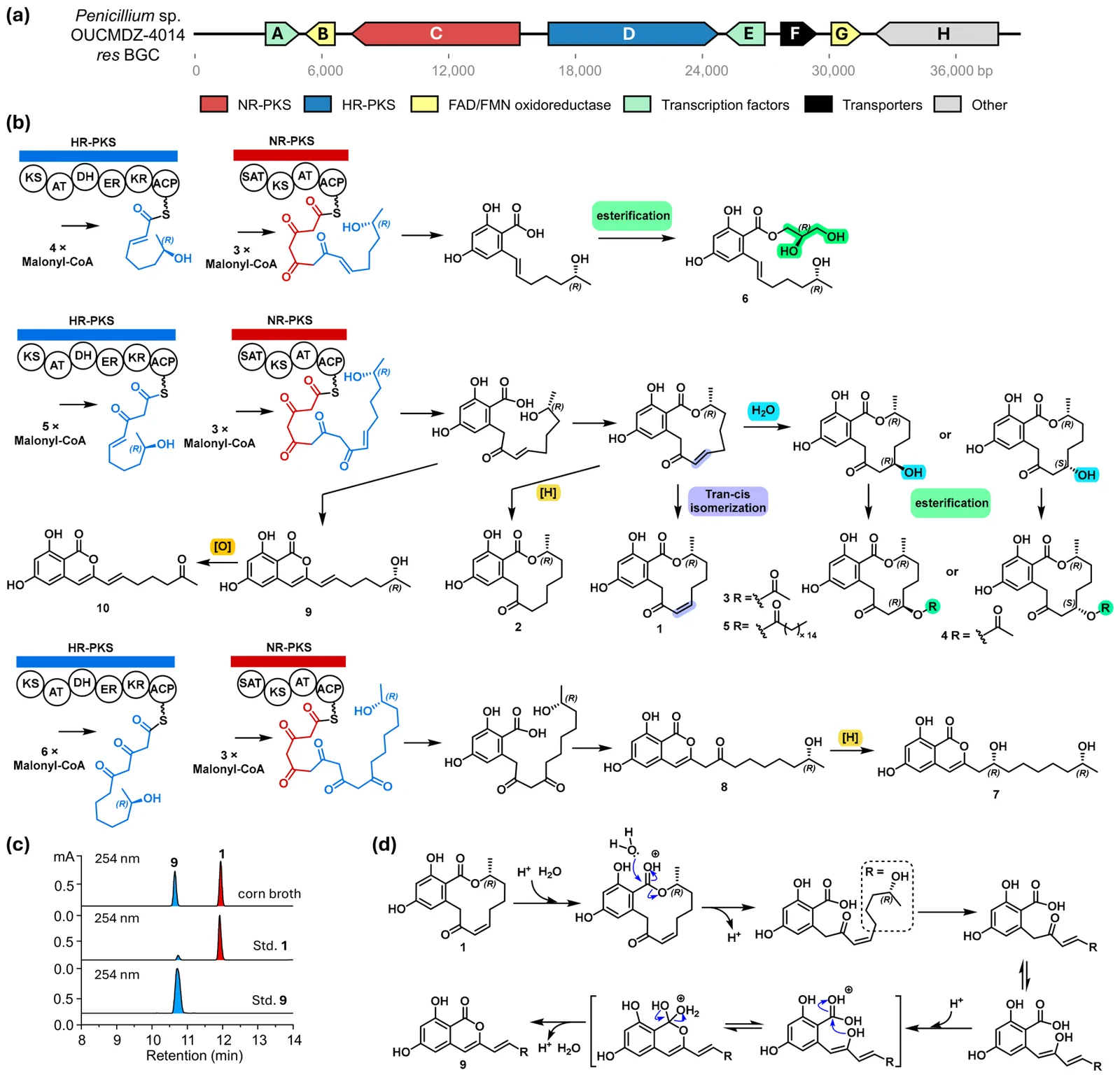
Proposed biosynthetic pathway and chemical transformation of compounds **1**–**10**. (**a**) Organization of the putative *res* biosynthetic gene cluster in *Penicillium* sp. OUCMDZ-4014; (**b**) proposed biosynthetic pathway for compounds **1**–**10** involving the collaborative action of HR-PKS and NR-PKS, followed by lactonization, esterification, oxidation, reduction, double-bond isomerization, hydration, and cyclization; (**c**) UPLC traces at 254 nm for the chemical transformation experiment between compounds **1** and **9** under simulated culture-medium conditions, compared with authentic standards of compounds **1** and **9**; (**d**) proposed mechanism for the post-biosynthetic conversion of RAL macrolide **1** to isocoumarin derivative **9**.

Genome analysis revealed a putative *res* biosynthetic gene cluster that may be associated with the production of these resorcylic acid lactone-derived metabolites (Figure 4a). This cluster contains one NR-PKS gene and one HR-PKS gene, together with genes encoding FAD/FMN-dependent oxidoreductases, transcription factors, a transporter, and other auxiliary proteins. Such a gene organization is consistent with the collaborative HR-PKS/NR-PKS biosynthetic logic commonly involved in the assembly of fungal resorcylic acid lactone-type polyketides, suggesting that the *res* BGC may participate in the biosynthesis of compounds **1**–**10** [26].

To further characterize the functional features of the core biosynthetic enzymes, domain analysis was performed using HMMER against the Pfam database (Table S22). The HR-PKS, *resD*, was predicted to contain KS-AT-DH-ER-KR-ACP domains, indicating its possible role in generating reduced aliphatic polyketide chains with different chain lengths and reduction states. The NR-PKS, *resC*, was predicted to contain SAT-KS-AT-ACP domains and may be responsible for construction of the aromatic polyketide core. Based on the structural features of compounds **1–10** and the functional annotation of the *res* BGC, a plausible biosynthetic pathway was proposed (Figure 4b). In this pathway, the HR-PKS and NR-PKS may collaboratively generate a series of ring-opened resorcylic acid-type intermediates, which are subsequently diversified through lactonization, esterification, oxidation, reduction, double-bond isomerization, and cyclization to afford RALs and isocoumarin derivatives.

In the first branch (Figure 4b), a shorter reduced polyketide fragment assembled from four C_2_ units may be coupled with an NR-PKS-derived aromatic acid intermediate, followed by esterification to give the ring-opened ester derivative **6**. This branch could account for the aromatic acid unit, alkenyl side chain, and polyhydroxylated short-chain ester fragment observed in **6**.

The second branch is proposed to represent the major route for the formation of this metabolite family (Figure 4b). A longer aliphatic chain assembled from five C_2_ units may be connected to the NR-PKS-derived aromatic polyketide core to generate a ring-opened resorcylic acid intermediate. Subsequent macrolactonization of this intermediate would afford compound **1**, which could then be reduced to give compound **2**. After double-bond isomerization, hydration or hydroxylation, and further esterification, compounds **3**–**5** could be generated. Compounds **3** and **4** may be generated as C-11 epimers through a Michael-type addition involving compound **1**, either by direct addition of an acetate equivalent or through a stepwise process involving initial hydration followed by acetylation. This proposed biogenetic relationship provides additional support for their epimeric relationship but remains to be experimentally verified. Notably, the same ring-opened intermediate may also undergo intramolecular esterification/cyclization to form the isocoumarin derivative **9**, which could be further oxidized to yield compound **10**. Therefore, compounds **9** and **10** are not proposed as products of an independent pathway outside the RAL branch, but rather as derivatives associated with the second biosynthetic branch. In particular, compound **9** may arise either from cyclization of a ring-opened intermediate in this branch or, at least in part, from post-biosynthetic nonenzymatic conversion of macrolactone **1** under culture-medium conditions.

The third branch may lead to the formation of the longer-chain isocoumarin derivatives **7** and **8** (Figure 4b). In this route, a long-chain polyketide intermediate assembled from six C_2_ units may undergo cyclization to form the ketone-containing isocoumarin skeleton of **8**. Compound **7** may subsequently arise from stereoselective reduction of the C-11 ketone group of co-metabolite **8**. If this proposed transformation occurs without modification of the terminal stereogenic center, the reported *R* configuration of the corresponding stereocenter in **8** would provide supplementary biogenetic support for a C-17*R* configuration in **7**. However, this proposal remains to be experimentally verified and cannot independently establish the configuration at C-11. Thus, variations in the chain length and oxidation state generated by the HR-PKS, together with subsequent redox modifications and different cyclization patterns, may collectively contribute to the structural diversity of this metabolite family.

To further investigate the relationship between the RAL macrolide and isocoumarin scaffolds, a chemical transformation experiment was performed to examine whether compound **1** could spontaneously transform into compound **9** under culture-medium conditions (Figure 4c). HPLC analysis showed that, after incubation of compound **1** in the culture medium, a new peak with the same retention time as authentic compound **9** appeared, indicating that compound **1** is chemically capable of undergoing nonenzymatic conversion into **9** under culture-medium conditions.

Notably, trace amounts of compound **9** were also detected after concentration of purified compound **1**, indicating that the transformation may additionally occur during sample processing. Therefore, compound **9** detected in the fungal extract may derive from both in-culture and processing-associated conversion, although their relative contributions cannot presently be quantified. Taken together, these observations establish the intrinsic chemical reactivity of compound **1** toward the nonenzymatic formation of **9** and support post-biosynthetic chemical transformation as a plausible contributor to the structural diversification of this metabolite family.

Based on these observations, a plausible mechanism for the conversion of compound **1** into compound **9** was further proposed (Figure 4d). In the presence of water and protons, the macrolactone bond of **1** may first undergo proton-assisted hydrolysis to generate a ring-opened carboxylic acid intermediate. Subsequent proton transfer, intramolecular cyclization, and dehydration/rearrangement could gradually construct the isocoumarin core, ultimately affording compound **9**. Therefore, the observed conversion of compound **1** into compound **9** supports the chemical feasibility of a post-biosynthetic, nonenzymatic transformation from an RAL macrolide to an isocoumarin derivative and reveals an additional route by which fungal polyketide diversity may be generated.

## 3. Conclusions

In this study, an HSQC NMR- and DeepSAT-assisted prioritization strategy was applied to investigate the aromatic polyketides produced by the marine-associated polar fungus *Penicillium* sp. OUCMDZ-4014. Ten structurally related resorcylic acid lactone-derived polyketides were isolated, encompassing RAL macrolides, ring-opened esters, and isocoumarins, among which compounds **4**–**7** were identified as new metabolites. These findings expand the chemical diversity of RAL-derived polyketides from polar fungi and illustrate the structural diversity associated with a single fungal strain and the close relationships among these distinct scaffolds. Compounds **7**–**10** exhibited measurable α-glucosidase inhibition, with compound **9** showing the strongest effect within the tested series, although their overall potencies were limited. Genome mining and domain analysis revealed a putative *res* biosynthetic gene cluster containing HR-PKS, NR-PKS, and tailoring-enzyme genes, supporting a collaborative HR-PKS/NR-PKS model for the generation of polyketide intermediates with different chain lengths and oxidation states and their subsequent diversification through lactonization, esterification, redox tailoring, and cyclization. Importantly, compound **1** was experimentally shown to undergo nonenzymatic conversion into compound **9** under simulated culture-medium conditions, while trace conversion was also observed during concentration. This transformation establishes the chemical feasibility of conversion from the RAL macrolide scaffold to the isocoumarin scaffold and highlights post-biosynthetic chemical transformation as an additional contributor to the observed diversity of fungal polyketides. Further genetic validation of the proposed pathway and quantitative investigation of the transformation under fermentation and processing conditions will help refine the biosynthetic and chemical relationships within this metabolite family.

## 4. Materials and Methods

### 4.1. General Experimental Procedures

NMR spectra were recorded on BRUKER AVANCE NEO 500 MHz (Bruker, Karlsruhe, Germany) with TMS as the internal standard. HR-ESI-MS spectra data were gathered on a MaXis quadrupole time-of-flight mass spectrometer (Waters, Milford, MA, USA). Optical rotations were measured with a JASCO P-1020 automatic polarimeter (JASCO, Tokyo, Japan) with MeOH as the solvent. UV spectra were recorded on Nano Drop One Microvolume UV-Vis Spectrophotometer (Thermo Fisher Scientific Inc., Waltham, MA, USA). HPLC purification was performed on Waters HPLC equipped with a DAD detector, using an ODS column (YMC-pack ODS-A, 10 × 250 mm, 5 µm, 3 mL/min). Silica gel (100–200 and 200–300 mesh) and Sephadex LH-20 for column chromatography were purchased from Qingdao Marine Chemical Group Co. (Qingdao, China) and Pharmacia Biotech AB (Uppsala, Sweden), respectively. All solvents used in CC and HPLC were of analytical grade (Tianjin Damao Chemical Plant, Tianjin, China) and chromatographic grade (MERDA, Beijing, China), respectively.

### 4.2. Fungal Materials

The fungus *Penicillium* sp. OUCMDZ-4014 was isolated from Antarctic moss (GenBank accession No. PV849063). The strain was deposited in the Zhu’s Laboratory, Ocean University of China. The working strain was cultured on potato dextrose agar (PDA) slants and stored at 4 °C.

### 4.3. DeepSAT-Assisted HSQC Analysis

To rapidly evaluate the chemical classes of the metabolites in the crude fractions, ^1^H–^13^C HSQC spectra were analyzed using the DeepSAT web platform (https://deepsat.ucsd.edu/, accessed on 23 May 2025) [17]. The HSQC cross-peak lists of the selected fractions were generated from the processed NMR spectra and used as input data for DeepSAT analysis. The ^1^H and ^13^C chemical shift coordinates of the observed HSQC correlations were uploaded to the DeepSAT server according to the instructions provided on the website.

### 4.4. Fermentation, Extraction, and Purification

The strain *Penicillium* sp. OUCMDZ-4014 was cultivated on corn medium under static conditions at 16 °C for 33 days in 100 bags. The fermented cultures were extracted with EtOAc to afford an oily crude extract (129.3 g).

The crude extract was subjected to vacuum liquid chromatography (VLC) over silica gel using petroleum ether (100%) and MeOH–CH_2_Cl_2_ (0–100%) as eluents to yield six fractions (Fr. 1–Fr. 6). Fr. 1 was further separated into ten subfractions (Fr. 1-1–Fr. 1-10) by reversed-phase silica gel column chromatography with a stepwise gradient of MeOH–H_2_O (10–100%). These fractions were concentrated and analyzed by LC-MS. Fr. 1-5 was applied to a Sephadex LH-20 column eluting with MeOH and further purified by semipreparative HPLC on an ODS column (YMC-Pack ODS-A, 10 × 250 mm, 5 μm, 3.0 mL/min) eluting with 43% MeCN–H_2_O to yield compound **7** (1.7 mg, *t*_R_ 35.2 min), compound **10** (1.7 mg, *t*_R_ 39.1 min), compound **4** (17.5 mg, *t*_R_ 40.3 min), and compound **3** (13.9 mg, *t*_R_ 42.1 min), together with subfraction Fr. 1-5-4. Fr. 1-5-4 was further purified by semipreparative HPLC on the same ODS column eluting with 50% MeCN–H_2_O to afford compound **1** (14.2 mg, *t*_R_ 15.6 min). Fr. 1-10 was applied to a Sephadex LH-20 column eluting with MeOH and further purified by semipreparative HPLC on the same ODS column eluting with 90% MeCN–H_2_O to give a diastereomeric mixture, which was subsequently resolved by semipreparative HPLC on a chiral column (Chiral MD(2)-RH, 4.6 × 250 mm, 5 μm, 1.0 mL/min) eluting with 86% MeCN–H_2_O to yield compound **5** (1.0 mg, *t*_R_ 36.5 min).

Fr. 3 was separated into ten subfractions (Fr. 3-1–Fr. 3-10) by reversed-phase silica gel column chromatography with a stepwise gradient of MeOH–H_2_O (10–100%). These fractions were concentrated and analyzed by LC-MS. Fr. 3-10 was applied to a Sephadex LH-20 column eluting with MeOH and further purified by semipreparative HPLC on the same ODS column eluting with 22% MeCN–H_2_O to yield compound **6** (2.5 mg, *t*_R_ 26.3 min).

Fr. 5 was separated into 19 subfractions (Fr. 5-1–Fr. 5-19) by reversed-phase silica gel column chromatography with a stepwise gradient of MeOH–H_2_O (10–100%). These fractions were concentrated and analyzed by LC-MS. Fr. 5-12 was applied to a Sephadex LH-20 column eluting with MeOH and further purified by semipreparative HPLC on the same ODS column eluting with 32% MeCN–H_2_O to afford compound **2** (2.8 mg, *t*_R_ 52.4 min). Fr. 5-14 was applied to a Sephadex LH-20 column eluting with MeOH and further purified by semipreparative HPLC on the same ODS column eluting with 41% MeCN–H_2_O to afford compound **8** (1.1 mg, *t*_R_ 34.7 min). Fr. 5-17 was applied to a Sephadex LH-20 column eluting with MeOH and further purified by semipreparative HPLC on the same ODS column eluting with 43% MeCN–H_2_O to yield compound **9** (3.5 mg, *t*_R_ 32.3 min).

### 4.5. Structural Elucidation of Compounds

Compound **4**: colorless oily solid; [α]^20^_D_ −24.4 (c 0.1, MeOH); UV(MeOH) λ_max_ (log ε) 215 (1.83), 263 (0.93), 302 (0.51) nm; ECD (1.43 mM, MeOH) *λ*_max_ (*Δε*) 228 (+5.43), 243 (−1.54), 266 (+1.50), 309 (−1.29) nm; ^1^H and ^13^C NMR, see Table S4; HRESIMS *m*/*z* 351.1435 (C_18_H_23_O_7_^+^ calcd 351.1438).

Compound **5**: pale-yellow oily solid; [α]^20^_D_ +1.1(c 0.1,MeOH); UV (MeOH) *λ*_max_ (log *ε*) 215 (1.24), 262 (0.72), 302 (0.35) nm; ECD (0.92 mM, MeOH) *λ*_max_ (*Δε*) 220 (−2.61), 263 (+7.58), 306 (−2.09) nm; IR(KBr) *ν*_max_: 3750, 3650, 3565, 3446, 2925, 2853, 1715, 1688, 1646, 1541, 1456, 1385, 1260, 1206 cm^−1^; ^1^H and ^13^C NMR, see Table S5; HRESIMS *m*/*z* 547.3621 (C_32_H_51_O_7_^+^ calcd 547.3629).

Compound **6**: colorless oily solid; [*α*]_D_^30.4^ +6.5 (*c* 0.1, MeOH); UV (MeOH) *λ*_max_ (log *ε*) 237 (1.97), 273 (1.0), 308 (0.38) nm; ECD (1.46 mM, MeOH) λ_max_ (Δε) 213 (−0.77), 263 (+0.36), 314 (−0.18) nm; IR(KBr) *ν*_max_: 3446, 2918, 2850, 1650, 1259, 1168, 1058 cm^−1^; ^1^H and ^13^C NMR, see Table S6; HRESIMS *m*/*z* 341.1594 (C_17_H_24_O_7_^+^ calcd 341.1595).

Compound **7**: yellow solid; [α]^20^_D_ +2.7(c 0.1, MeOH); UV(MeOH) λ_max_ (log ε) 246 (2.03), 329 (0.33) nm; ECD (1.48 mM, MeOH) λ_max_ (Δε) 205 (+1.83), 225 (+0.85), 232 (+1.14), 241 (+12.58), 247 (+11.56), 289 (+2.92), 315 (+1.58) nm; IR (KBr) *v*_max_ 3420, 2928, 2853, 1681, 1617, 1504, 1238, 1174 cm^−1^; ^1^H and ^13^C NMR, see Table S7; HRESIMS *m*/*z* 337.1653 (C_18_H_25_O_6_^+^ calcd 337.1646).

### 4.6. Quantum Chemical Calculations

#### 4.6.1. Electronic Circular Dichroism (ECD) Calculations

The absolute configuration of compound **5** was evaluated by comparison of its experimental ECD spectrum with quantum-chemically calculated ECD spectra of the candidate stereoisomers. Conformational searches were conducted in Spartan 14 using the MMFF force field within an energy window of 10 kJ/mol, and the calculated ECD spectra of low-energy conformers were matched with the experimental data to assign absolute configurations. The detailed process was described in experimental section of Supplemental Material.

#### 4.6.2. ^13^C NMR Calculations and DP4+ Analysis

The relative stereochemical relationships of compounds **6** and **7** were evaluated by comparing their experimental ^13^C NMR data with calculated chemical shifts, followed by DP4+ probability analysis. Because conventional NMR chemical shifts measured in an achiral medium cannot distinguish enantiomeric pairs, the DP4+ results were not used independently to establish absolute configurations. Conformational searches were initially performed in Spartan’14 using the MMFF force field within an energy window of 10 kJ/mol. The obtained low-energy conformers were further optimized in the gas phase at the B3LYP/6-31G(d) level. The magnetic shielding tensors were then calculated using the gauge-independent atomic orbital (GIAO) method with the NMR keyword in Gaussian 16W at the mPW1PW91/6-311+G(d,p) level.

The calculated shielding constants of each conformer were converted into chemical shift values by comparison with the calculated shielding constant of the reference compound. The overall calculated chemical shifts were obtained by Boltzmann weighting according to the relative free energies of the conformers. The resulting ^13^C NMR chemical shifts were then scaled and compared with the experimental values. Finally, DP4+ probability analysis was performed to evaluate the relative agreement of the candidate diastereomeric relationships with the experimental NMR data.

### 4.7. α-Glucosidase Inhibitory Assay

#### 4.7.1. Preliminary Screening

The α-glucosidase inhibitory activity was evaluated using a spectrophotometric method with p-nitrophenyl α-D-glucopyranoside (PNPG) as the substrate. α-Glucosidase and PNPG were prepared in phosphate buffer (0.1 M, pH 6.8) at concentrations of 0.2 U/mL and 2.5 mM, respectively. Test compounds and acarbose, used as the positive control, were dissolved in DMSO.

For preliminary screening, representative compounds with sufficient quantities were tested at a final concentration of 200 μM. In a 96-well plate, 10 μL of the sample solution and 100 μL of α-glucosidase solution were added to each well and incubated at 37 °C for 15 min. Subsequently, 40 μL of PNPG solution was added to initiate the reaction, while phosphate buffer was added instead of PNPG in the blank wells. After incubation at 37 °C for another 15 min, the absorbance was measured at 405 nm. The inhibition rate was calculated according to the following equation:

Inhibition (%) = [1 − (A_sample_ − A_sample blank_)/(A_control_ − A_blank_)] × 100%

All experiments were performed in triplicate.

#### 4.7.2. IC_50_ Determination

Compounds showing inhibitory activity in the preliminary screening, together with related isocoumarin analogues, were further evaluated for their IC_50_ values. The test compounds were serially diluted with DMSO to prepare six concentrations. The assay procedure was the same as described above. Briefly, 10 μL of each diluted sample solution and 100 μL of α-glucosidase solution were mixed in a 96-well plate and preincubated at 37 °C for 15 min. Then, 40 μL of PNPG solution was added to initiate the enzymatic reaction. After incubation at 37 °C for 15 min, the absorbance was recorded at 405 nm. Acarbose was used as the positive control. The IC_50_ values were calculated from dose–response curves and expressed as mean ± SD of three independent experiments.

#### 4.7.3. Enzyme Kinetic Analysis

The inhibition modes of compounds **9** and **10** against α-glucosidase were investigated using PNPG as the substrate. The kinetic assays were performed at five PNPG concentrations ranging from 0.25 to 8 mM in the absence or presence of inhibitors at two concentrations, 100 and 200 μM. The final concentration of α-glucosidase was 0.5 U/mL.

The absorbance at 405 nm was monitored every minute for 20 min. The linear portion of the reaction progress curve from 5 to 15 min was used to calculate the initial velocity (*V*_0_). Lineweaver–Burk double-reciprocal plots were generated by plotting 1/[*S*] against 1/*V*_0_ at each inhibitor concentration. The apparent kinetic parameters *K*_m_ and *V*_max_ were determined from the plots, and the inhibition types were assigned by comparing the changes in *K*_m_ and *V*_max_ with increasing inhibitor concentrations.

### 4.8. Bioinformatic Analysis

Bioinformatic analysis was performed to identify the putative biosynthetic gene cluster associated with compounds **1**–**10** in *Penicillium* sp. OUCMDZ-4014. Core biosynthetic enzymes from known resorcylic acid lactone-type pathways were used as query sequences for local homology searches. When the genome assembly was used as the search database, tBLASTn was employed to locate homologous genomic regions; when predicted protein sequences were used as the database, BLASTp was used for protein-level comparison. tBLASTn and BLASTp searches were performed using NCBI BLAST+ version 2.17.0. The genomic region containing homologues of the core PKS genes was selected as the candidate biosynthetic locus.

The candidate region was further analyzed using antiSMASH and DeepBGC to predict secondary metabolite biosynthetic potential and to define the putative cluster boundaries [28,29]. Coding sequences within the candidate region were predicted and refined using AUGUSTUS [30], with the closest related fungal species used as the prediction model. The protein sequences encoded by the predicted genes were analyzed using HMMER against the Pfam database to identify conserved domains and infer possible enzymatic functions [31,32].

The functions of genes within the candidate cluster were assigned based on conserved domain architecture, sequence homology, biosynthetic logic, and the structural features of the isolated metabolites. The putative *res* biosynthetic gene cluster was visualized using DNA Feature Viewer [33], and a plausible biosynthetic pathway for compounds **1**–**10** was proposed based on the combined bioinformatic and chemical evidence.

### 4.9. Chemical Transformation from Compound **1** to Compound **9**

To investigate whether compound **1** could be converted into compound **9** under culture-medium conditions, compound **1** standard sample (1.0 mg) was added to 3 mL of autoclaved corn broth medium to simulate the fermentation environment. The mixture was stirred at room temperature for 7 days. After incubation, the reaction mixture was extracted three times with an equal volume of EtOAc. The combined EtOAc extracts were concentrated under reduced pressure, and the residue was redissolved in 1 mL of MeOH. The resulting solution was analyzed by UPLC-MS at 254 nm. The retention time of the newly generated peak was compared with those of authentic standards of compounds **1** and **9** to evaluate the chemical transformation from **1** to **9**.

## Data Availability

The original contributions presented in this study are included in the article and Supplementary Materials. Further inquiries can be directed to the corresponding authors.

## Supplementary Materials

The following supporting information can be downloaded at: https://www.mdpi.com/article/10.3390/md24080256/s1, Figures S1–S34: HRESIMS, 1D, and 2D NMR spectra of compounds **1**–**10**; Tables S1–S10: NMR spectroscopic data assignments of compounds **1**–**10**; Tables S11–S21: computational chemistry data; Table S22: domain annotation of the target biosynthetic gene cluster.
