# Supplementary material for: Resorcylic Acid Lactones and Isocoumarin Derivatives from the Marine-Associated Polar Fungus Penicillium sp. OUCMDZ-4014
Source: Mar Drugs. 2026 Jul 24;24(8):256. doi: 10.3390/md24080256 (PMC13514119; doi:10.3390/md24080256)
Supplement: Supplementary file 1 [file marinedrugs-24-00256-s001.zip › marinedrugs-4428743-supplementary.pdf]

Supplementary Materials

Resorcylic Acid Lactones and Isocoumarin Derivatives from the Marine-Associated Polar Fungus *Penicillium* sp. OUCMDZ-4014

Deng Yu [1, †], Rongtian Du [1, †], Xuehan Yu[1], Chengze An[1], Chenyang Ding[1], Jiapeng Wang[1], Weiming Zhu [1, 2, 3, *] and Yi Wang [1, 2, 3, *]

1) School of Medicine and Pharmacy, Ocean University of China, Qingdao 266003, China.

2) Laboratory for Marine Drugs and Bioproducts, Qingdao Marine Science and Technology Centre, Qingdao, 266237, China.

3) Key Laboratory of Marine Drugs, Ministry of Education of China, Ocean University of China, Qingdao 266003, China.

* Corresponding authors. E-mail: wangyi0213@ouc.edu.cn;

[#] The two authors contributed equally to this work.

This PDF file includes:

Structural Elucidation of Compounds
Figures S1 to S34
Tables. S1 to S12

Supporting Information Contents

Spectral data of all isolated compounds 1
Figure S1. ^{1}H NMR spectrum of **1** in DMSO-d_6 (400 MHz). 2
Figure S2. ^{13}C NMR spectrum of **1** in DMSO-d_6 (150 MHz). 3
Figure S3. ^{1}H NMR spectrum of **2** in DMSO-d_6 (400 MHz). 4
Figure S4. ^{13}C NMR spectrum of **2** in DMSO-d_6 (100 MHz). 5
Figure S5. ^{1}H NMR spectrum of **3** in DMSO-d_6 (400 MHz). 6
Figure S6. ^{13}C NMR spectrum of **3** in DMSO-d_6 (100 MHz). 7
Figure S7. ^{1}H NMR spectrum of **4** in DMSO-d_6 (400 MHz). 8
Figure S8. ^{13}C NMR spectrum of **4** in DMSO-d_6 (100 MHz). 9
Figure S9. HRESIMS spectrum of compound **4**. 10
Figure S10. ^{1}H NMR spectrum of **5** in DMSO-d_6 (400 MHz). 11
Figure S11. ^{13}C NMR spectrum of **5** in DMSO-d_6 (125 MHz). 12
Figure S12. HSQC spectrum of **5** in DMSO-d_6 (500 MHz, 125 MHz). 13
Figure S13. HMBC spectrum of **5** in DMSO-d_6 (500 MHz, 125 MHz). 14
Figure S14. ^{1}H-^{1}H COSY spectrum of **5** in DMSO-d_6 (500 MHz, 500 MHz). 15
Figure S15. ^{1}H-^{1}H NOESY spectrum of **5** in DMSO-d_6 (500 MHz, 500 MHz). 16
Figure S16. HRESIMS spectrum of compound **5**. 17
Figure S17. ECD spectrum of compound **5**. 18
Figure S18. ^{1}H NMR spectrum of **6** in DMSO-d_6 (400 MHz).. 19
Figure S19. ^{13}C NMR spectrum of **6** in DMSO-d_6 (125 MHz). 20
Figure S20. HSQC spectrum of **6** in DMSO-d_6 (500 MHz, 125 MHz). 21
Figure S21. HMBC spectrum of **6** in DMSO-d_6 (500 MHz, 125 MHz). 22
Figure S20. ^{1}H-^{1}H COSY spectrum of **6** in DMSO-d_6 (500 MHz, 500 MHz). 23
Figure S21. ^{1}H-^{1}H NOESY spectrum of **6** in DMSO-d_6 (500 MHz, 500 MHz). 24
Figure S22. HRESIMS spectrum of compound **6**. 25
Figure S23. ^{1}H NMR spectrum of **7** in DMSO-d_6 (400 MHz). 26
Figure S24. ^{13}C NMR spectrum of **7** in DMSO-d_6 (125 MHz). 27
Figure S25. HSQC spectrum of **7** in DMSO-d_6 (500 MHz, 125 MHz). 28
Figure S26. HMBC spectrum of **7** in DMSO-d_6 (500 MHz, 125 MHz). 29
Figure S27. ^{1}H-^{1}H COSY spectrum of **7** in DMSO-d_6 (500 MHz, 500 MHz). 30
Figure S28. HRESIMS spectrum of compound **7**. 31
Figure S29. ^{1}H NMR spectrum of **8** in DMSO-d_6 (400 MHz). 32
Figure S30. ^{13}C NMR spectrum of **8** in DMSO-d_6 (125 MHz). 33
Figure S31. ^{1}H NMR spectrum of **9** in DMSO-d_6 (400 MHz).. 34
Figure S32. ^{13}C NMR spectrum of **9** in DMSO-d_6 (100 MHz). 35
Figure S33. ^{1}H NMR spectrum of **10** in DMSO-d_6 (400 MHz).. 36
Figure S34. ^{13}C NMR spectrum of **10** in DMSO-d_6 (125 MHz). 37
Table S1. ^{1}H (400 MHz) and ^{13}C (100 MHz) NMR data for compound **1** in DMSO-d_6. 38
Table S2. ^{1}H (400 MHz) and ^{13}C (100 MHz) NMR data for compound **2** in DMSO-d_6. 39
Table S3. ^{1}H (400 MHz) and ^{13}C (100 MHz) NMR data for compound **3** in DMSO-d_6. 40
Table S4. ^{1}H (400 MHz) and ^{13}C (100 MHz) NMR data for compound **4** in DMSO-d_6. 41
Table S5. ^{1}H (400 MHz) and ^{13}C (125 MHz) NMR data for compound **5** in DMSO-d_6. 42

Table S6. ^{1}H (400 MHz) and ^{13}C (125 MHz) NMR data for compound **6** in DMSO-d_6. 43
Table S7. ^{1}H (400 MHz) and ^{13}C (125 MHz) NMR data for compound **7** in DMSO-d_6. 44
Table S8. ^{1}H (400 MHz) and ^{13}C (125 MHz) NMR data for compound **8** in DMSO-d_6. 45
Table S9. ^{1}H (400 MHz) and ^{13}C (100 MHz) NMR data for compound **9** in DMSO-d_6. 46
Table S10. ^{1}H (400 MHz) and ^{13}C (125 MHz) NMR data for compound **10** in DMSO-d_6. 47
Table S11. DP4+ probability analysis of compound **6**. 48
Table S12. DP4+ probability analysis of compound **7**. 49
Table S13. DFT-optimized structures for low-energy conformers of **5a**. 50
Table S14. DFT-optimized structures for low-energy conformers of **6a**. 51
Table S15. DFT-optimized structures for low-energy conformers of **6b**. 52
Table S16. DFT-optimized structures for low-energy conformers of **6c**. 53
Table S17. DFT-optimized structures for low-energy conformers of **6d**. 54
Table S18. DFT-optimized structures for low-energy conformers of **7a**. 55
Table S19. DFT-optimized structures for low-energy conformers of **7b**. 56
Table S20. DFT-optimized structures for low-energy conformers of **7c**. 57
Table S21. DFT-optimized structures for low-energy conformers of **7d**. 58
Table S22. Predicted conserved domains of genes in the putative *res* biosynthetic gene cluster. 59

Spectral data of all isolated compounds

Compound 1: white amorphous powder; $[\alpha]_D^{20}$ −8.7 (*c* 0.1, MeOH); UV (MeOH) λ_{max} (log ε) 215 (2.34), 263 (1.14), 302 (0.73) nm; ^{1}H and ^{13}C NMR, see Table S1; ESIMS m/z 291.85 $[M + H]^+$.

Compound 2: colorless oil; $[\alpha]_D^{20}$ +24.1 (*c* 0.1, MeOH); UV (MeOH) λ_{max} (log ε) 215 (1.58), 263 (0.78), 302 (0.39) nm; ^{1}H and ^{13}C NMR, see Table S2; ESIMS m/z 293.81 $[M + H]^+$.

Compound 3: yellow powder; $[\alpha]_D^{20}$ +19.1 (*c* 0.1, MeOH); UV (MeOH) λ_{max} (log ε) 215 (1.75), 263 (0.91), 302 (0.49) nm; ^{1}H and ^{13}C NMR, see Table S3; ESIMS m/z 349.99 $[M - H]^-$.

Compound 4: colorless oil; $[\alpha]_D^{20}$ −24.4 (*c* 0.1, MeOH); UV (MeOH) λ_{max} (log ε) 215 (1.83), 263 (0.93), 302 (0.51) nm; ECD (1.43 mM, MeOH) λ_{max} ($\Delta\varepsilon$) 228 (+5.43), 243 (−1.54), 266 (+1.50), 309 (−1.29) nm;^{1}H and ^{13}C NMR, see Table 4; ESIMS *m/z* 349.99 $[M - H]^-$ (calcd for $C_{18}H_{22}O_7$, 349.1293).

Compound 5: pale yellow oil; $[\alpha]_D^{20}$ +1.1 (*c* 0.1, MeOH); UV (MeOH) λ_{max} (log ε) 215 (1.24), 262 (0.72), 302 (0.35) nm; ECD (0.92 mM, MeOH) λ_{max} ($\Delta\varepsilon$) 220 (−2.61), 263 (+7.58), 306 (−2.09) nm; IR (KBr) ν_{max} 3750, 3650, 3565, 3446, 2925, 2853, 1715, 1688, 1646, 1541, 1456, 1385, 1260, 1206 cm^{-1}; ^{1}H and ^{13}C NMR, see Table S5; HRESIMS *m/z* 547.3621 $[M + H]^+$(calcd for $C_{32}H_{50}O_7$, 547.3629).

Compound 6: colorless oil; $[\alpha]_D^{20}$ −5.35 (*c* 0.1, MeOH); UV (MeOH) λ_{max} (log ε) 237 (1.97), 273 (1.0), 308 (0.38) nm; ECD (1.46 mM, MeOH) λ_{max} (Δε) 213 (−0.77), 263 (+0.36), 314 (−0.18) nm; IR (KBr) ν_{max} 3446, 2918, 2850, 1650, 1259, 1168, 1058 cm^{-1}; ^{1}H and ^{13}C, see Table S6; HRESIMS *m/z* 341.1594 $[M + H]^+$(calcd for $C_{17}H_{24}O_7$, 341.1595).

Compound 7: yellow powder; $[\alpha]_D^{20}$ +2.7 (*c* 0.1, MeOH); UV (MeOH) λ_{max} (log ε) 246 (2.03), 329 (0.33) nm; ECD (1.48 mM, MeOH) λ_{max} (Δε) 205 (+1.83), 225 (+0.85), 232 (+1.14), 241 (+12.58), 247 (+11.56), 289 (+2.92), 315 (+1.58) nm; IR (KBr) ν_{max} 3420, 2928, 2853, 1681, 1617, 1504, 1238, 1174 cm^{-1}; ^{1}H and ^{13}C NMR, see Table S7; HRESIMS *m/z* 337.1653 $[M + H]^+$ (calcd for $C_{18}H_{24}O_6$, 337.1646).

Compound 8: yellow oil; $[\alpha]_D^{20}$ −2.6 (*c* 0.1, MeOH); UV (MeOH) λ_{max} (log ε) 246 (2.01), 329 (0.32) nm; ^{1}H and ^{13}C NMR, see Table S8; ESIMS m/z 333.35 $[M - H]^-$.

Compound 9: white amorphous powder; $[\alpha]_D^{20}$ −11.6 (*c* 0.1, MeOH); UV (MeOH) λ_{max} (log ε) 202 (0.33), 259 (1.45), 299 (0.26), 342 (0.32) nm; ^{1}H and ^{13}C NMR, see Table S9; ESIMS m/z 289.11 $[M - H]^-$.

Compound 10: white amorphous powder; UV (MeOH) λ_{max} (log ε) 202 (0.31), 259 (1.42), 299 (0.27), 342 (0.31) nm; ^{1}H and ^{13}C NMR, see Table S10; ESIMS m/z 289.90 $[M + H]^+$.

Figure S1. ^{1}H NMR spectrum of **1** in DMSO-d_6 (400 MHz).

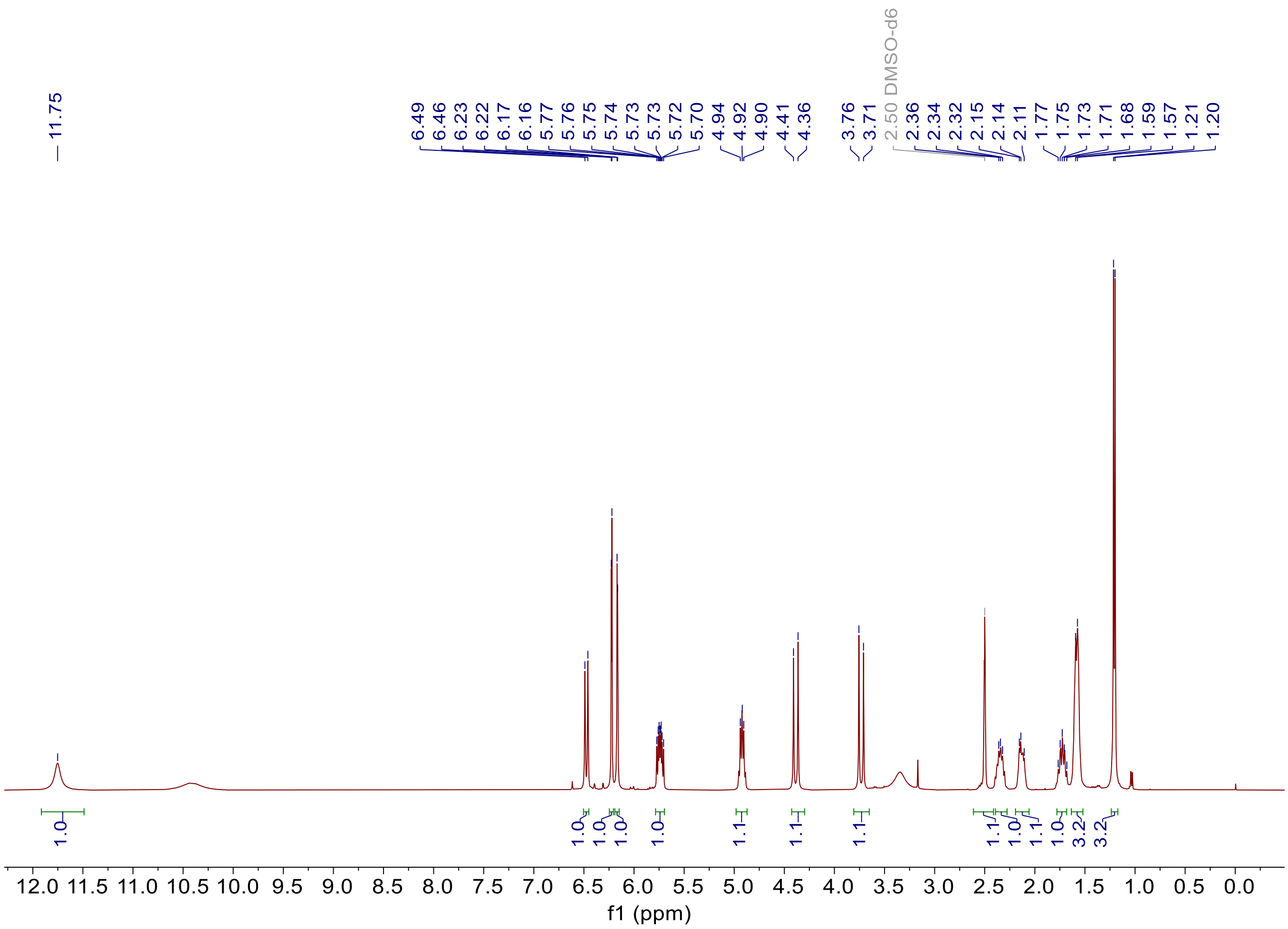

Figure S2. ^{13}C NMR spectrum of **1** in DMSO-d_6 (150 MHz).

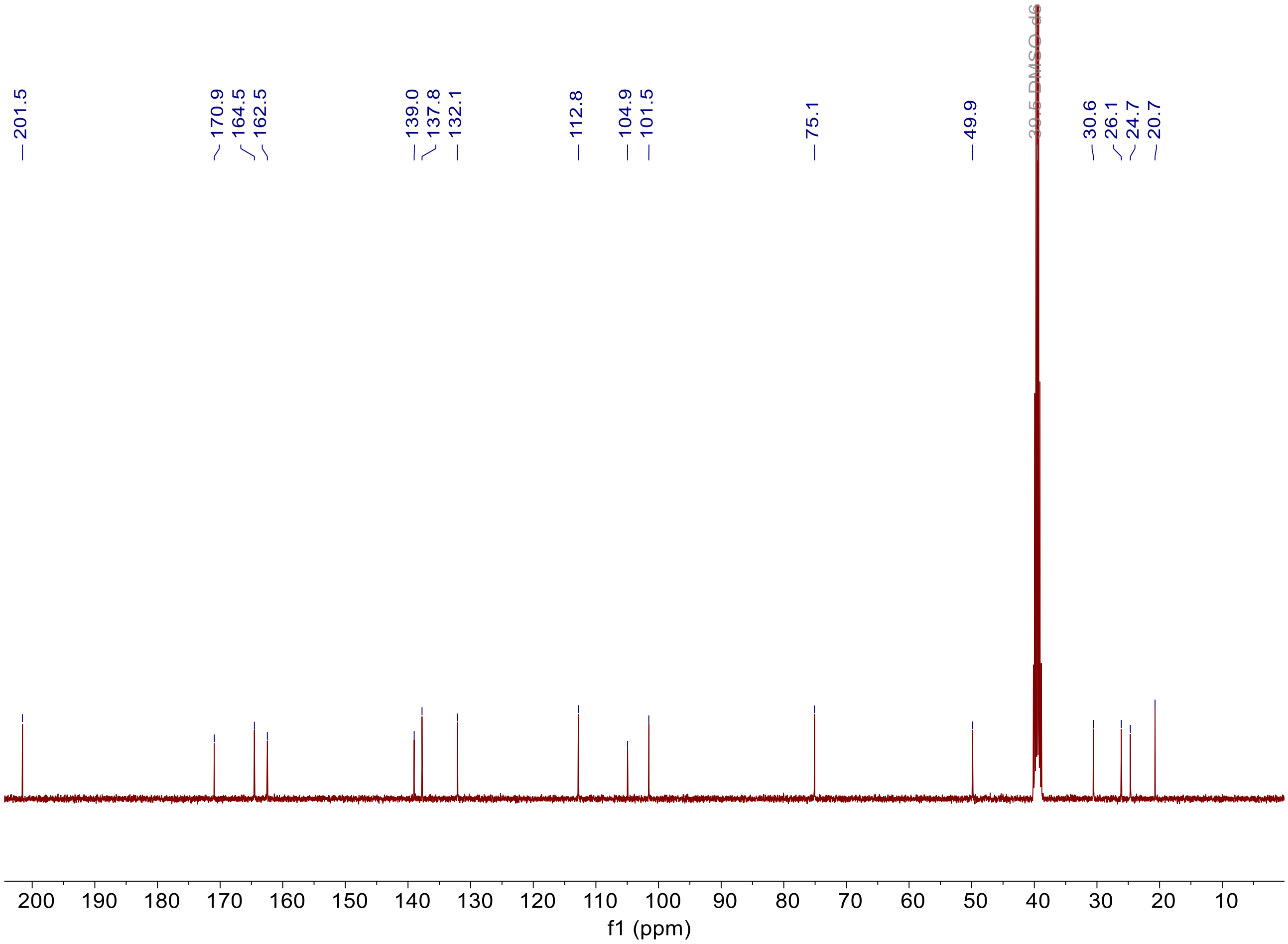

Figure S3. ¹H NMR spectrum of **2** in DMSO-d_6 (400 MHz).

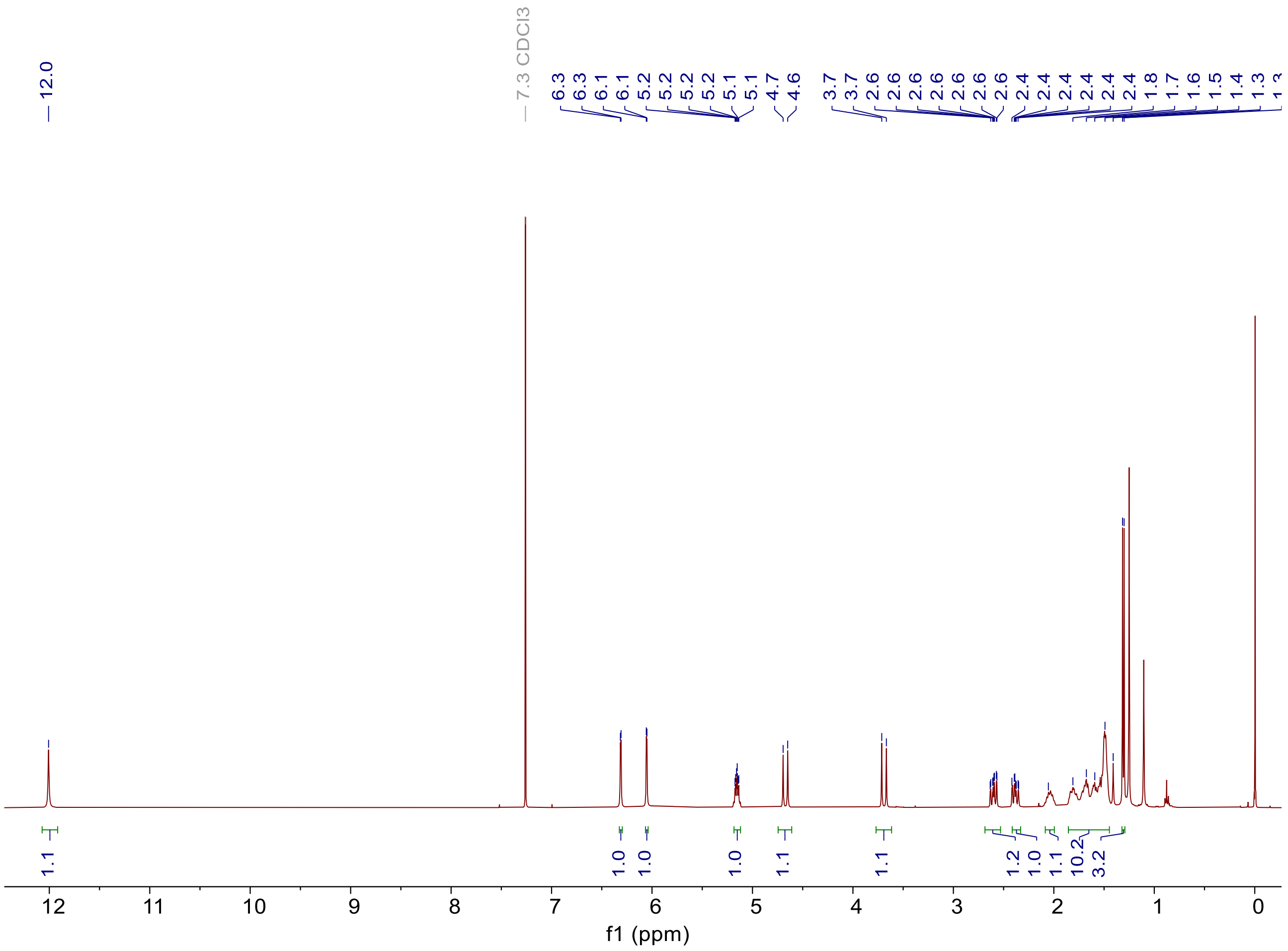

Figure S4. ^{13}C NMR spectrum of **2** in DMSO-d_6 (100 MHz).

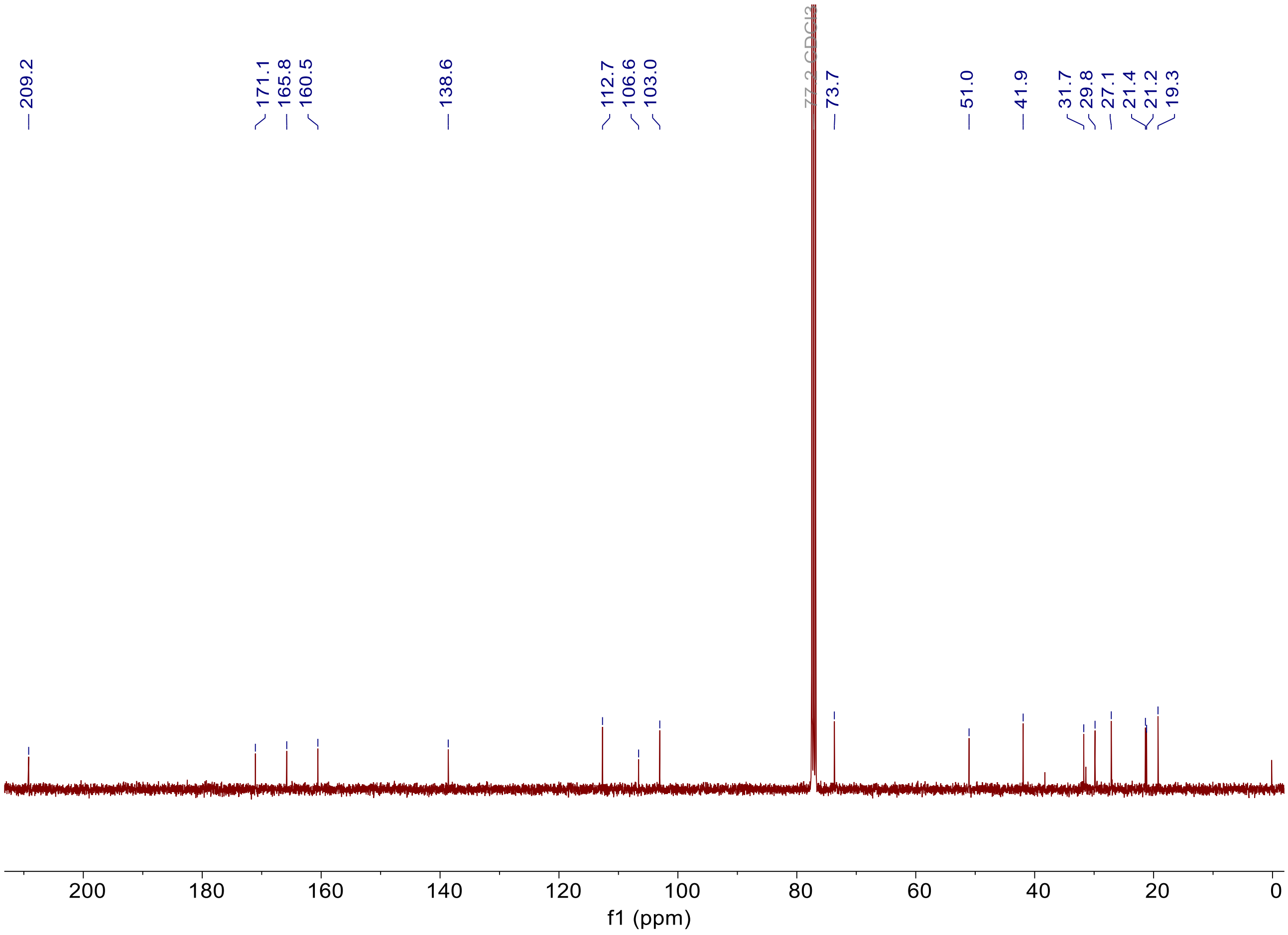

Figure S5. ^{1}H NMR spectrum of **3** in DMSO-d_6 (400 MHz).

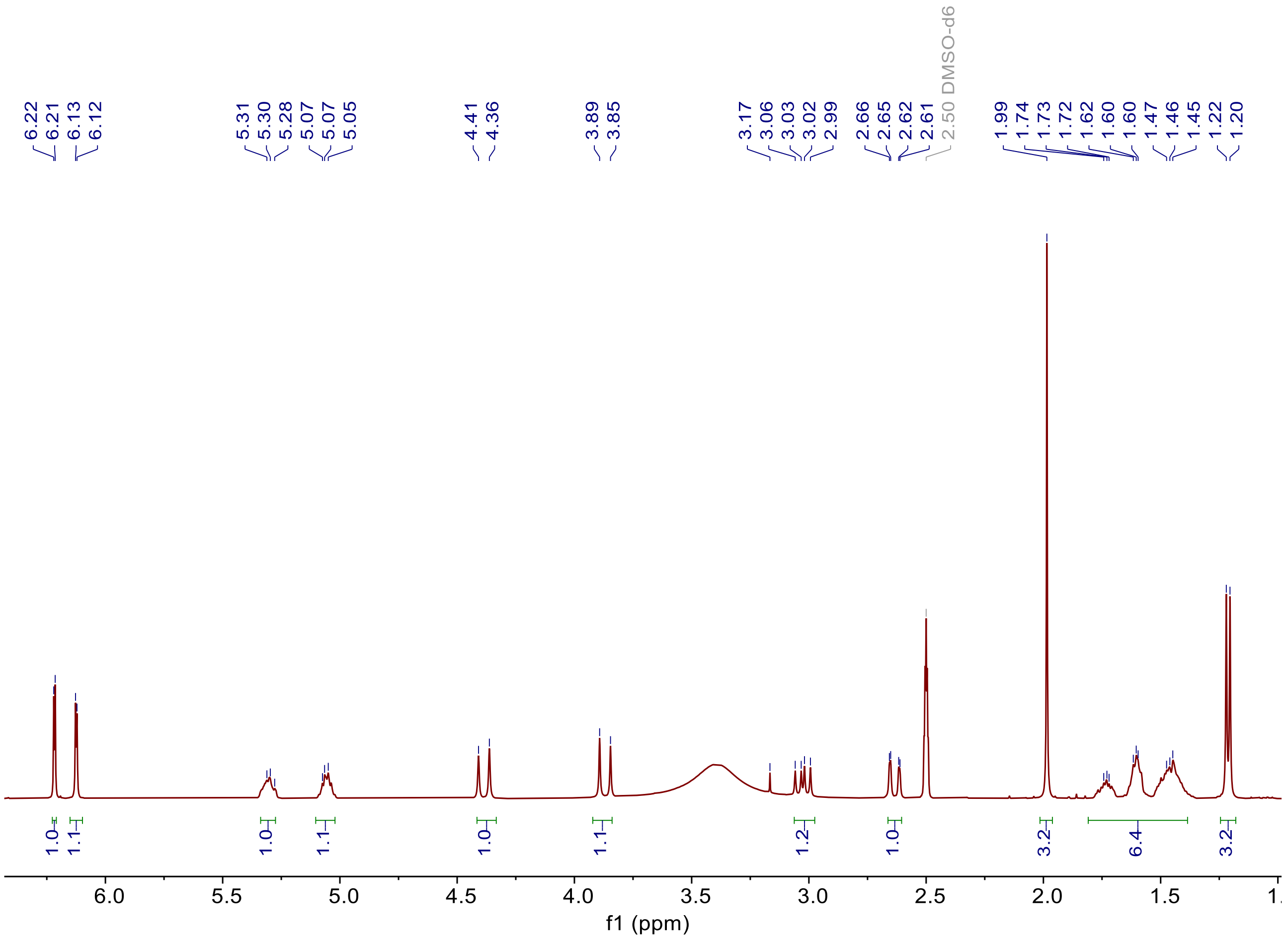

Figure S6. ^{13}C NMR spectrum of **3** in DMSO-d_6 (100 MHz).

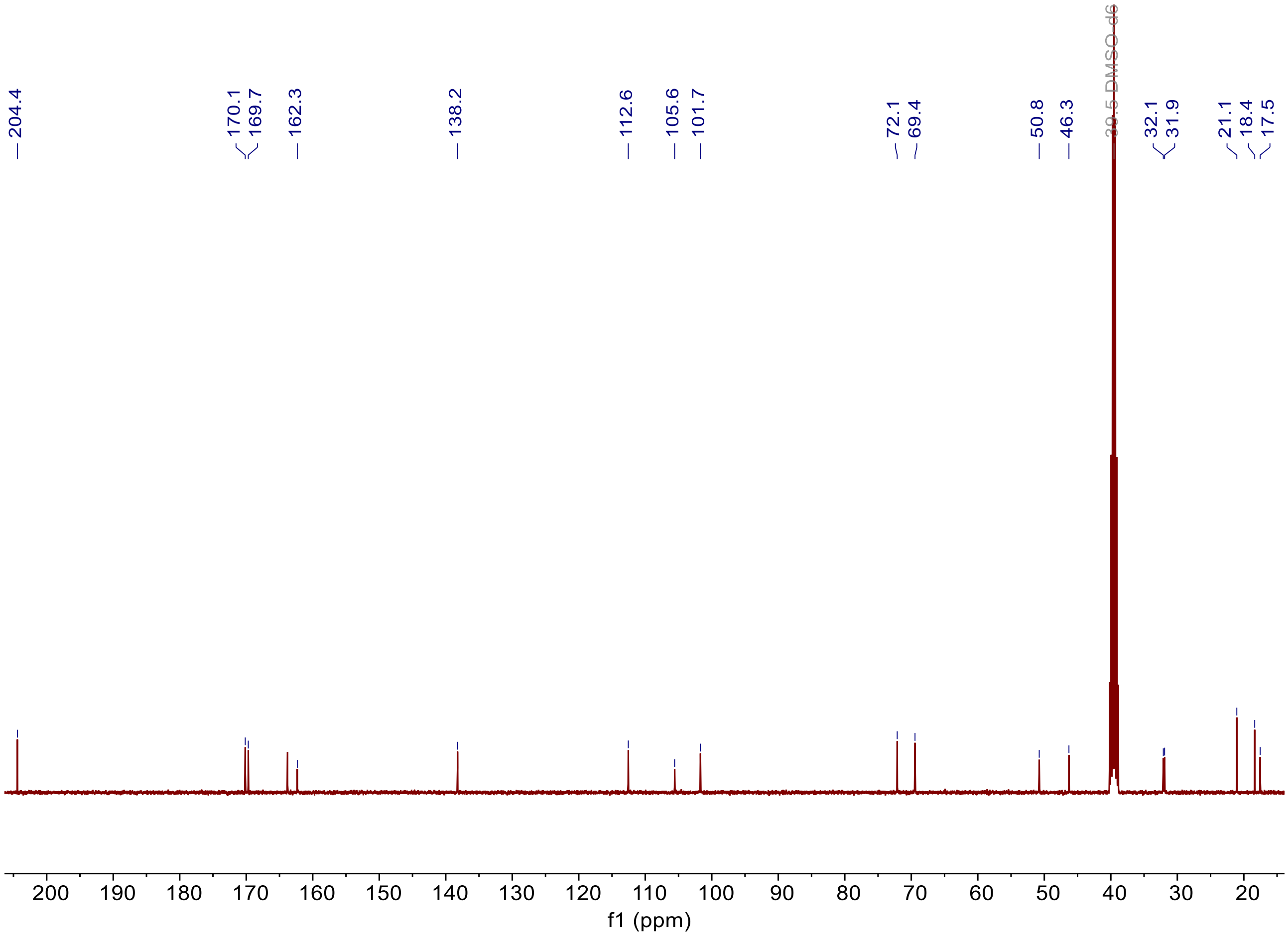

Figure S7. ^{1}H NMR spectrum of **4** in DMSO-d_6 (400 MHz).

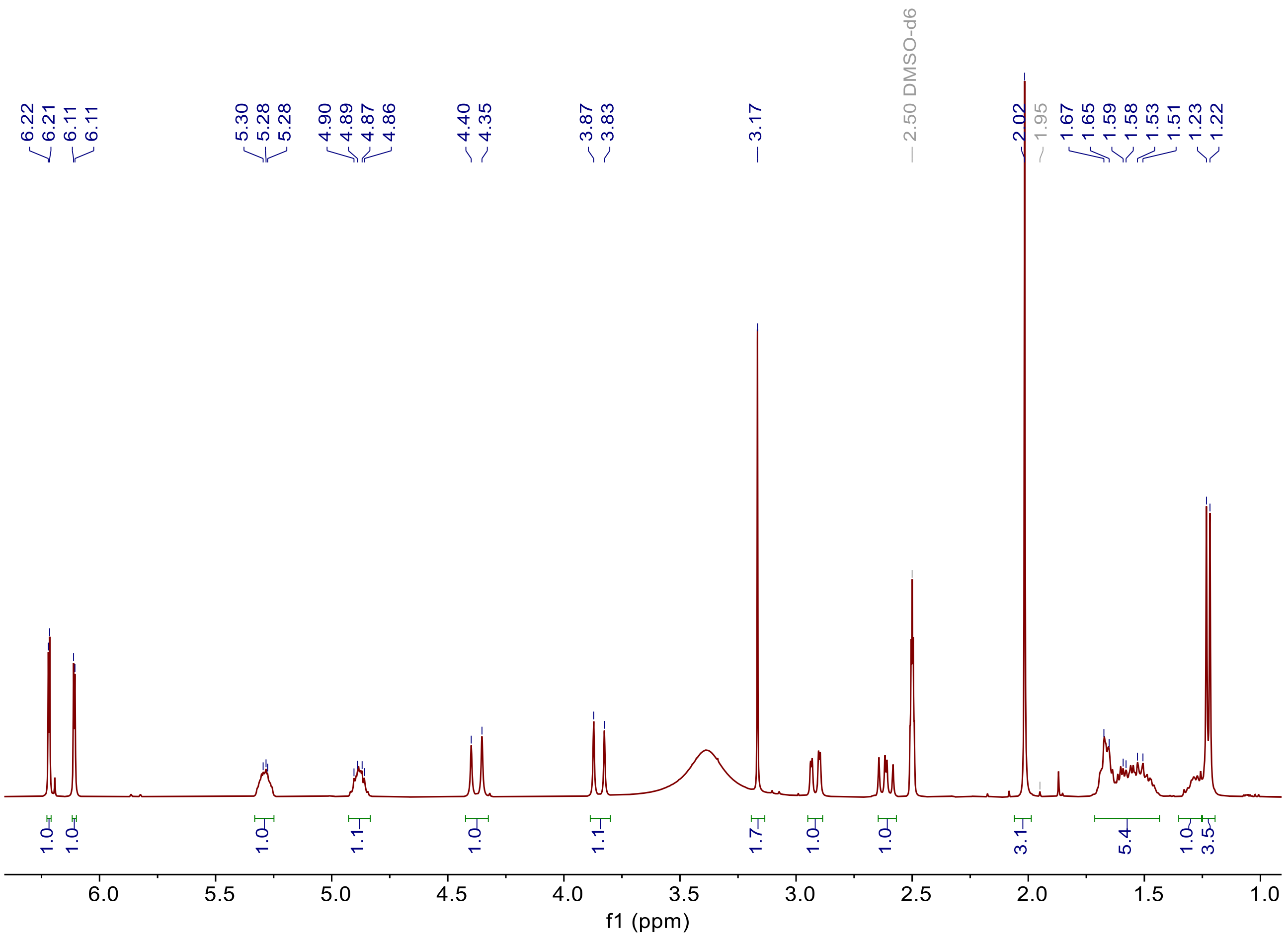

Figure S8. ^{13}C NMR spectrum of **4** in DMSO-d_6 (100 MHz).

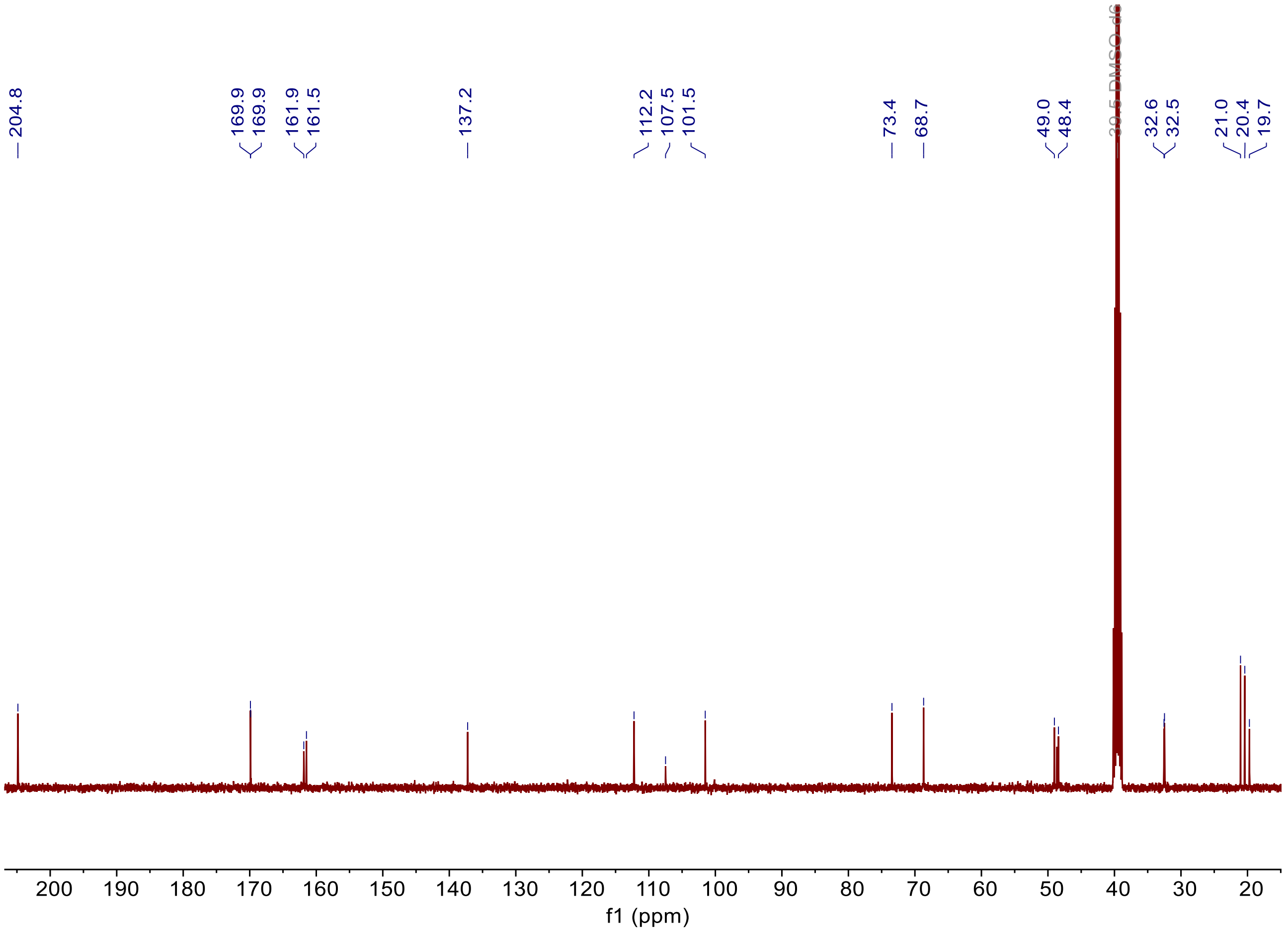

Figure S9. HRESIMS spectrum of compound **4**.

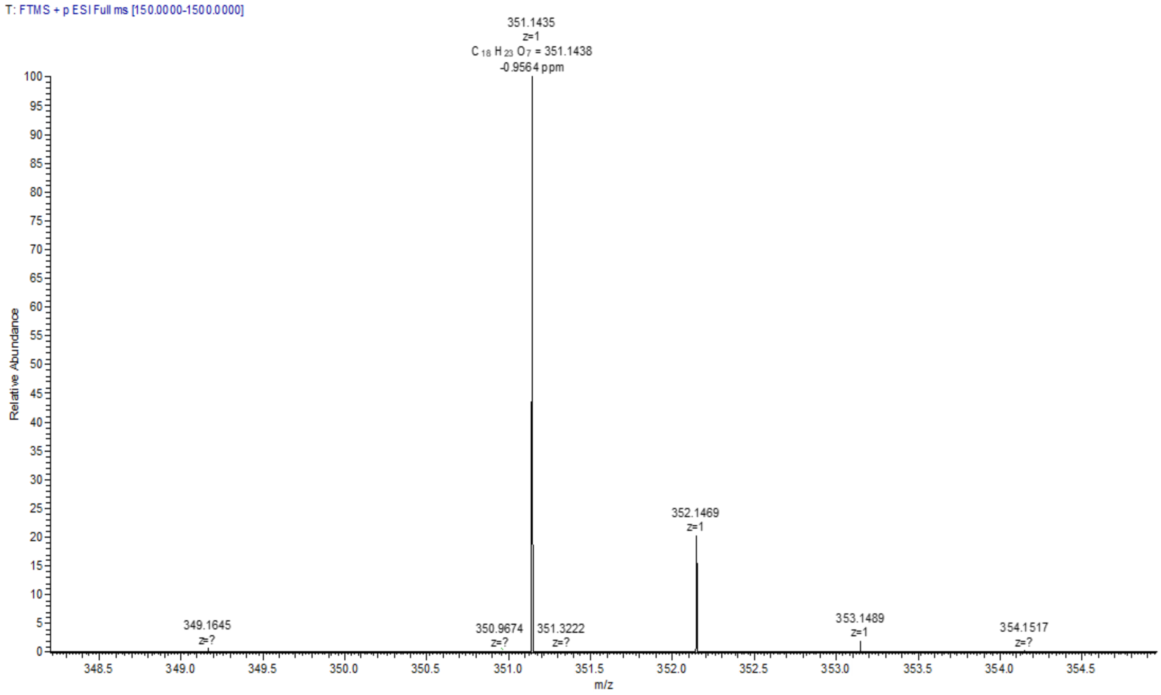

Figure S10. ^{1}H NMR spectrum of **5** in DMSO-d_6 (400 MHz).

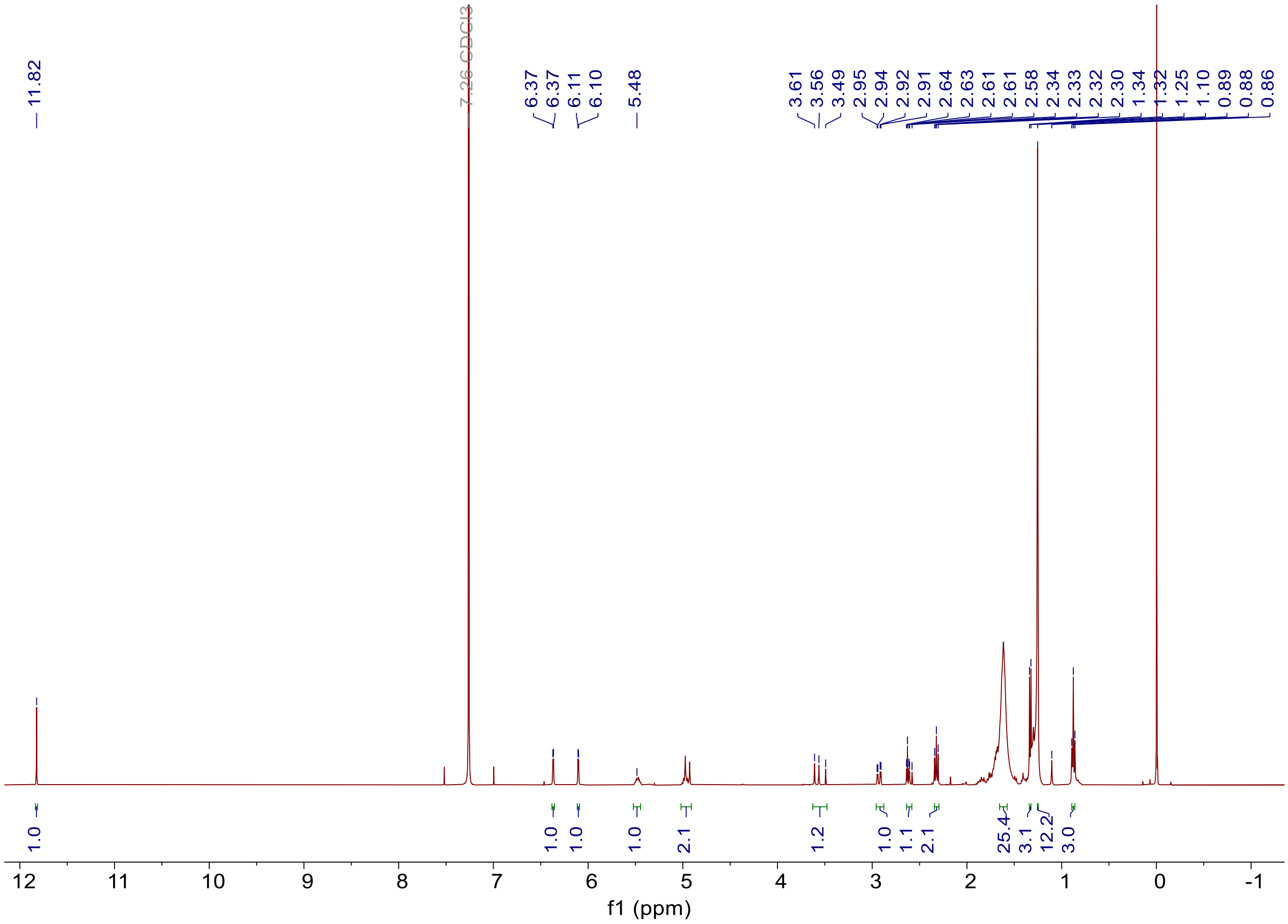

Figure S11. ^{13}C NMR spectrum of **5** in DMSO-d_6 (125 MHz).

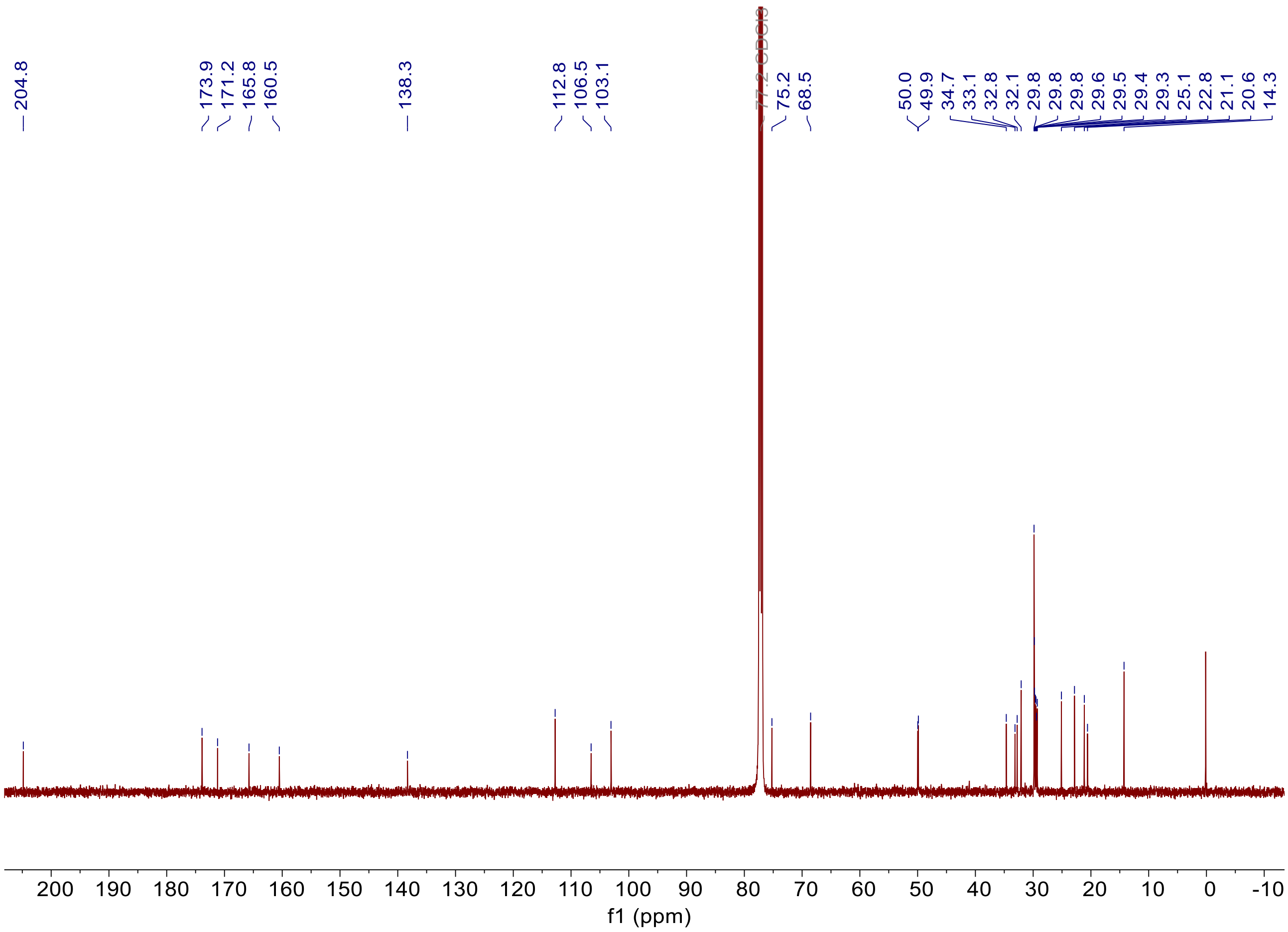

Figure S12. HSQC spectrum of **5** in DMSO-d_6 (500 MHz, 125 MHz).

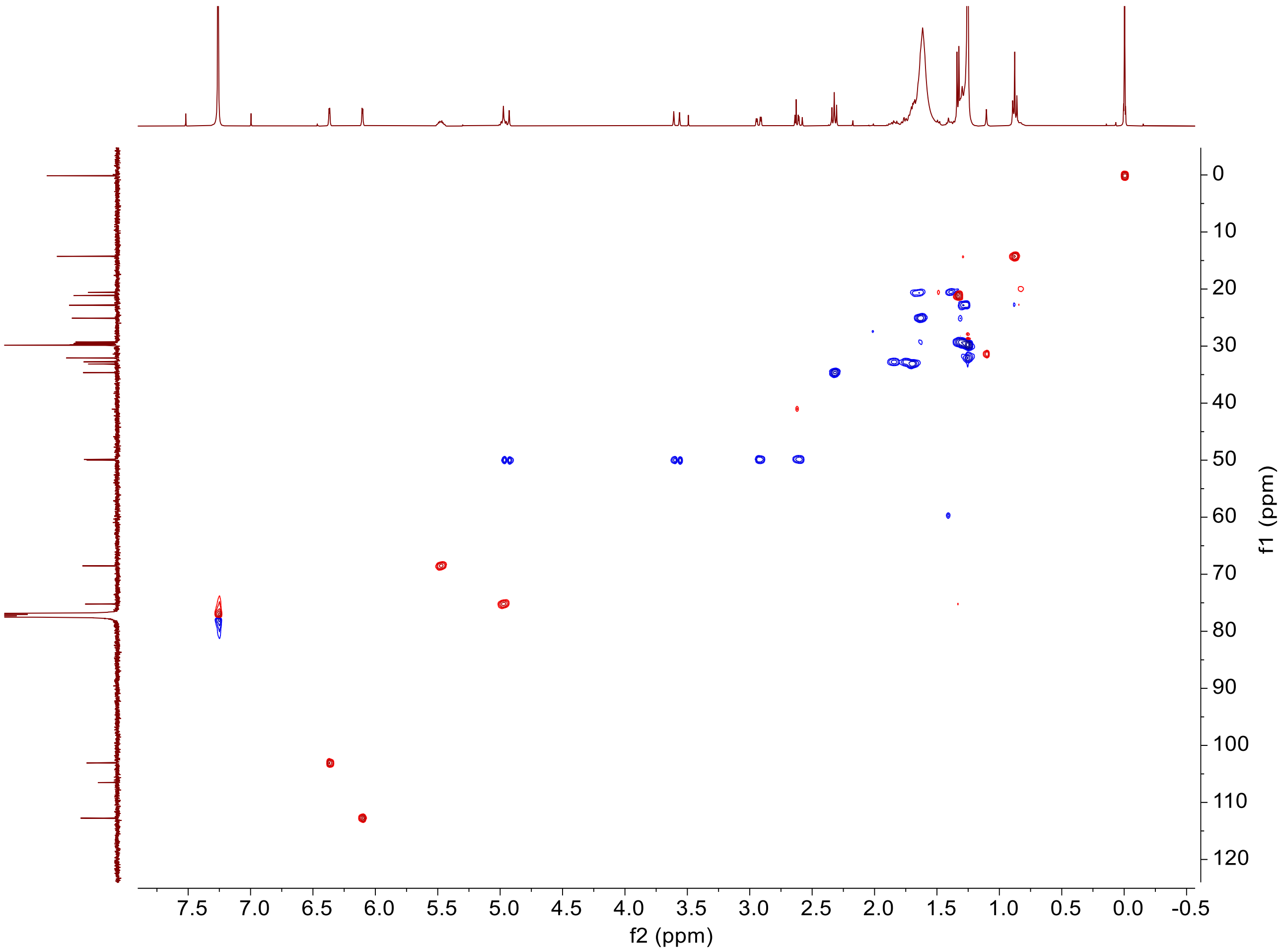

Figure S13. HMBC spectrum of **5** in DMSO-d_6 (500 MHz, 125 MHz).

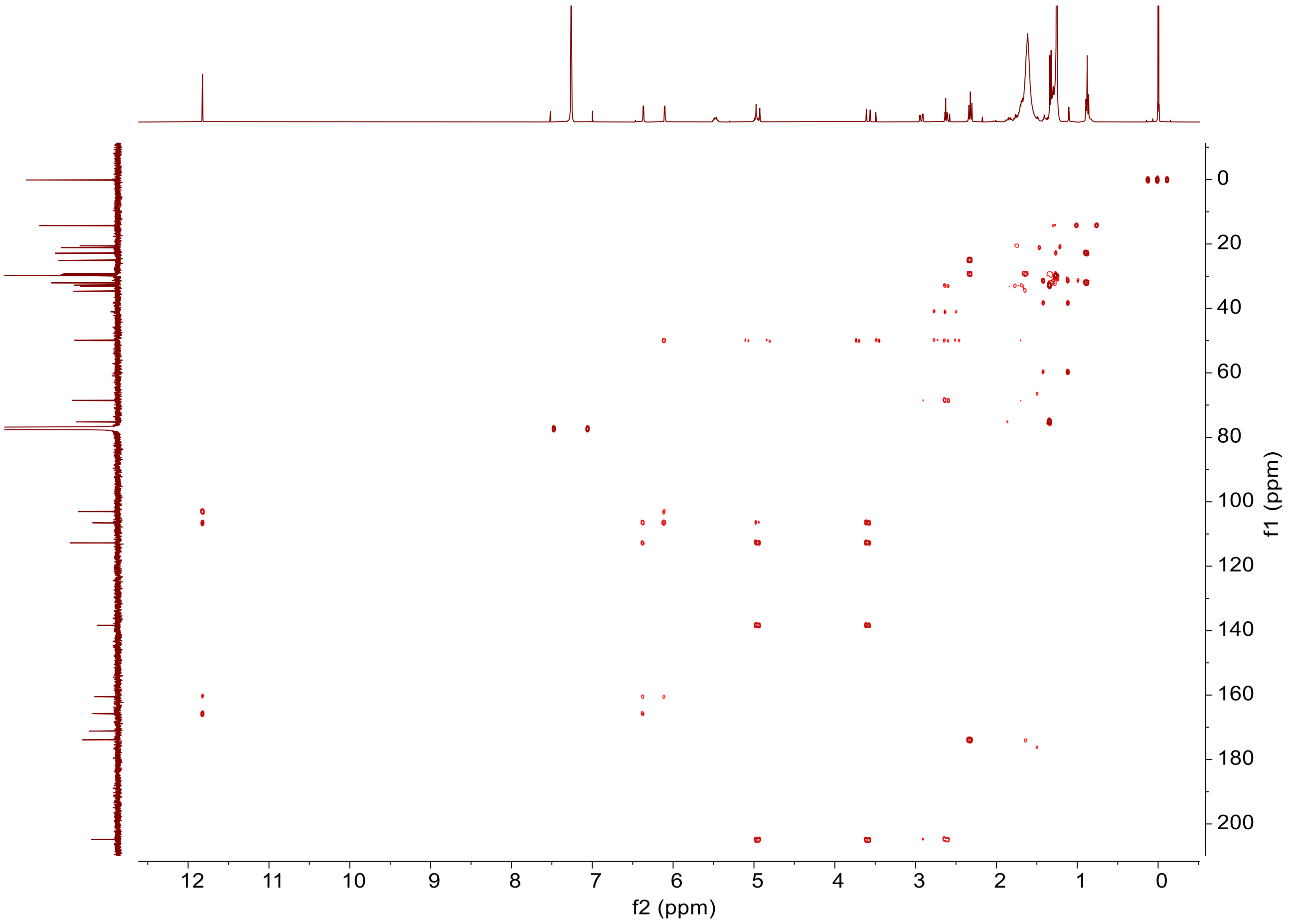

Figure S14. ^{1}H-^{1}H COSY spectrum of **5** in DMSO-d_6 (500 MHz, 500 MHz).

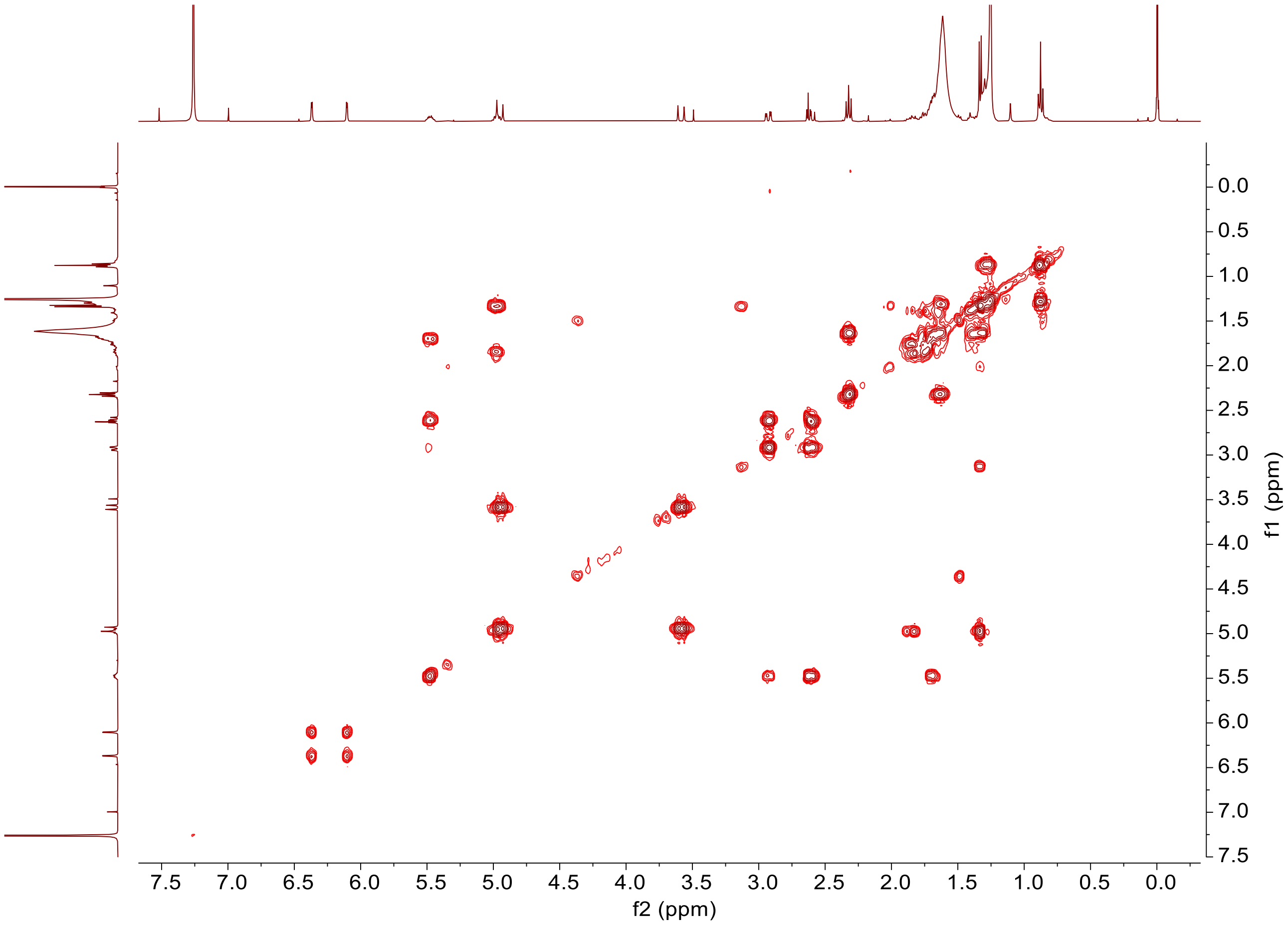

Figure S15. ^{1}H-^{1}H NOESY spectrum of **5** in DMSO-d_6 (500 MHz, 500 MHz).

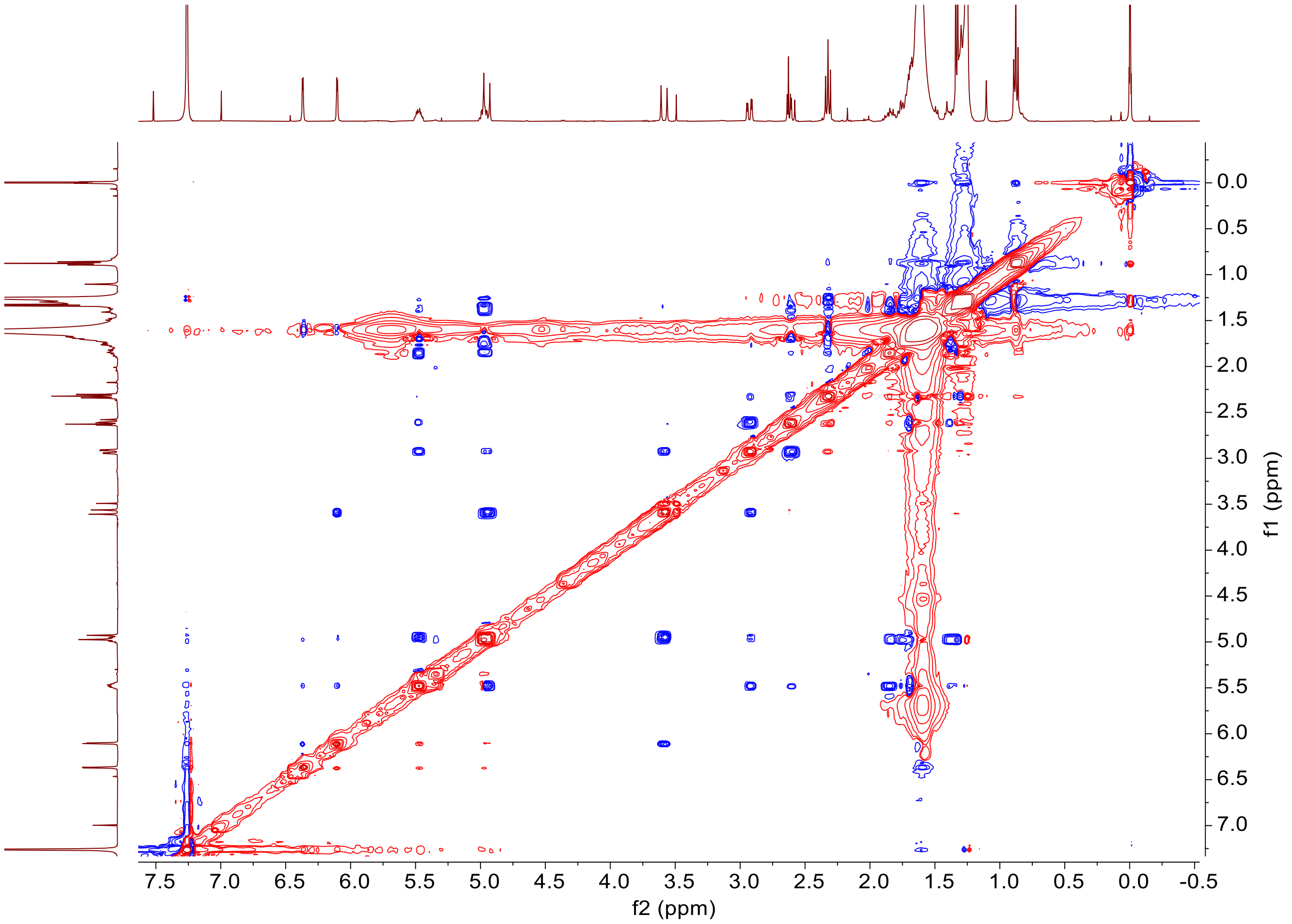

Figure S16. HRESIMS spectrum of compound **5**.

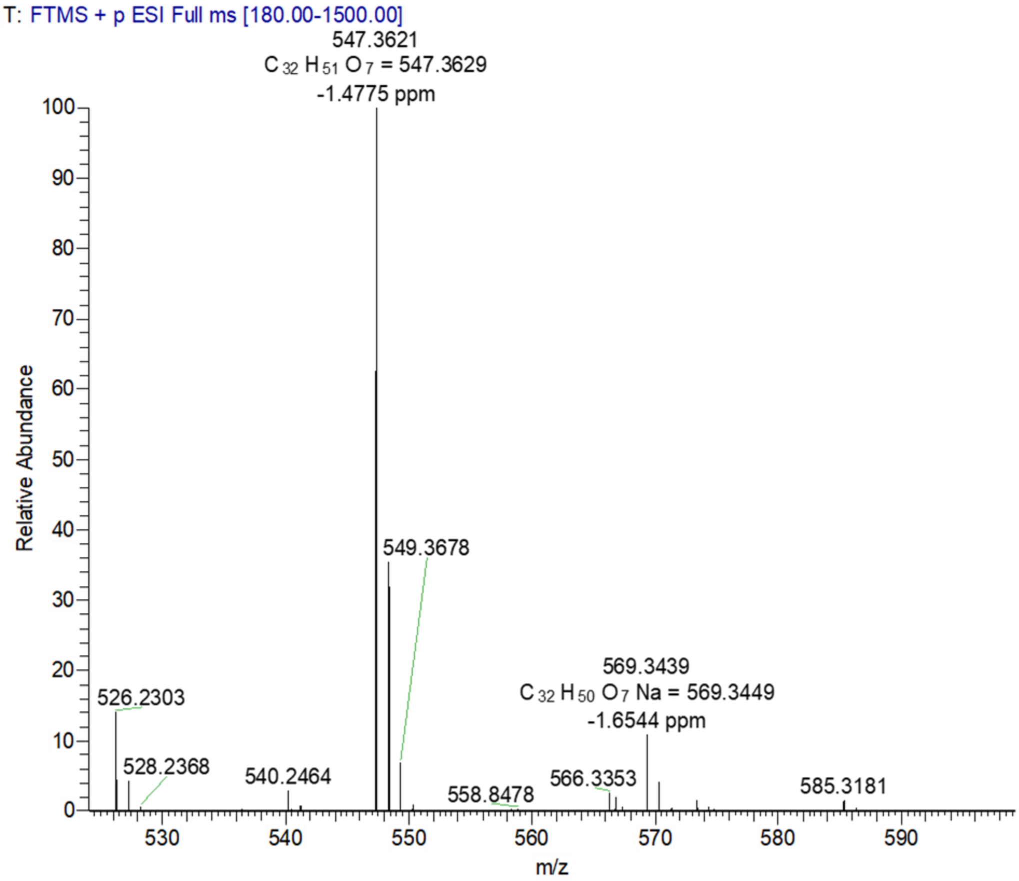

Figure S17. ECD spectrum of compound **5**.

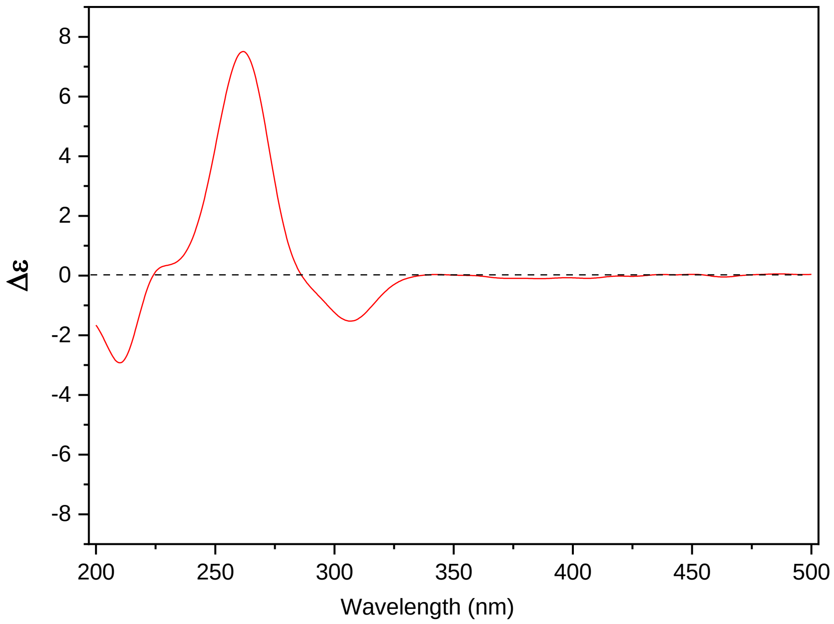

Figure S18. ^{1}H NMR spectrum of **6** in DMSO-d_6 (400 MHz)..

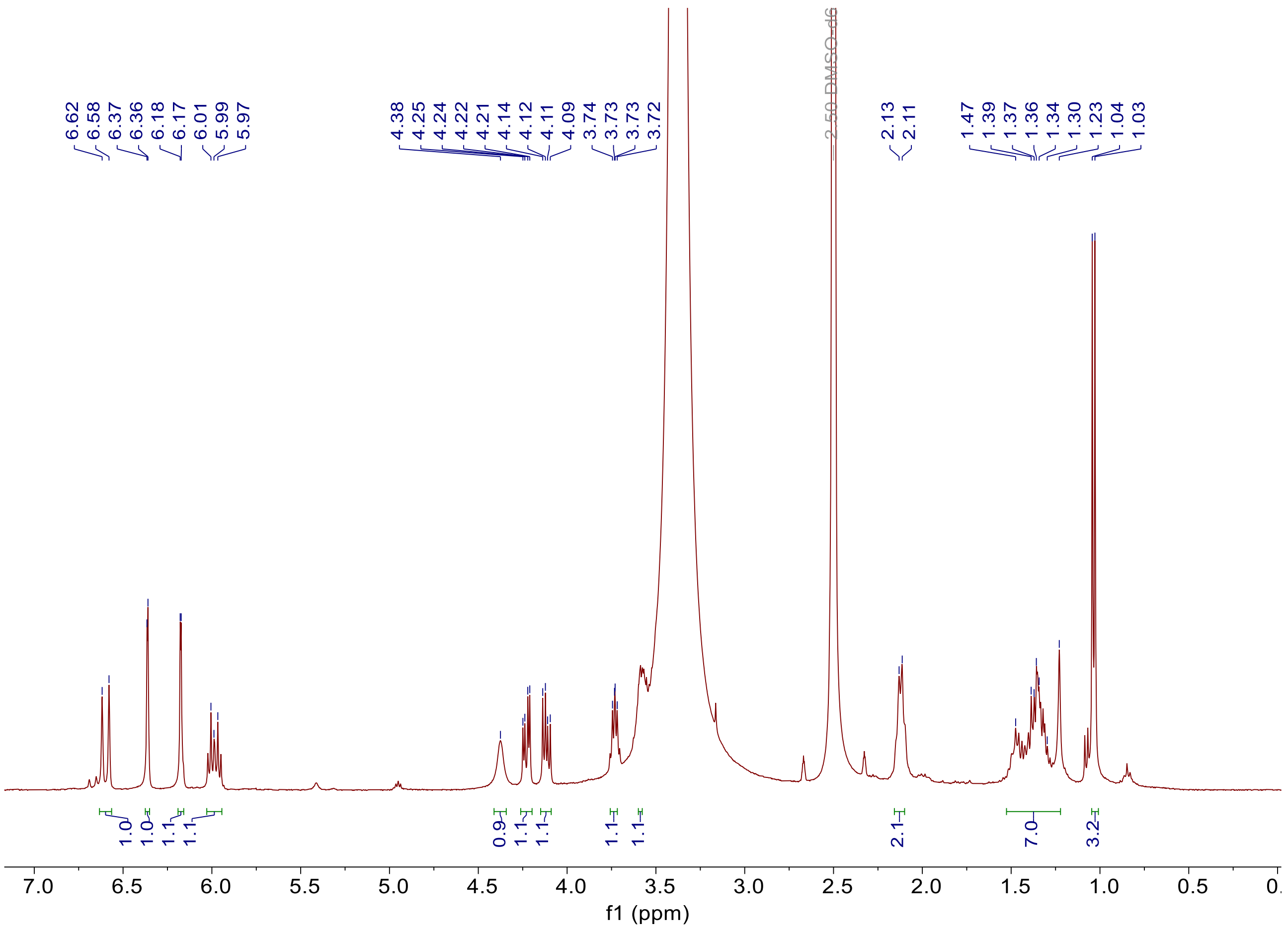

Figure S19. ^{13}C NMR spectrum of **6** in DMSO-d_6 (125 MHz).

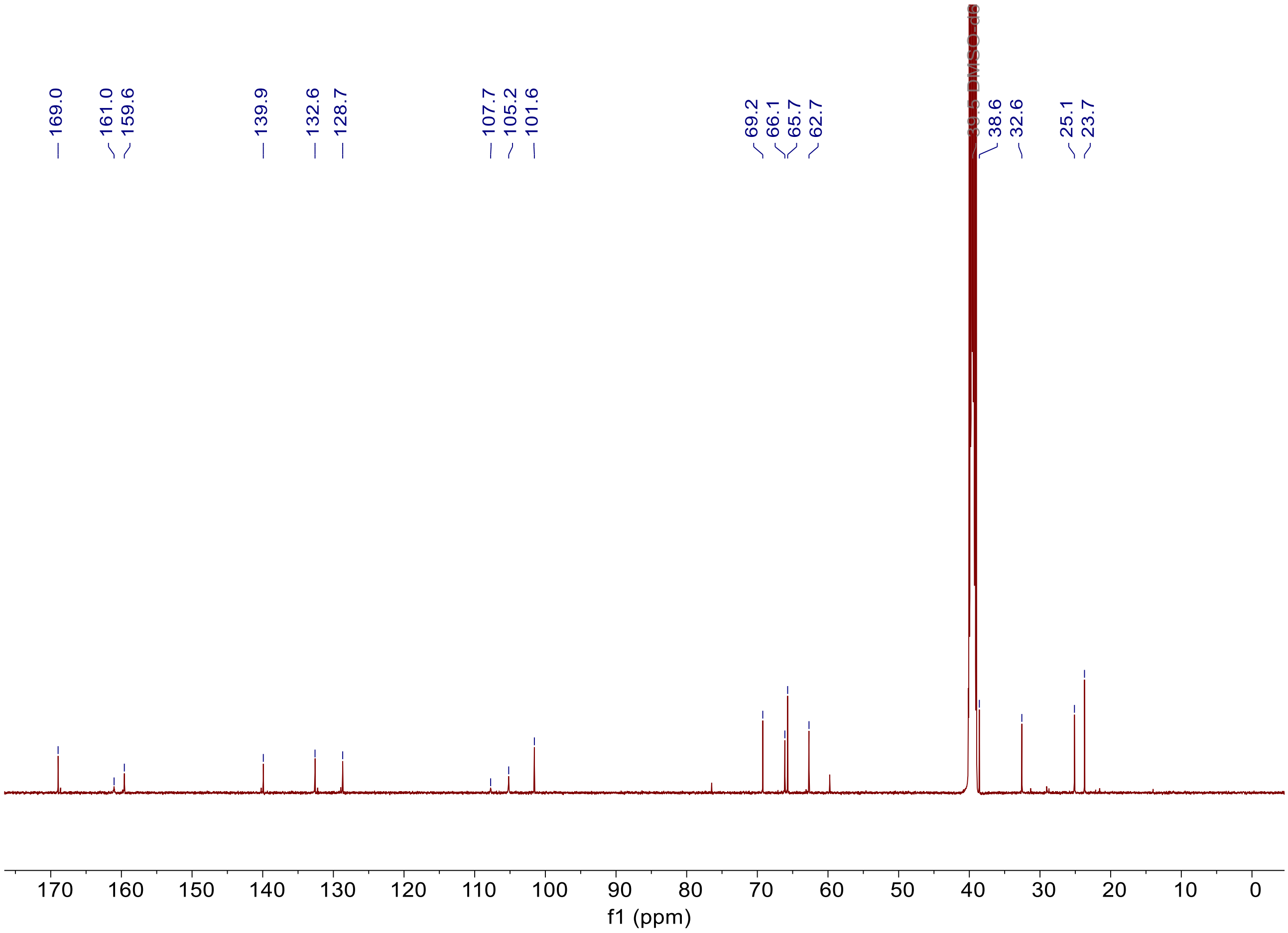

Figure S20. HSQC spectrum of **6** in DMSO-d_6 (500 MHz, 125 MHz).

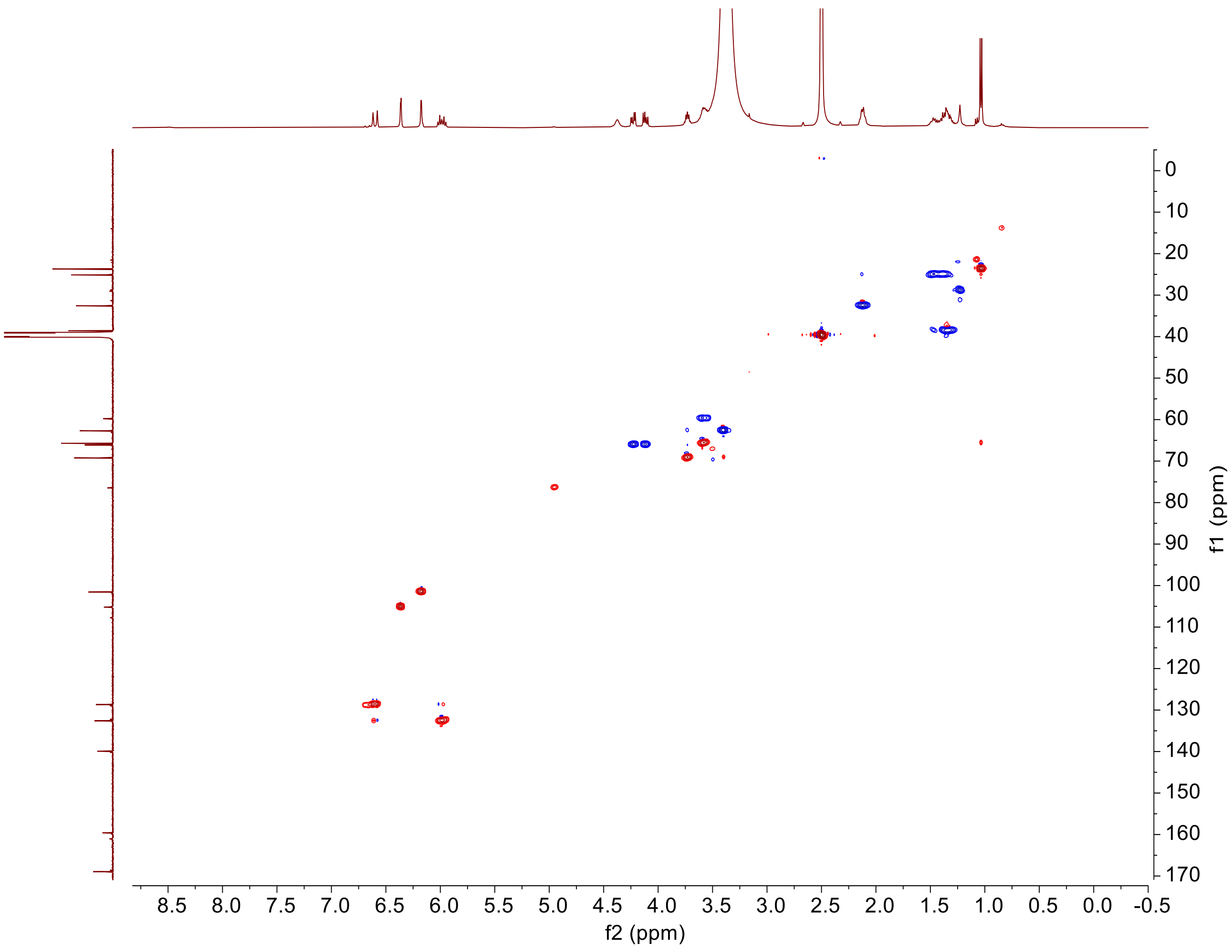

Figure S21. HMBC spectrum of **6** in DMSO-d_6 (500 MHz, 125 MHz).

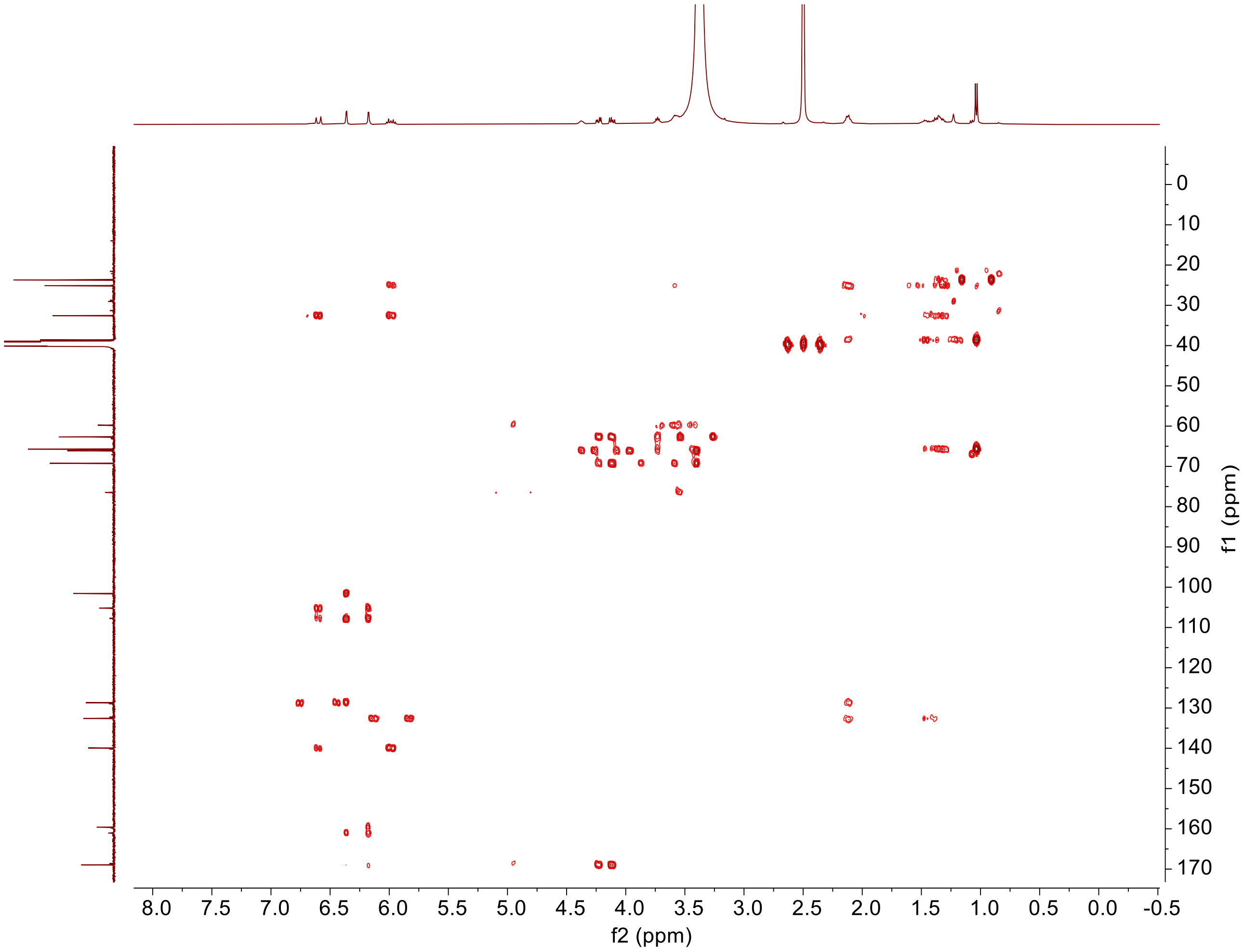

Figure S20. ^{1}H-^{1}H COSY spectrum of **6** in DMSO-d_6 (500 MHz, 500 MHz).

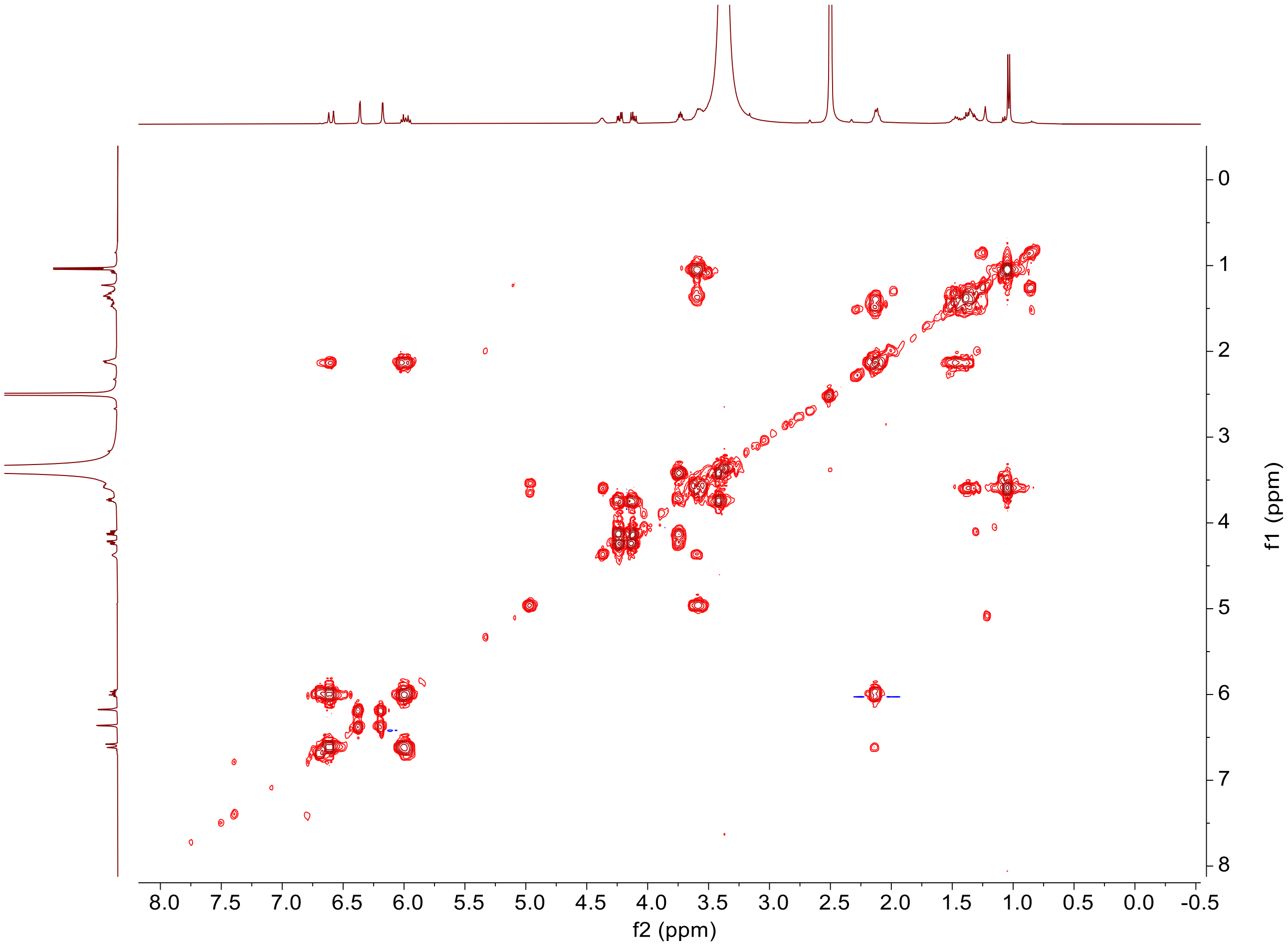

Figure S21. ^{1}H-^{1}H NOESY spectrum of **6** in DMSO-d_6 (500 MHz, 500 MHz).

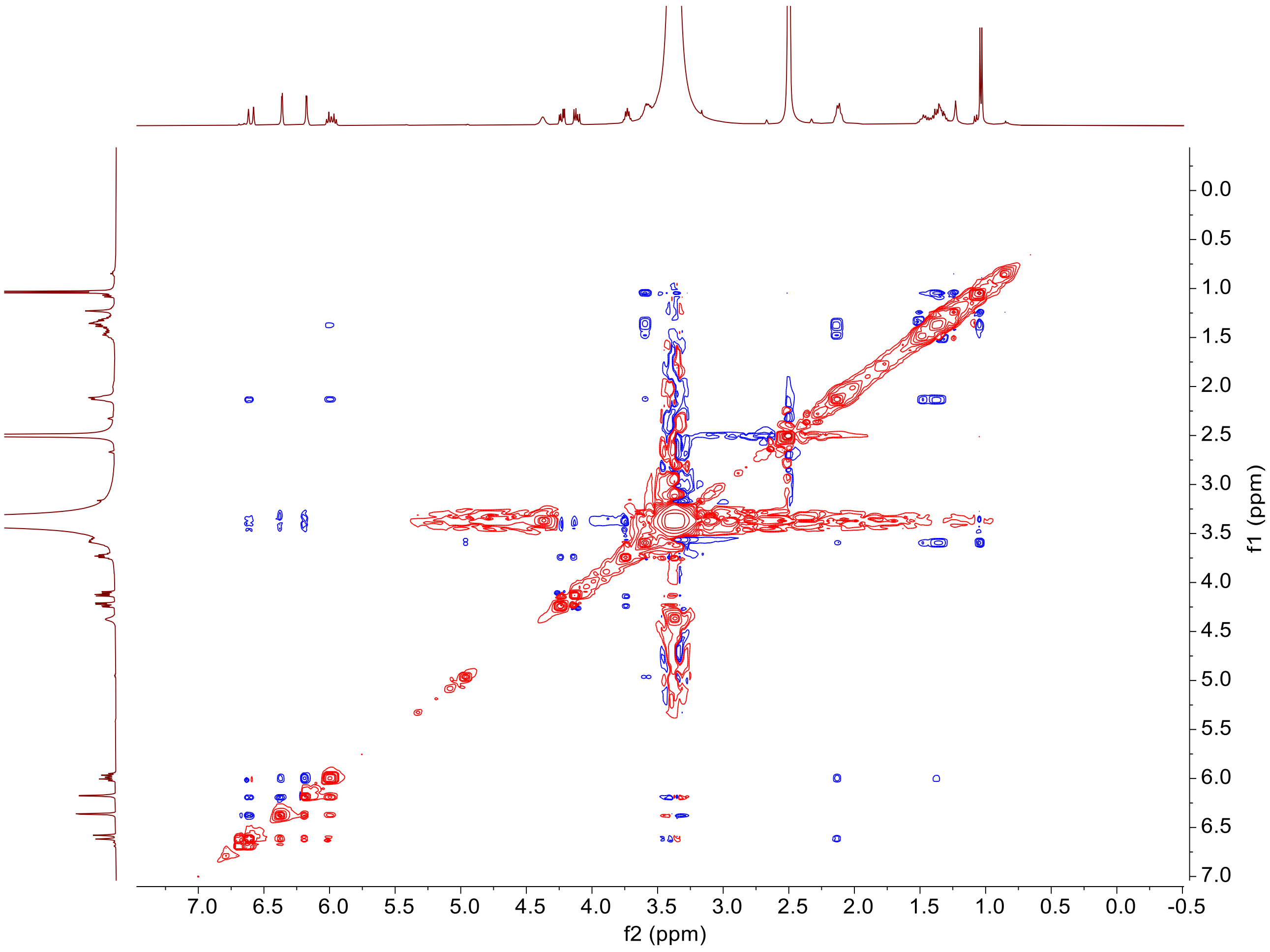

Figure S22. HRESIMS spectrum of compound **6**.

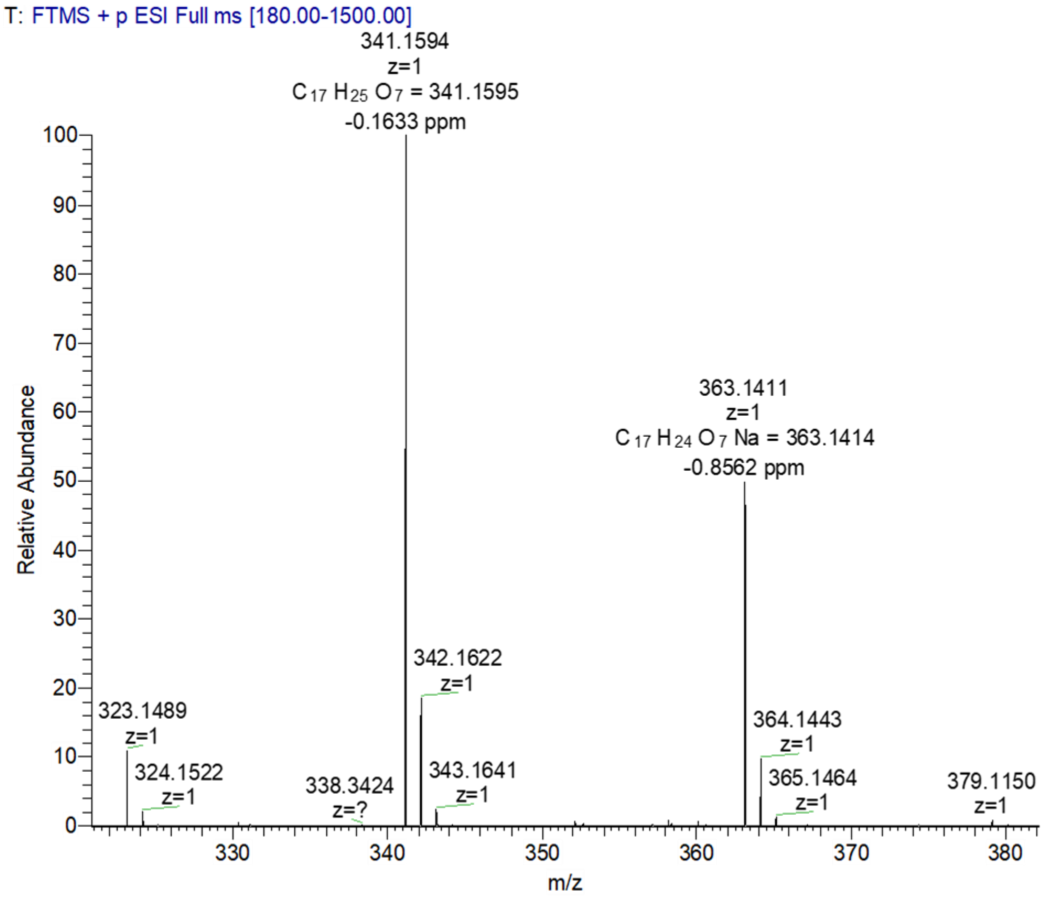

Figure S23. ^{1}H NMR spectrum of **7** in DMSO-d_6 (400 MHz).

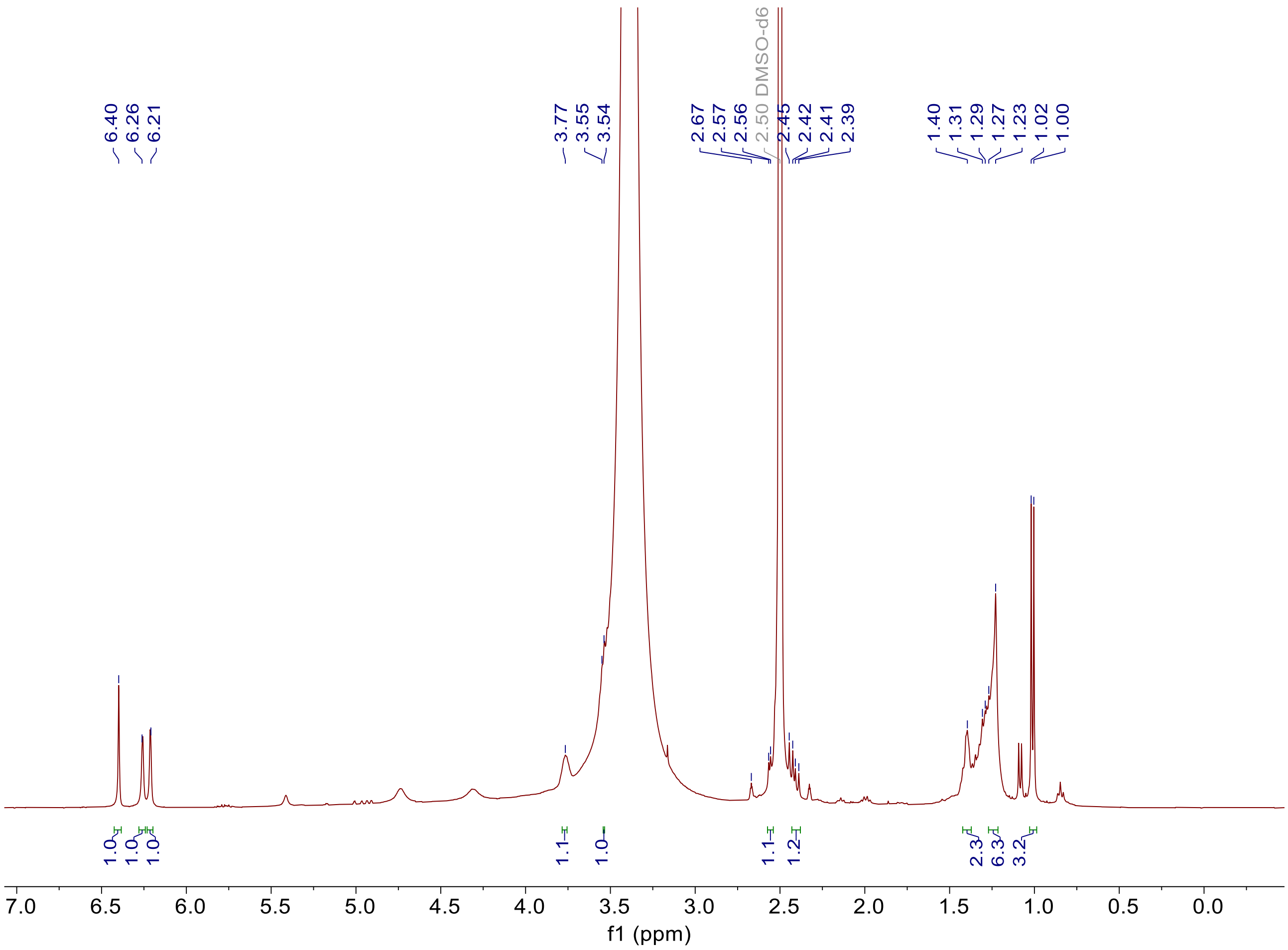

Figure S24. ^{13}C NMR spectrum of **7** in DMSO-d_6 (125 MHz).

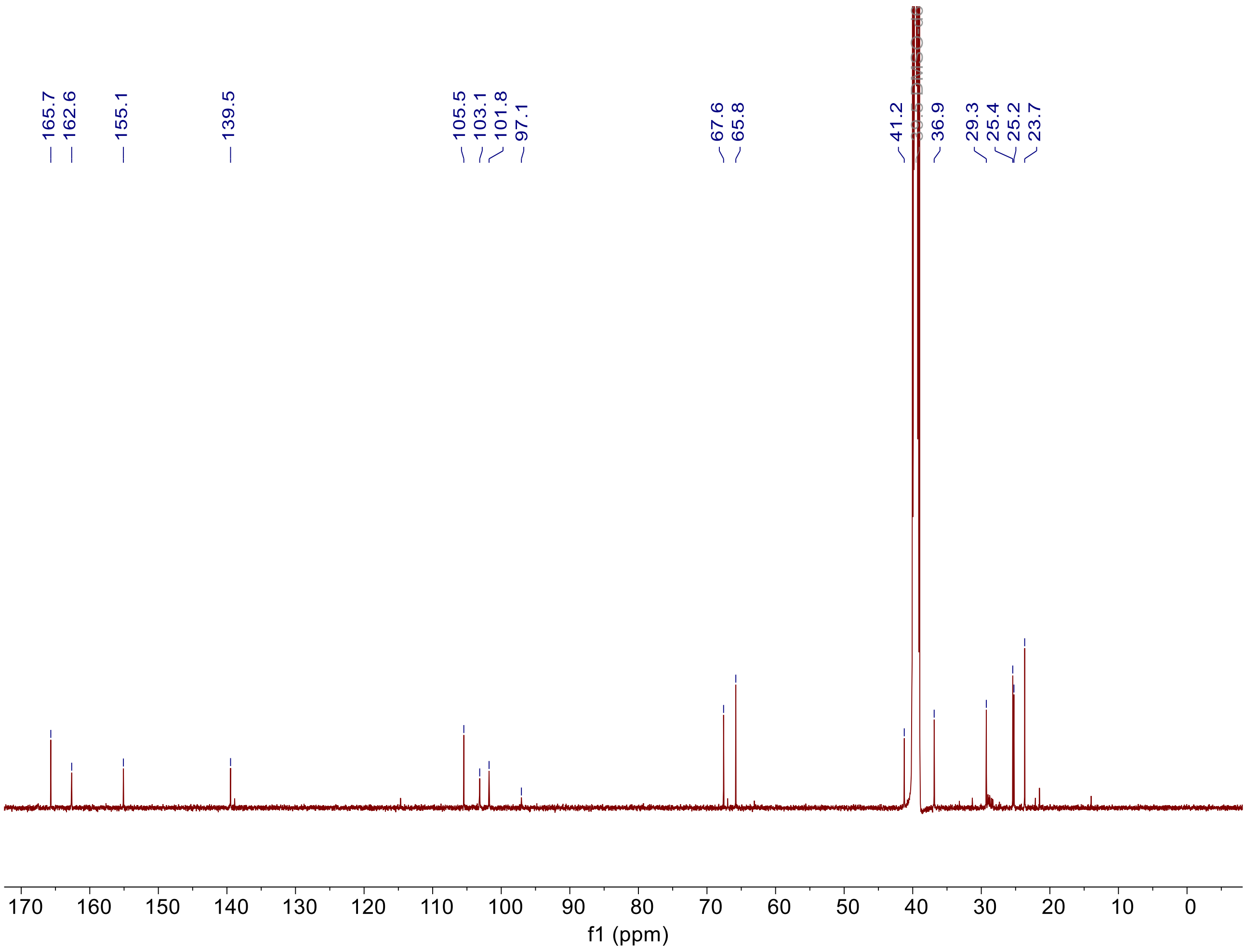

Figure S25. HSQC spectrum of **7** in DMSO-d_6 (500 MHz, 125 MHz).

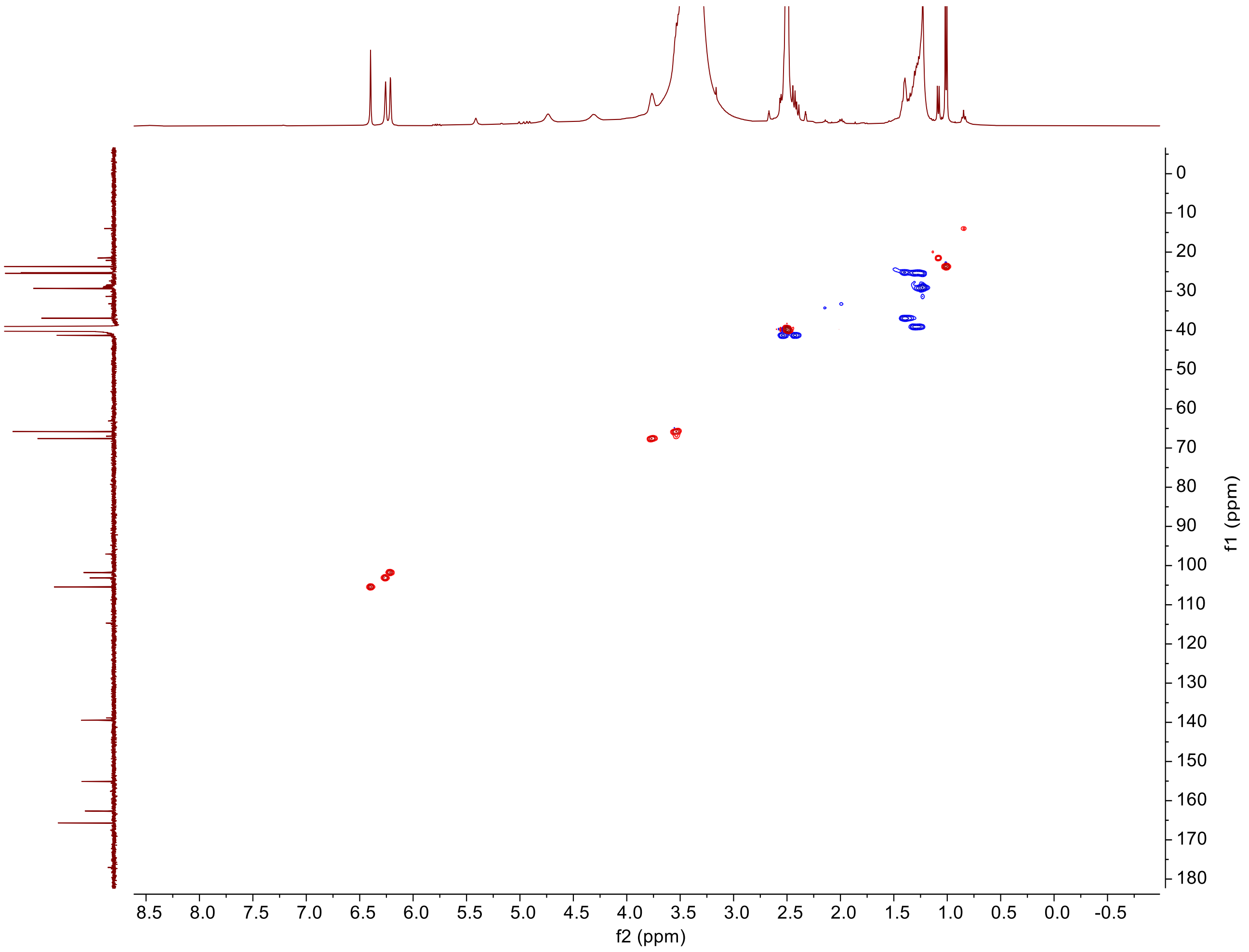

Figure S26. HMBC spectrum of **7** in DMSO-d_6 (500 MHz, 125 MHz).

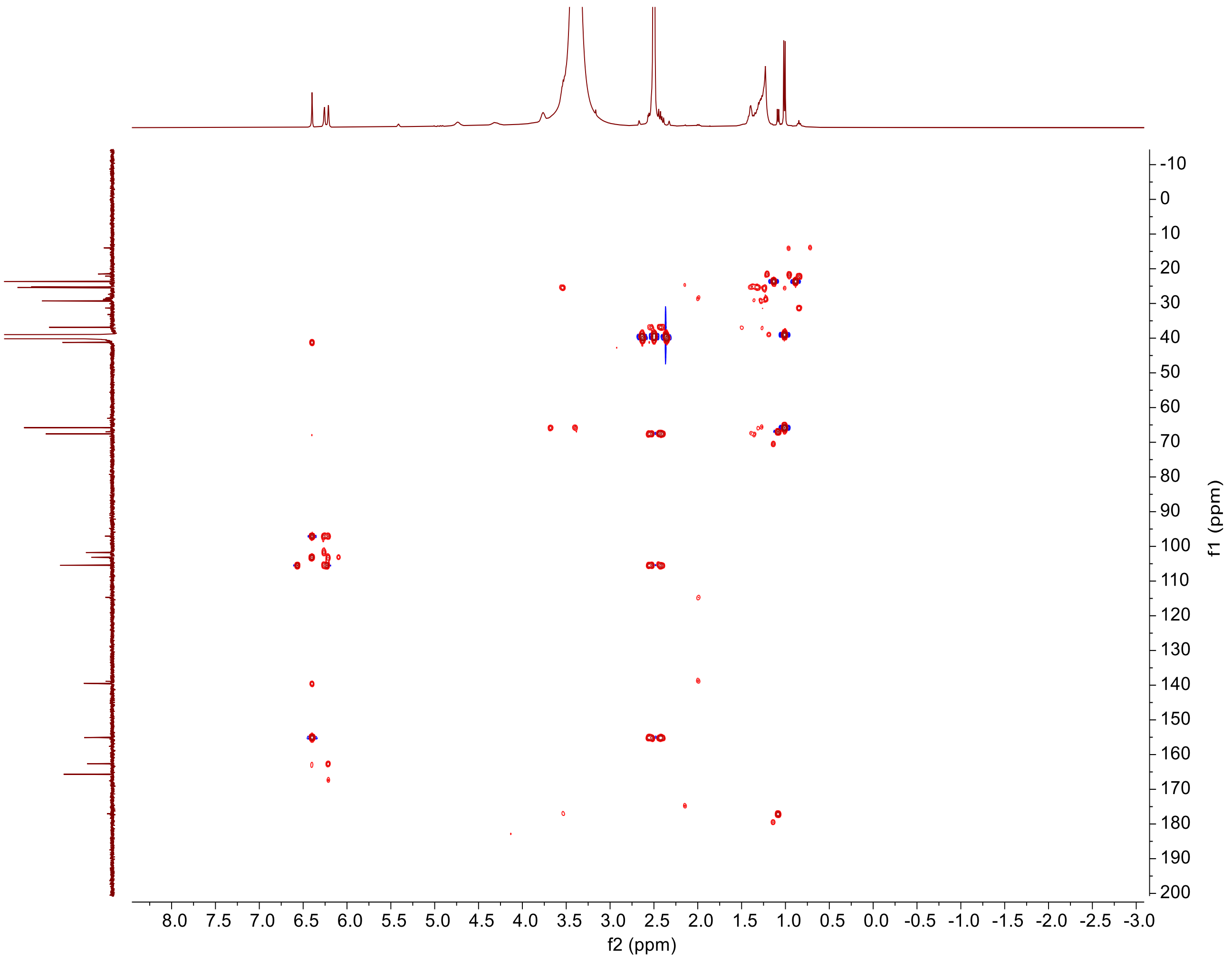

Figure S27. ^{1}H-^{1}H COSY spectrum of **7** in DMSO-d_6 (500 MHz, 500 MHz).

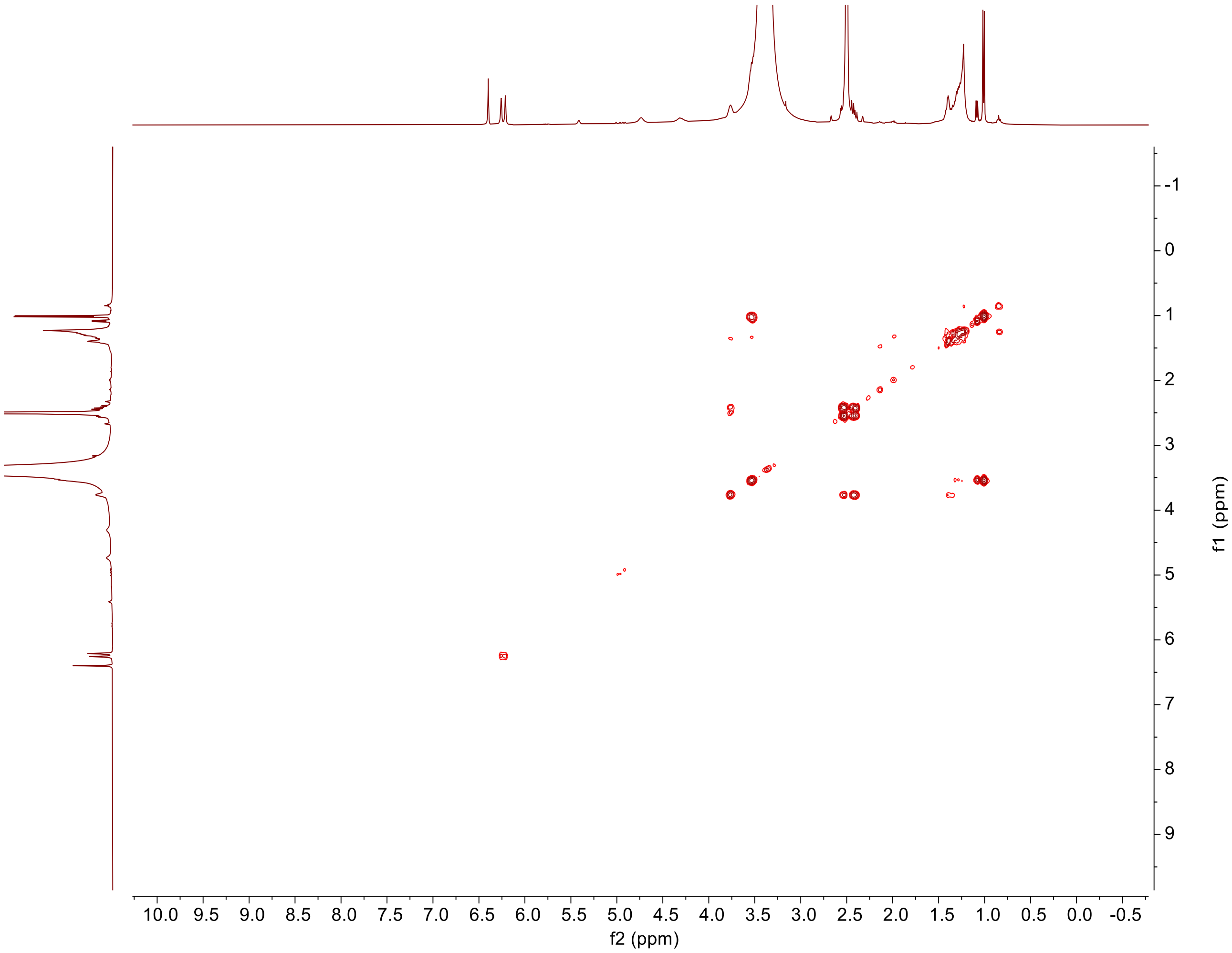

Figure S28. HRESIMS spectrum of compound **7**.

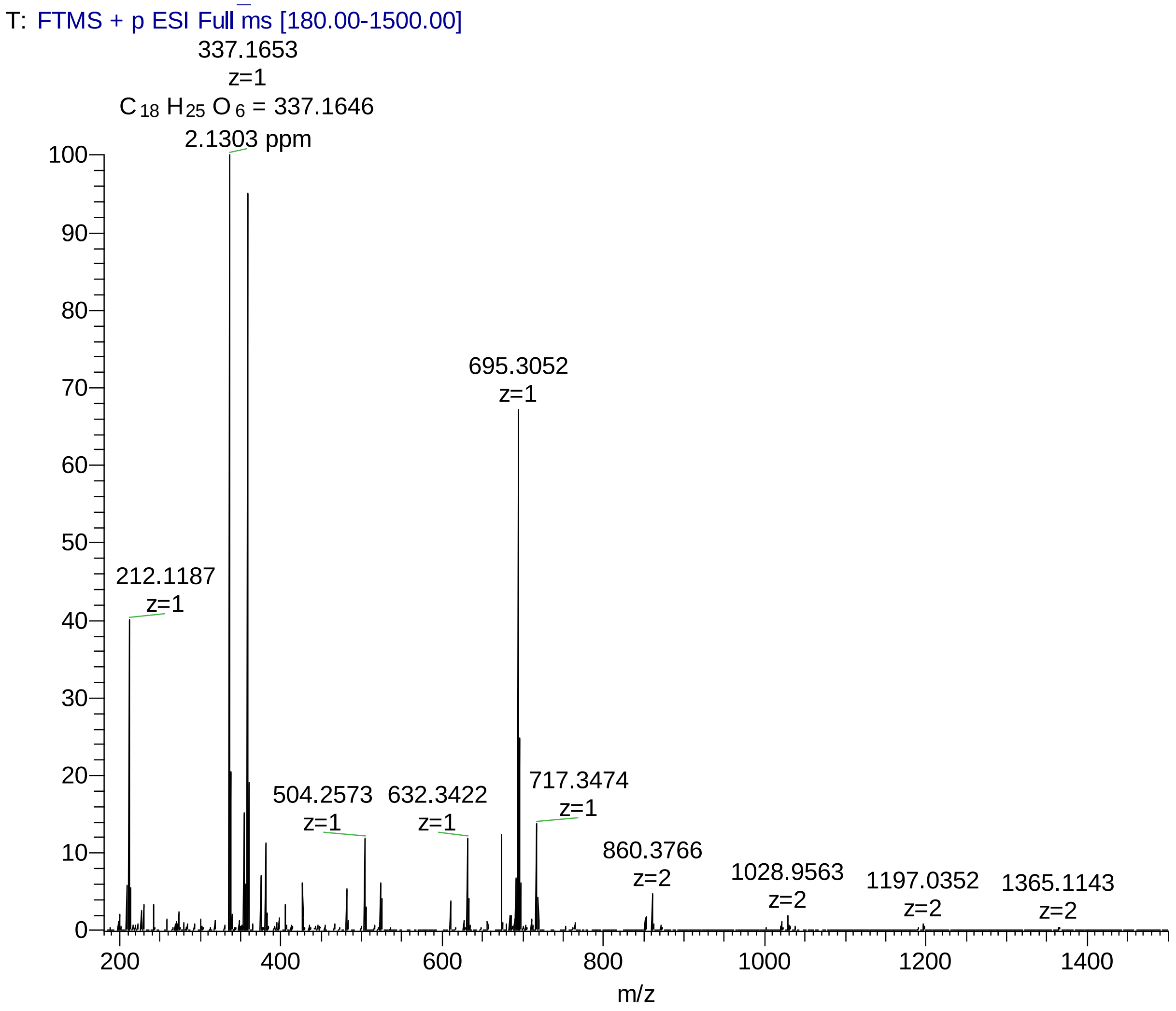

Figure S29. ^{1}H NMR spectrum of **8** in DMSO-d_6 (400 MHz).

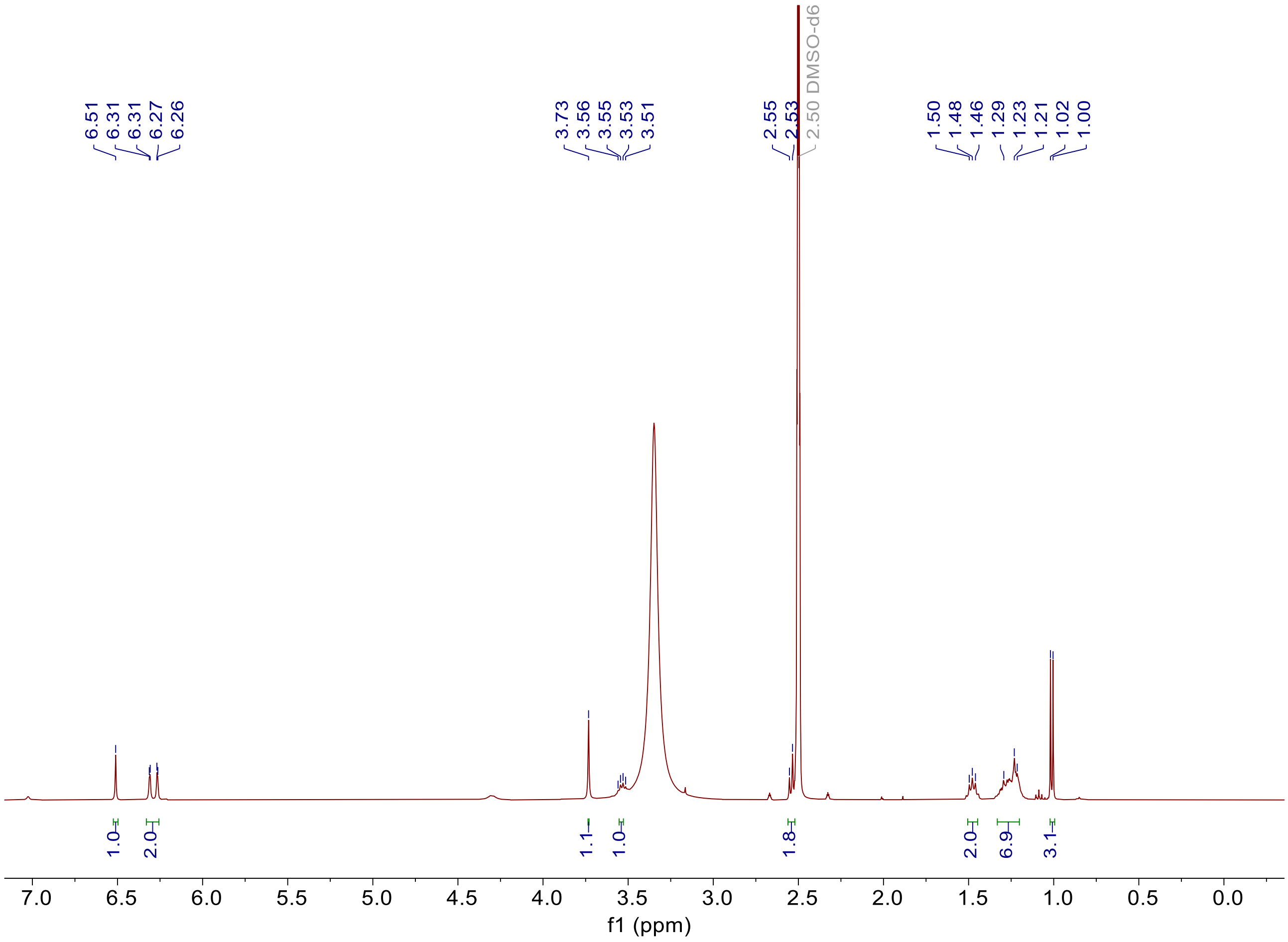

Figure S30. ^{13}C NMR spectrum of **8** in DMSO-d_6 (125 MHz).

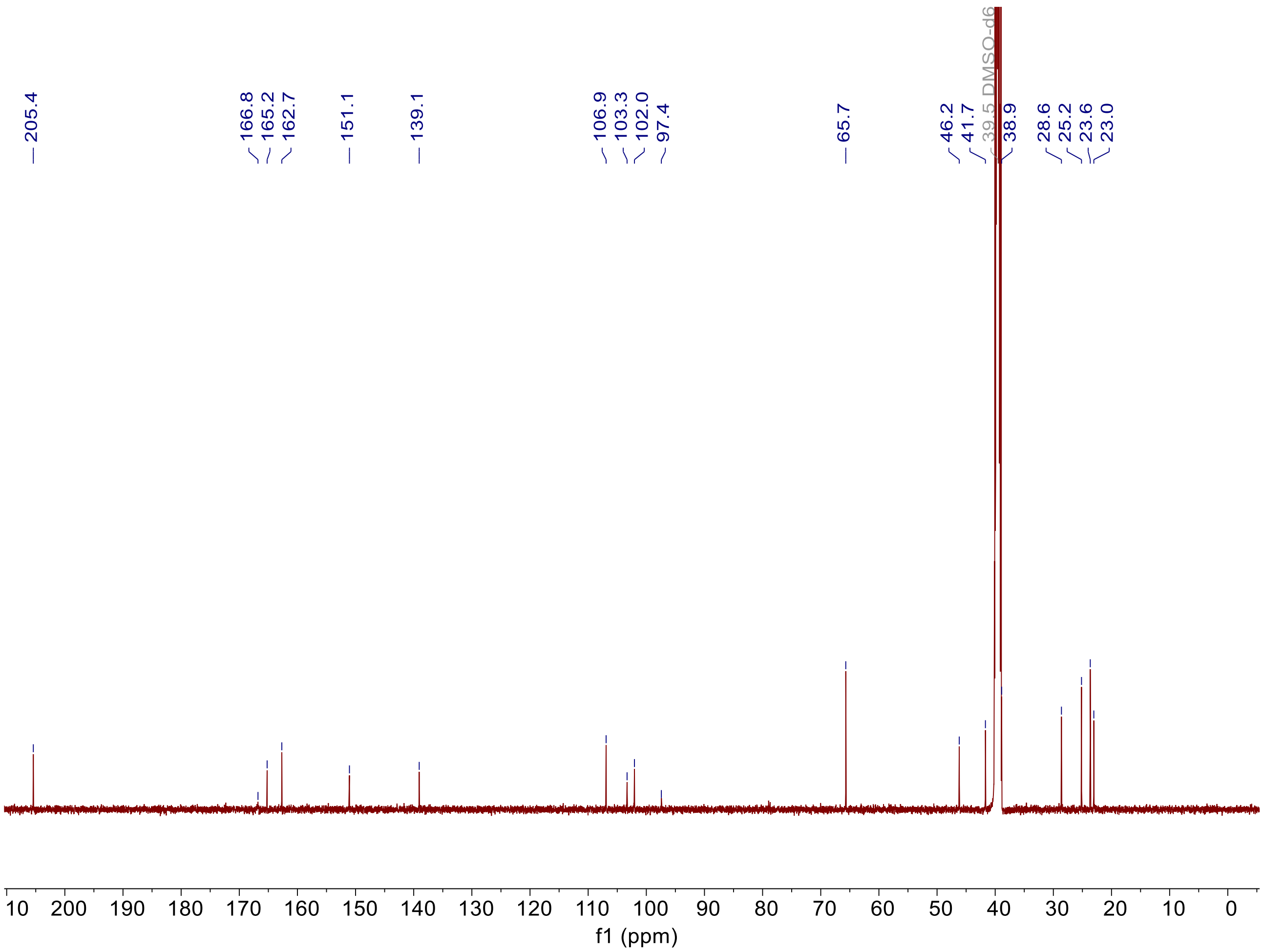

Figure S31. ^{1}H NMR spectrum of **9** in DMSO-d_6 (400 MHz)..

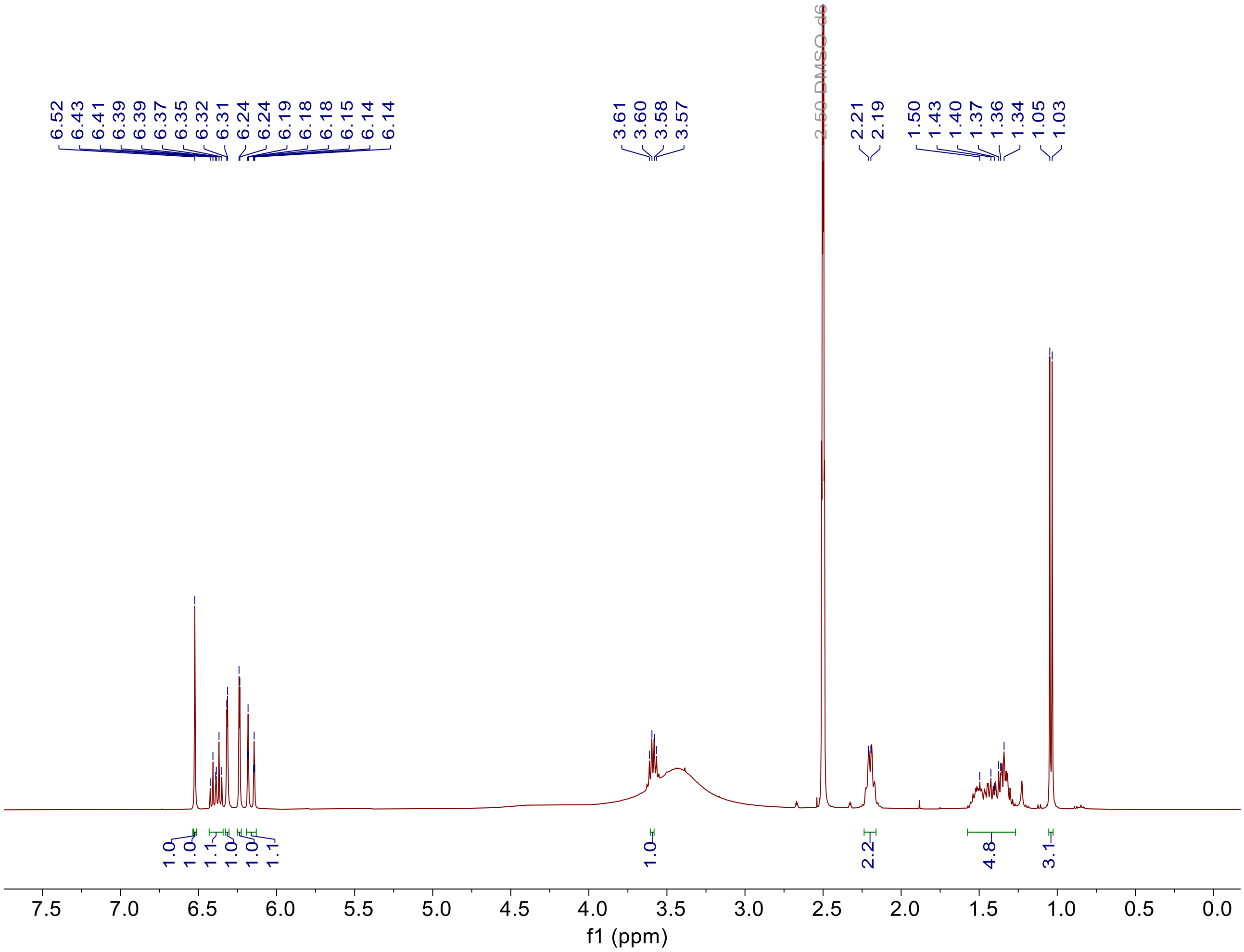

Figure S32. ^{13}C NMR spectrum of **9** in DMSO-d_6 (100 MHz).

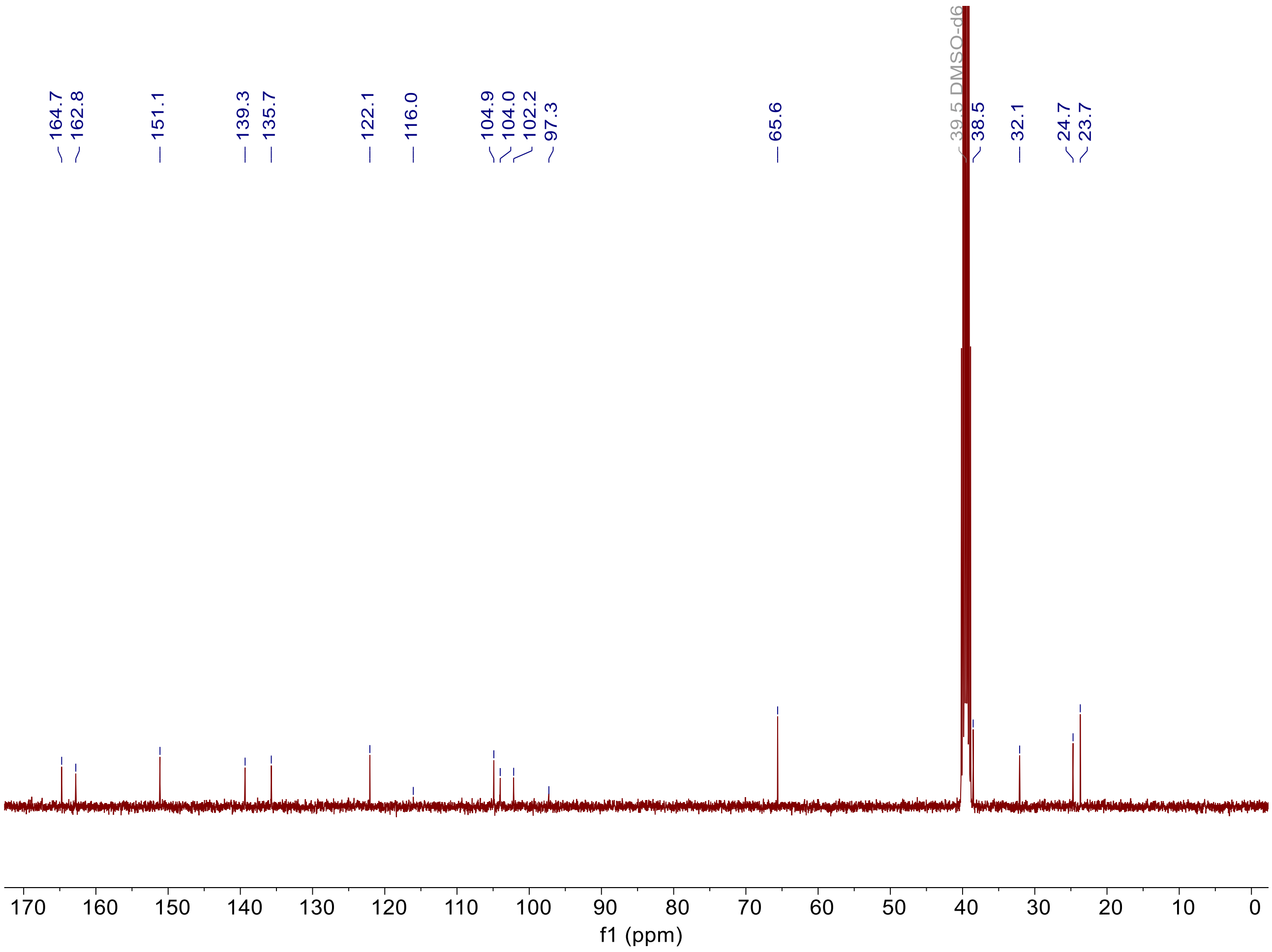

Figure S33. ^{1}H NMR spectrum of **10** in DMSO-d_6 (400 MHz)..

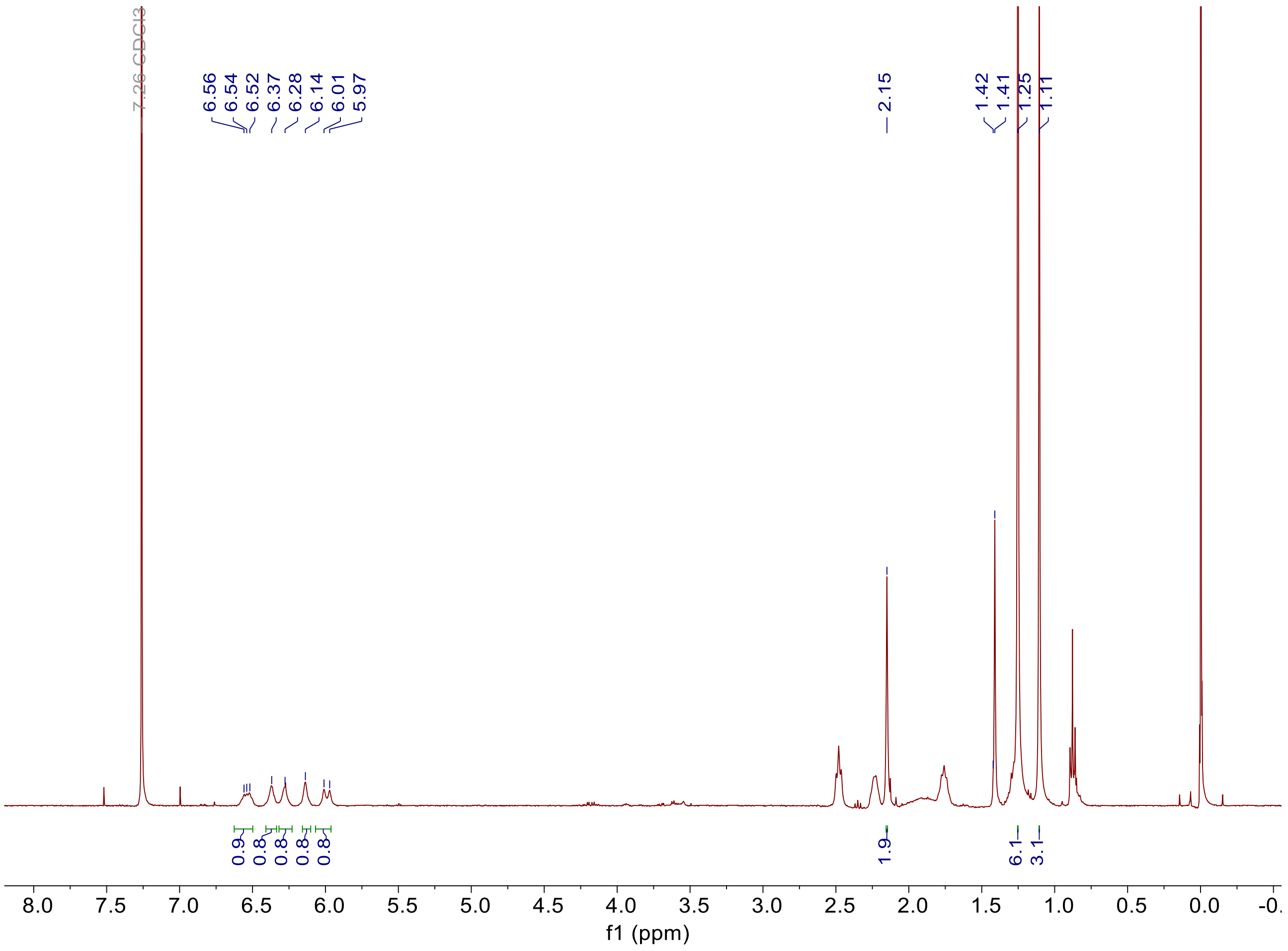

Figure S34. ^{13}C NMR spectrum of **10** in DMSO-d_6 (125 MHz).

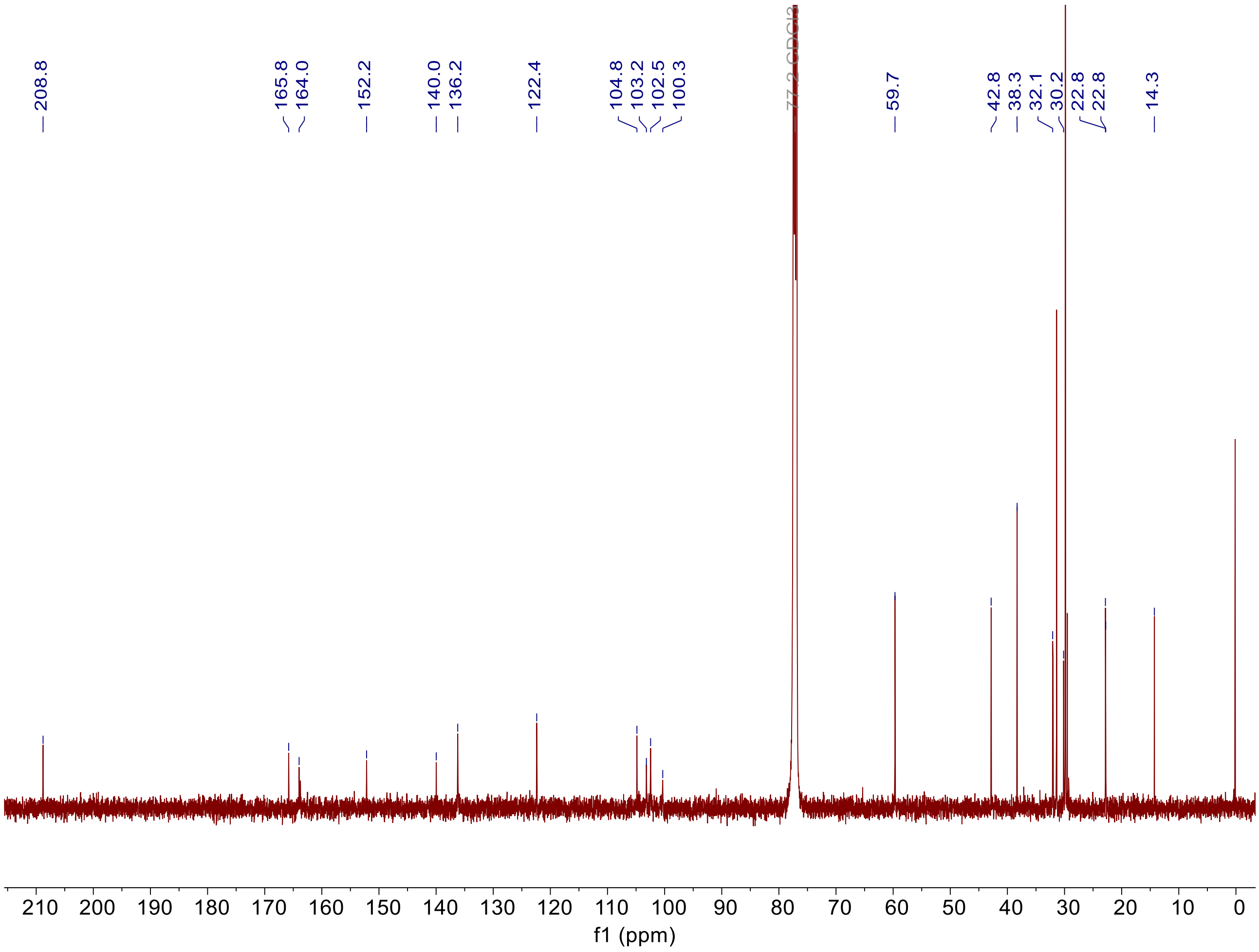

Table S1. ^1H (400 MHz) and ^{13}C (100 MHz) NMR data for compound **1** in DMSO-d_6.

No.	1			
	δ_C, type	δ_C, type^a	δ_H, mult (J in Hz)	δ_H, mult (J in Hz)^a
1	170.9, C	172.5, C		
2	104.9, C	105.9, C		
3	164.5, C	166.8, C		
4	101.6, CH	102.9, CH	6.22, d (2.4)	6.25, d (2.5)
5	162.5, C	163.6, C		
6	112.8, CH	113.8, CH	6.16, d (2.3)	6.15, d (2.5)
7	137.8, C	139.6, C		
8	49.9, CH_2	51.4, CH_2	4.38, d (18.4)	4.56, d (18.4)
9	201.6, C	204.4, C		
10	132.1, CH	132.4, CH	6.48, d (12.0)	6.49, dt (11.8, 1.4)
11	139.0, CH	139.9, CH	5.74, ddd (11.9, 9.6, 5.9)	5.80, ddd (12.0, 10.0, 5.9)
12	26.1, CH_2	27.4, CH_2	α 2.14, m;<br>β 2.35, m	α 2.22, m;<br>β 2.50, m
13	24.7, CH_2	26.1, CH_2	1.58, m	1.66, m
14	30.6, CH_2	32.0, CH_2	α 1.58, m;<br>β 1.72, m	α 1.66, m;<br>β 1.84, m
15	75.1, CH	76.5, CH	4.92, m	5.02, bp (6.5)
15-CH_3	20.7, CH_3	21.4, CH_3	1.21, d (6.1)	1.34, d (6.3)

^{a}The literature data [25] were recorded in $CDCl_3$; the solvent difference should be considered when comparing the chemical shifts.

Table S2. ^{1}H (400 MHz) and ^{13}C (100 MHz) NMR data for compound **2** in DMSO-d_6.

No.	**2**			
	δ_C, type	δ_C, type^a	δ_H, mult (J in Hz)	δ_H, mult (J in Hz)^a
1	171.1, C	172.6, C		
2	106.6, C	106.8, C		
3	165.8, C	166.7, C		
4	103.1, CH	103.0, CH	6.30, d (2.5)	6.25, d (2.1)
5	160.6, C	163.9, C		
6	112.7, CH	113.9, CH	6.05, d (2.5)	6.10, d (2.1)
7	138.6, C	140.4, C		
8	51.0, CH_2	52.0, CH_2	α 3.68, d (18.4); β 4.66, d (18.4)	α 3.77, d (18.5); β 4.67, d (18.5)
9	209.2, C	211.7, C		
10	41.9, CH_2	42.9, CH_2	α 2.60, ddd (15.0,9.8,1.9); β 2.38, ddd (15.1,9.5,2.1)	α 2.65, m; β 2.33, m
11	21.4, CH_2	22.2, CH_2	1.81, m	1.80, m
12	27.1, CH_2	28.3, CH_2	1.49, m	1.47, m
13	21.2, CH_2	22.3, CH_2	1.45, m	1.47, m
14	31.8, CH_2	32.9, CH_2	1.54, m	1.63, m
15	73.7, CH	74.7, CH	5.15, m	5.11, m
15-CH_3	19.3, CH_3	19.6, CH_3	1.31, d (6.3)	1.28, d (6.2)

^{a}The literature data [26] were recorded in CD_3OD-d_4; the solvent difference should be considered when comparing the chemical shifts.

Table S3. ^{1}H (400 MHz) and ^{13}C (100 MHz) NMR data for compound **3** in DMSO-d_6.

No.	**3**			
	δ_C, type	δ_C, type[a]	δ_H, mult (J in Hz)	δ_H, mult (J in Hz)[a]
1	170.2, C	172.4, C		
2	105.6, C	106.5, C		
3	163.8, C	164.1, C		
4	101.7, CH	103.2, CH	6.21, d (2.5)	6.24, d (2.5)
5	162.3, C	164.1, C		
6	112.6, CH	113.9, CH	6.10, d (2.5)	6.11, d (2.5)
7	138.2, C	139.9, C		
8	46.3, CH_2	47.9, CH_2	α 4.38, d (18.2); β 3.87, d (18.2)	α 4.55, d (18.7); β 3.85, d (18.7)
9	204.4, C	206.7, C		
10	50.8, CH_2	52.9, CH_2	α 2.63, dd (15.8, 2.2) β 3.03, dd (15.8, 10.2)	α 2.68, dd (15.9, 1.3) β 3.09, dd (15.9, 10.4)
11	69.4, CH	71.6, CH	5.30, m	5.45, m
12	31.9, CH_2	33.5, CH_2	α 1.56, m; β 1.78, m	α 1.56, m; β 1.88, m
13	17.5, CH_2	19.2, CH_2	1.61, m	α 1.56, m; β 1.58, m
14	32.1, CH_2	34.0, CH_2	α 1.62, m; β 1.75, m	α 1.65, m; β 1.71, m
15	72.1, CH	74.1, CH	5.06, m	5.16, m
15-CH_3	18.4, CH_3	18.9, CH_3	1.21, d (6.3)	1.28, d (6.4)
1'	169.7, C	172.1, C		
2'	21.1, CH_3	21.3, CH_3	1.99, s	2.01, s

[a]The literature data [19] were recorded in CD_3OD-d_4; the solvent difference should be considered when comparing the chemical shifts.

Table S4. ^{1}H (400 MHz) and ^{13}C (100 MHz) NMR data for compound **4** in DMSO-d_6.

No.	**4**	
	δ_C, type	δ_H, mult (J in Hz)
1	170.2, C	
2	107.5, C	
3	161.9, C	
4	101.5, CH	6.21, d (2.4)
5	161.5, C	
6	112.2, CH	6.10, d (2.5)
7	137.2, C	
8	48.4, CH_2	α 4.37, d (18.8); β 3.84, d (18.7)
9	204.9, C	
10	49.0, CH_2	α 2.61, dd (13.8, 10.5) β 3.03, dd (13.9, 2.9)
11	68.7, CH	5.30, m
12	32.5, CH_2	α 1.52, m; β 1.70, m
13	19.7, CH_2	1.61, m
14	32.6, CH_2	α 1.52, m; β 1.69, m
15	73.5, CH	4.88, m
15-CH_3	20.4, CH_3	1.23, d (6.1)
1'	169.7, C	
2'	21.1, CH_3	2.01, s

Table S5. ^{1}H (400 MHz) and ^{13}C (125 MHz) NMR data for compound **5** in DMSO-d_6.

No.	**5**	
	δ_C, type	δ_H, mult (J in Hz)
1	171.1, C	
2	106.5, C	
3	165.8, C	
4	103.1, CH	6.37, d (2.4)
5	160.5, C	
6	112.8, CH	6.10, d (2.4)
7	138.3, C	
8	50.0, CH_2	α 4.95, m; β 3.58, m
9	204.8, C	
10	49.9, CH_2	α 2.60, m; β 2.92, dd (14.9, 9.8)
11	68.5, CH	5.47, m
12	33.2, CH_2	1.70, m
13	20.6, CH_2	α 1.64, m; β 1.39, m
14	32.8, CH_2	α 1.84, m; β 1.75, m
15	75.2, CH	4.97, m
15-CH_3	21.1, CH_3	1.33, d (6.3)
1'	173.9, C	
2'	34.7, CH_2	2.34, t (7.5)
3'	25.1, CH_2	1.63, m
4'	29.3, CH_2	1.33, m
5'	29.4, CH_2	1.31, m
6'	29.6, CH_2	1.27, m
7'	29.7, CH_2	1.25, m
8'	29.8, CH_2	1.25, m
9'	29.9, CH_2	1.25, m
10'	29.9, CH_2	1.25, m
11'	29.9, CH_2	1.25, m
12'	29.9, CH_2	1.25, m
13'	29.5, CH_2	1.29, m
14'	22.8, CH_2	1.25-1.29, m
15'	32.1, CH_2	1.25, m
16'	14.3, CH_3	0.88, m

Table S6. ^{1}H (400 MHz) and ^{13}C (125 MHz) NMR data for compound **6** in DMSO-d_6.

No.	**6**	
	δ_C, type	δ_H, mult (J in Hz)
1	169.0, C	
2	107.7, C	
3	159.6, C	
4	101.6, CH	6.17, d (2.1)
5	161.0, C	
6	105.2, CH	6.36, d (2.0)
7	139.9, C	
8	128.7, CH	6.60, d (15.6)
9	132.6, CH	5.99, dt (15.6, 6.9)
10	32.6, CH_2	2.12, m
11	25.1, CH_2	α 1.39, m;<br>β 1.47, m
12	38.5, CH_2	1.34, m
13	65.7, CH	3.57, m
14	23.7, CH_3	1.03, d (6.1)
1'	66.1, CH_2	α 4.12, dd (11.1, 6.0);<br>β 4.23, dd (11.1, 4.3)
2'	69.2, CH	3.73, m
3'	62.7, CH_2	3.40, m

Table S7. ^{1}H (400 MHz) and ^{13}C (125 MHz) NMR data for compound **7** in DMSO-d_6.

No.	**7**	
	δ_C, type	δ_H, mult (J in Hz)
1	165.7, C	
2	97.1, C	
3	162.7, C	
4	101.8, CH	6.21, s
5	166.8, C	
6	103.1, CH	6.26, s
7	139.5, C	
8	105.5, CH	6.40, s
9	155.1, C	
10	41.2, CH_2	α 2.54, m; β 2.41, m
11	67.6, CH	3.76, m
12	36.8, CH_2	1.39, m
13	25.2, CH_2	1.40, m
14	29.2, CH_2	1.23, m
15	25.4, CH_2	α 1.25, m; β 1.29, m
16	39.1, CH_2	1.28, m
17	65.8, CH	3.54, m
18	23.7, CH_3	1.01, d (6.1)

Table S8. ^1H (400 MHz) and ^{13}C (125 MHz) NMR data for compound **8** in DMSO-d_6.

No.	**8**			
	δ_C, type	δ_C, type^a	δ_H, mult (J in Hz)	δ_H, mult (J in Hz)^a
1	165.2, C	164.6, C		
2	97.4, C	99.7, C		
3	162.7, C	164.8, C		
4	102.0, CH	102.8, CH	6.27, d (2.2)	6.41, d (2.0)
5	166.8, C	166.7, C		
6	103.3, CH	103.8, CH	6.31, d (2.1)	6.45, d (2.0)
7	139.1, C	140.5, C		
8	106.9, CH	107.9, CH	6.51, s	6.51, s
9	151.1, C	152.5, C		
10	46.2, CH	47.2, CH	3.73, s	3.74, s
11	205.4, CH	205.1, CH		
12	41.7, CH_2	42.9, CH_2	2.54, m	2.62, t (7.2)
13	23.7, CH_2	24.3, CH_2	1.47, m	1.58, m
14	28.6, CH_2	30.1, CH_2	1.34-1.17, m	1.29, m
15	25.2, CH	26.4, CH	1.34-1.17, m	1.37, m
16	38.9, CH_2	40.1, CH_2	1.34-1.17, m	1.34, m
17	65.7, CH	67.5, CH	3.53, m	3.68, m
18	23.0, CH_3	24.1, CH_3	1.01, d (6.1)	1.09, d (6.2)

^{a}The literature data [26] were recorded in acetone-d_6; the solvent difference should be considered when comparing the chemical shifts.

Table S9. ^{1}H (400 MHz) and ^{13}C (100 MHz) NMR data for compound **9** in DMSO-d_6.

No.	**9**		
	δ_C, type	δ_H, mult (J in Hz)	δ_H, mult (J in Hz) [a]
1	164.8, C		
2	97.3, C		
3	162.8, C		
4	104.9, CH	6.24, d (2.1)	6.31, d (2.0)
5	167.5, C		
6	104.0, CH	6.32, d (2.1)	6.34, d (2.0)
7	139.3, C		
8	102.2, CH	6.51 (s)	6.37 (s)
9	151.0, C		
10	122.1, CH	6.16, dt (15.6, 1.4)	6.15, d (15.6)
11	135.7, CH	6.39, dt (15.9, 7.1)	6.54, dt (15.5, 6.9)
12	32.1, CH_2	2.20, m	2.27, m
13	24.7, CH_2	1.57-1.27, m	1.45-1.67, m
14	38.5, CH_2	1.57-1.27, m	1.17-1.34, m
15	65.6, CH	3.59, m	3.76, m
16	23.7, CH_3	1.04, d (6.1)	1.17, d (6.2)

[a]The literature data [26] were recorded in CD_3OD-d_4; the solvent difference should be considered when comparing the chemical shifts.

Table S10. ^{1}H (400 MHz) and ^{13}C (125 MHz) NMR data for compound **10** in DMSO-d_6.

No.	**10**			
	δ_C, type	δ_C, type[a]	δ_H, mult (J in Hz)	δ_H, mult (J in Hz)[a]
1	164.0, C	163.9		
2	100.3, C	99.4		
3	165.8, C	165.5 C		
4	104.9, CH	105.4, CH	6.27, s	6.20, s
5	165.8, C	167.3, C		
6	103.2, CH	104.2, CH	6.37, s	6.33, s
7	140.0, C	139.9, C		
8	102.5, CH	102.5, CH	6.53, s	6.56, s
9	152.2, C	151.6, C		
10	122.4, CH	122.8, CH	6.16, dt (15.6, 1.4)	6.14, d (15.5)
11	136.2, CH	135.6, CH	6.39, dt (15.9, 7.1)	6.33, m
12	32.1, CH_2	31.8, CH_2	2.24, m	2.27, m
13	22.8, CH_2	22.8, CH_2	1.77, m	1.61, m
14	42.9, CH_2	42.5, CH_2	2.47, m	2.42, m
15	208.8, CH	208.8, CH		
16	29.9, CH_3	29.8, CH_3	2.15, s	2.01, s

[a]The literature data [23] were recorded in DMSO-d_6.

Table S11. DP4+ probability analysis of compound **6**.

Functional		Solvent?		Basis Set		Type of Data	
mPW1PW91		PCM		6-311+G(d, p)		Shielding Tensors	
		DP4+	0.04%	0.33%	0.00%	99.63%	-
Nuclei	sp2?	Experimenta	Isomer 1	Isomer 2	Isomer 3	Isomer 4	Isomer 5
C	x	128.7	45.1	46.3	46.1	46.9	
C	x	132.6	45.4	45.2	40.7	44.1	
C	x	169	9.4	9.8	6.6	9.6	
C	x	107.7	80.7	80.5	79.5	80.6	
C	x	139.9	34.4	34.3	32.9	34.8	
C		66.1	122.2	122.2	122.7	121.3	
C	x	159.6	14.8	14.9	15.8	14.8	
C	x	101.6	82.6	82.7	82.4	82.5	
C	x	161	18.8	18.8	19.1	19.0	
C	x	105.2	74.4	74.7	73.8	74.8	
C		32.6	150.1	151.9	153.5	152.1	
C		65.7	114.50	115.61	114.40	118.89	
C		38.5	146.58	147.33	149.02	147.85	
C		25.1	158.01	160.59	154.77	159.34	
C		69.2	113.41	112.97	106.23	113.15	
C		62.7	124.07	123.87	119.36	123.02	
C		23.7	163.12	165.25	161.32	165.42	

Functional	Solvent?		Basis Set		Type of Data	
mPW1PW91	PCM		6-311+G(d, p)		Shielding Tensors	
	Isomer 1	Isomer 2	Isomer 3	Isomer 4	Isomer 5	Isomer 6
sDP4+ (H data)	-	-	-	-	-	-
sDP4+ (C data)	0.46%	6.32%	0.00%	93.22%	-	-
sDP4+ (all data)	0.46%	6.32%	0.00%	93.22%	-	-
uDP4+ (H data)	-	-	-	-	-	-
uDP4+ (C data)	6.42%	4.39%	0.00%	89.20%	-	-
uDP4+ (all data)	6.42%	4.39%	0.00%	89.20%	-	-
DP4+ (H data)	-	-	-	-	-	-
DP4+ (C data)	0.04%	0.33%	0.00%	99.63%	-	-
DP4+ (all data)	0.04%	0.33%	0.00%	99.63%	-	-

Note: Enantiomeric pairs cannot be distinguished by conventional NMR chemical shifts in an achiral medium. Therefore, the DP4+ results were interpreted only as tentative support for the relative stereochemical relationship of compound **6** and were not used to establish its absolute configuration.

Table S12. DP4+ probability analysis of compound **7**.

Functional		Solvent?		Basis Set		Type of Data	
mPW1PW91		PCM		6-31+G(d, p)		Shielding Tensors	
		DP4+	0.00%	97.06%	0.00%	2.94%	-
Nuclei	sp2?	Experimental	Isomer 1	Isomer 2	Isomer 3	Isomer 4	Isomer 5
C	x	101.8	97.8	97.5	97.1	97.8	
C	x	166.8	35.3	35.5	34.5	35.4	
C	x	103.1	96.3	96.4	95.3	96.2	
C	x	139.5	57.3	57.6	58.2	57.5	
C	x	97.1	97.6	97.3	98.0	98.0	
C	x	162.7	34.6	34.8	34.7	34.5	
C	x	105.5	89.6	89.4	88.7	91.6	
C	x	155.1	38.3	39.7	39.6	36.9	
C	x	165.7	32.0	32.4	32.7	33.1	
C		41.2	155.8	152.6	156.4	154.5	
C		67.6	123.2	124.2	123.5	122.8	
C		36.8	159.27	158.04	162.16	157.94	
C		25.2	168.54	167.00	173.14	168.35	
C		29.2	168.27	163.55	166.22	164.47	
C		25.4	169.70	166.84	168.43	166.35	
C		39.1	158.23	154.90	157.04	155.14	
C		65.8	129.95	125.81	125.41	126.99	
C		23.7	171.99	170.98	172.50	170.89	

Functional	Solvent?		Basis Set		Type of Data	
mPW1PW91	PCM		6-31+G(d, p)		Shielding Tensors	
	Isomer 1	Isomer 2	Isomer 3	Isomer 4	Isomer 5	Isomer 6
sDP4+ (H data)	-	-	-	-	-	-
sDP4+ (C data)	18.31%	74.24%	0.39%	7.06%	-	-
sDP4+ (all data)	18.31%	74.24%	0.39%	7.06%	-	-
uDP4+ (H data)	-	-	-	-	-	-
uDP4+ (C data)	0.01%	75.83%	0.00%	24.16%	-	-
uDP4+ (all data)	0.01%	75.83%	0.00%	24.16%	-	-
DP4+ (H data)	-	-	-	-	-	-
DP4+ (C data)	0.00%	97.06%	0.00%	2.94%	-	-
DP4+ (all data)	0.00%	97.06%	0.00%	2.94%	-	-

Note: Enantiomeric pairs cannot be distinguished by conventional NMR chemical shifts in an achiral medium. Therefore, the DP4+ results were interpreted only as tentative support for the relative stereochemical relationship of compound **7** and were not used to establish its absolute configuration.

Table S13. DFT-optimized structures for low-energy conformers of **5a**.

Conformers	Conf. A	Conf. B	Conf. C
DFT-optimized structures	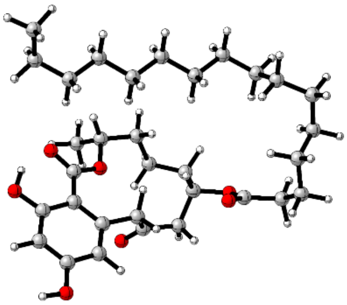	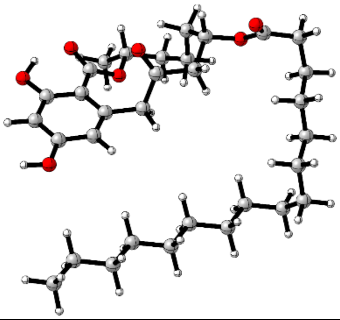	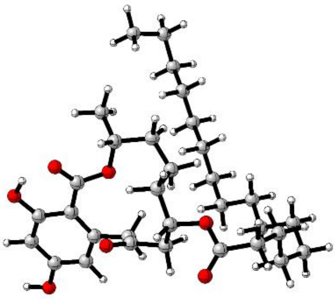
Population	84.96%	11.57%	3.47%

Table S14. DFT-optimized structures for low-energy conformers of **6a**.

**Conformers**	**Conf. A**	**Conf. B**	**Conf. C**
DFT-optimized structures	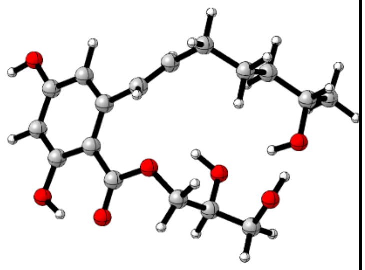	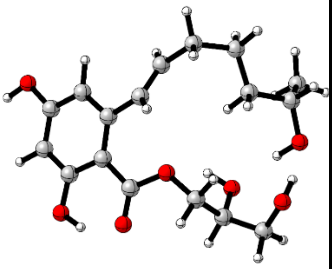	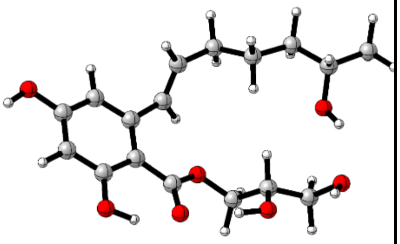
Population	28.83%	71.12%	0.05%

Table S15. DFT-optimized structures for low-energy conformers of **6b**.

**Conformers**	**Conf. A**	**Conf. B**	**Conf. C**
DFT-optimized structures	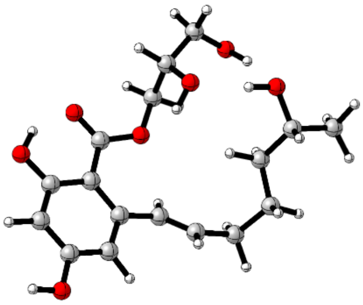	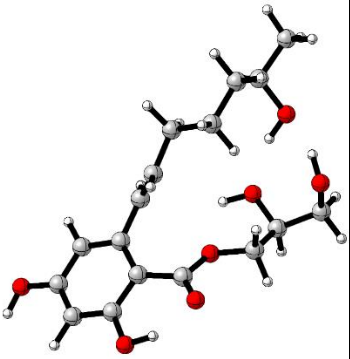	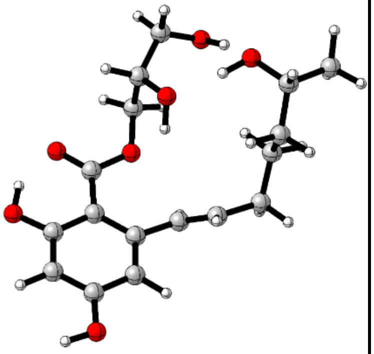
Population	64.28%	1.43%	2.42%
**Conformers**	**Conf. D**	**Conf. E**	
DFT-optimized structures	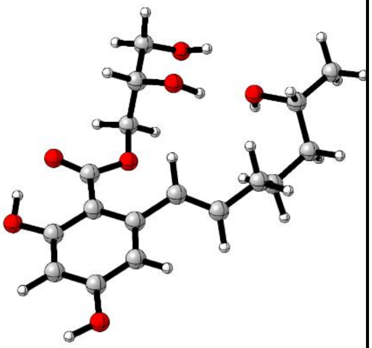	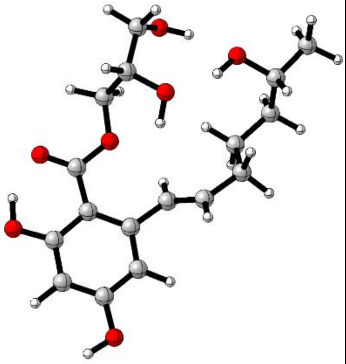	
Population	5.96%	25.91%	

Table S16. DFT-optimized structures for low-energy conformers of **6c**.

Conformers	Conf. A	Conf. B	
DFT-optimized structures	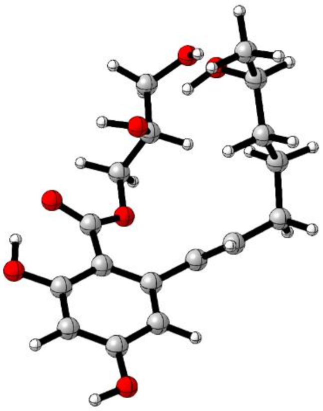	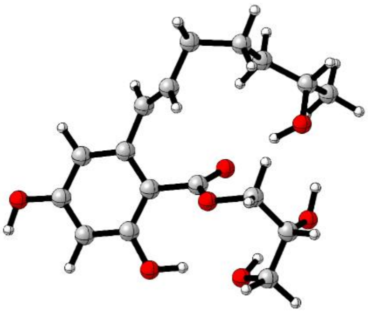	
Population	99.92%	0.08%	

Table S17. DFT-optimized structures for low-energy conformers of **6d**.

**Conformers**	**Conf. A**	**Conf. B**	**Conf. C**
DFT-optimized structures	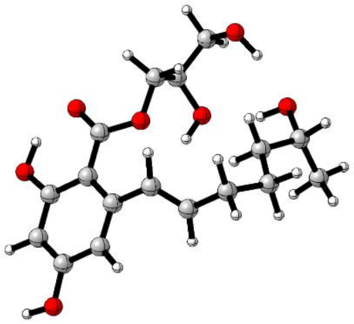	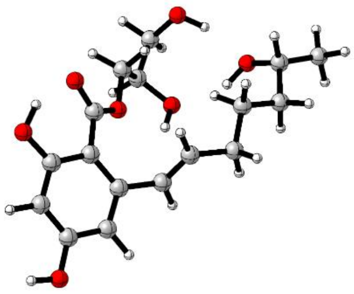	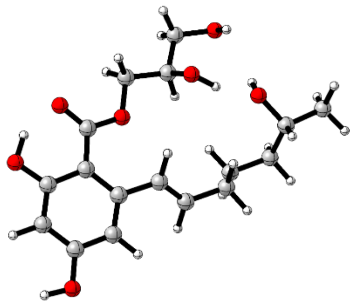
Population	17.16%	0.25%	18.21%
**Conformers**	**Conf. D**	**Conf. E**	
DFT-optimized structures	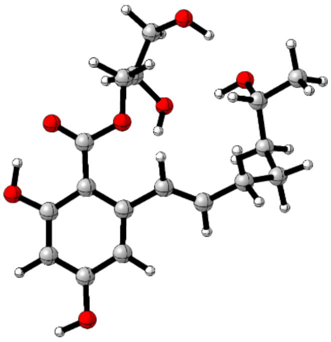	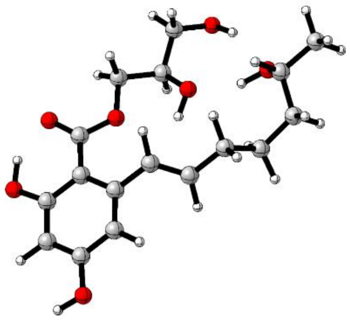	
Population	47.43%	16.96%	

Table S18. DFT-optimized structures for low-energy conformers of **7a**.

**Conformers**	**Conf. A**	**Conf. B**	**Conf. C**
DFT-optimized structures	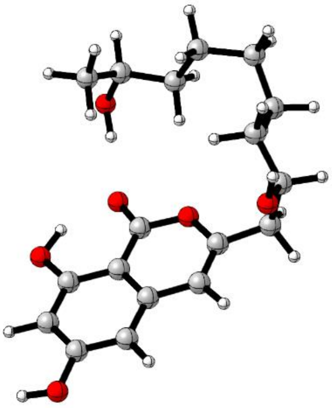	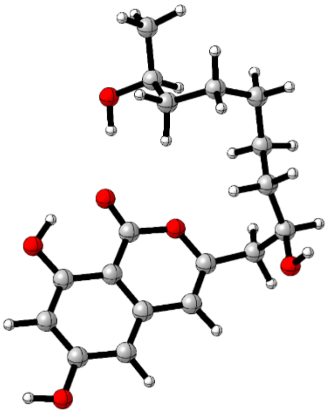	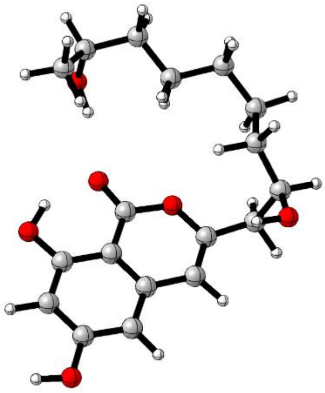
Population	8.76%	13.84%	5.80%
**Conformers**	**Conf. D**	**Conf. E**	**Conf. F**
DFT-optimized structures	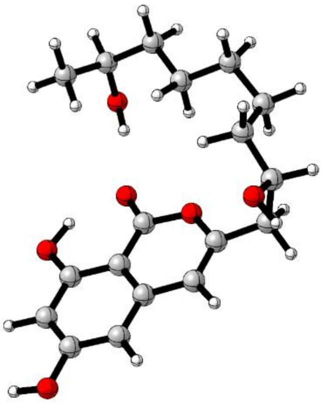	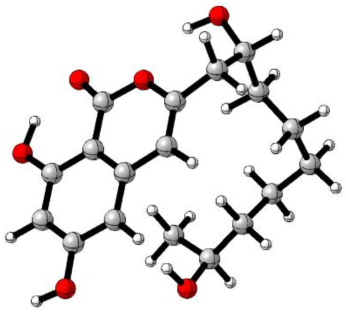	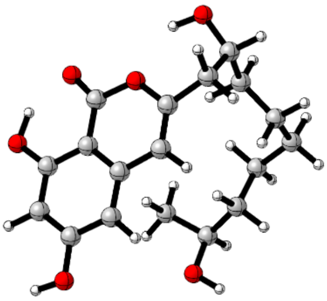
Population	4.26%	6.18%	6.22%
**Conformers**	**Conf. G**		
DFT-optimized structures	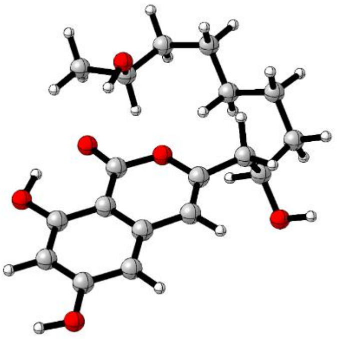		
Population	54.96%		

Table S19. DFT-optimized structures for low-energy conformers of **7b**.

**Conformers**	**Conf. A**	**Conf. B**	**Conf. C**
DFT-optimized structures	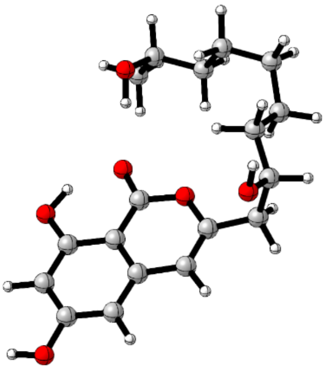	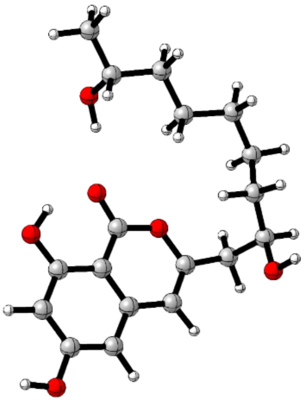	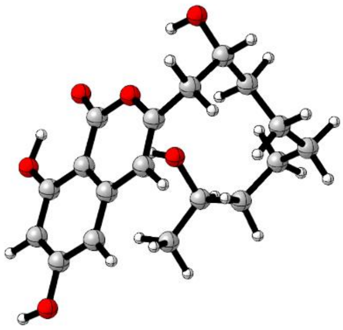
Population	10.48%	5.68%	0.02%
**Conformers**	**Conf. D**	**Conf. E**	**Conf. F**
DFT-optimized structures	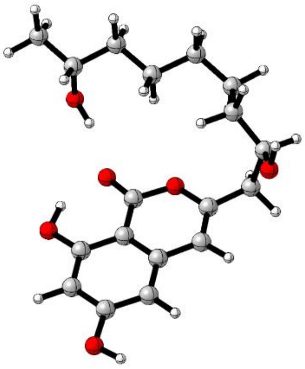	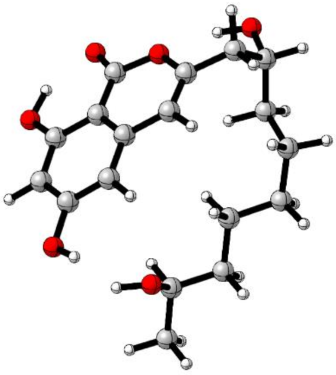	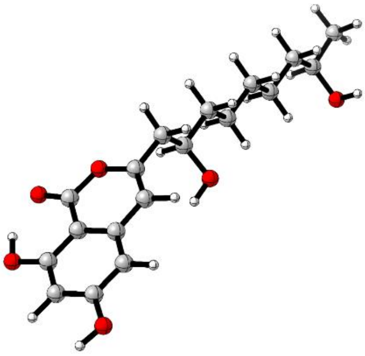
Population	5.93%	0.02%	66.45%
**Conformers**	**Conf. G**		
DFT-optimized structures	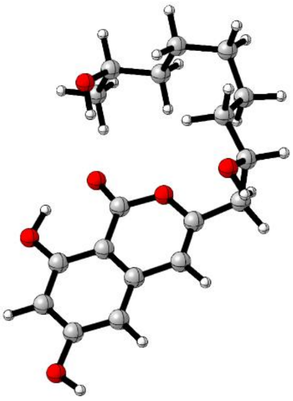		
Population	11.41%		

Table S20. DFT-optimized structures for low-energy conformers of **7c**.

**Conformers**	**Conf. A**	**Conf. B**	**Conf. C**
DFT-optimized structures	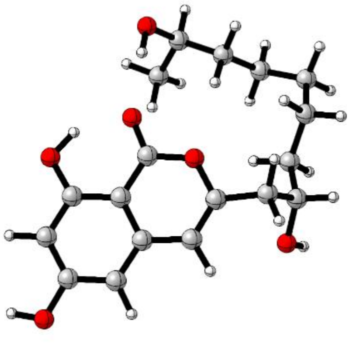	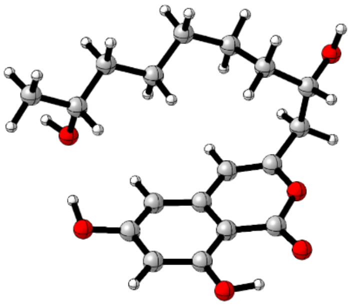	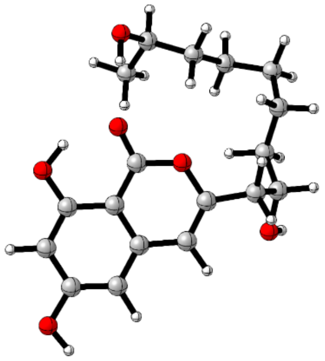
Population	8.89%	30.67%	9.45%
**Conformers**	**Conf. D**	**Conf. E**	**Conf. F**
DFT-optimized structures	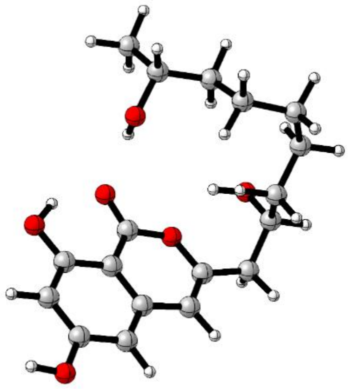	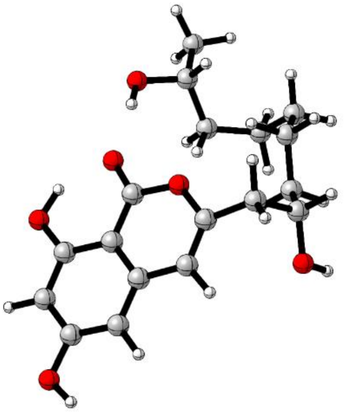	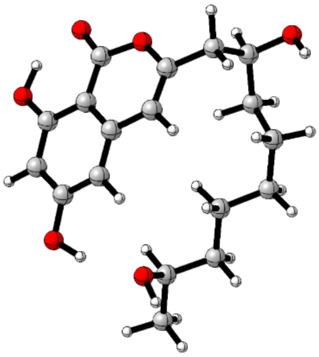
Population	8.77%	15.06%	24.65%
**Conformers**	**Conf. G**		
DFT-optimized structures	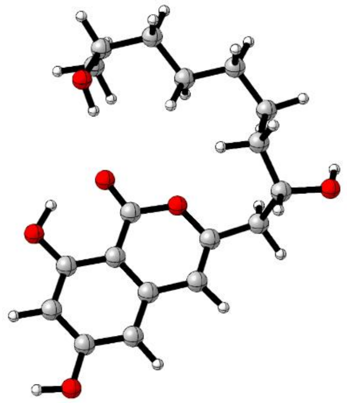		
Population	2.51%		

Table S21. DFT-optimized structures for low-energy conformers of **7d**.

**Conformers**	**Conf. A**	**Conf. B**	**Conf. C**
DFT-optimized structures	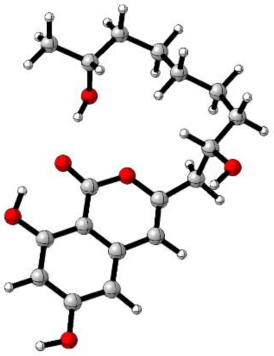	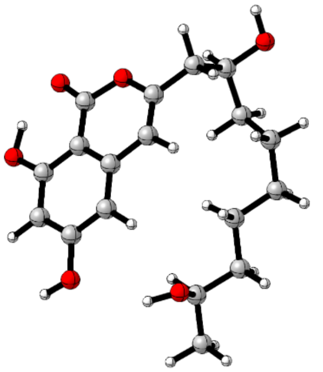	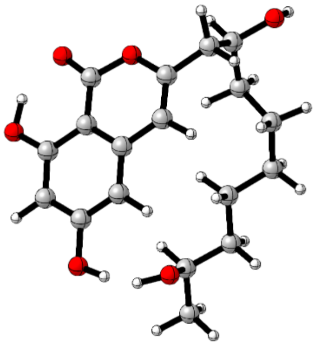
Population	14.60%	0.08%	0.02%
**Conformers**	**Conf. D**	**Conf. E**	**Conf. F**
DFT-optimized structures	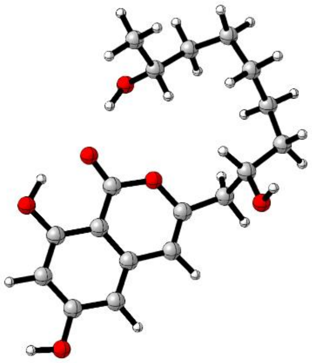	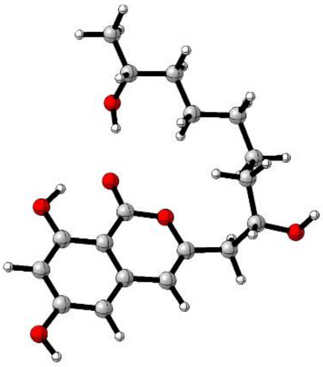	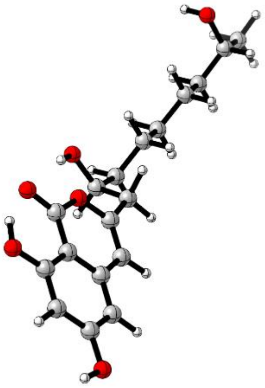
Population	6.00%	6.39%	20.17%
**Conformers**	**Conf. G**	**Conf. H**	
DFT-optimized structures	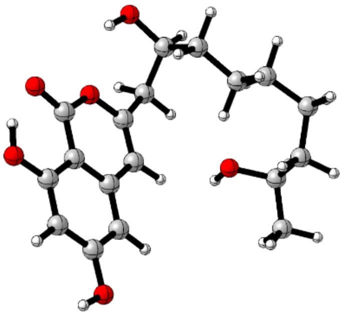	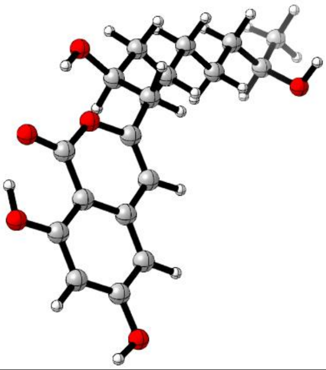	
Population	0.08%	52.67%	

Table S22. Predicted conserved domains of genes in the putative *res* biosynthetic gene cluster.

Gene	Domain	Pfam ID	i-Evalue	Env_Start	Env_End	Confidence
resA	Fungal_trans	PF04082.24	3.20E-14	19	292	Medium
resB	FAD_binding_3	PF01494.26	1.00E-21	8	386	Medium
resC	SAT	PF16073.11	5.40E-10	55	257	Medium
resC	ketoacyl-synt	PF00109.33	2.80E-72	383	629	High
resC	Ketoacyl-synt_C	PF02801.29	1.70E-31	637	756	Medium
resC	Acyl_transf_1	PF00698.27	1.60E-25	915	1223	Medium
resC	PP-binding	PF00550.32	2.90E-09	1670	1736	Medium
resC	HTH_51	PF18558.9	7.90E-29	1805	1892	Medium
resC	Methyltransf_12	PF08242.19	4.40E-13	1997	2098	Medium
resC	NAD_binding_4	PF07993.19	1.70E-24	2236	2492	Medium
resC	Epimerase	PF01370.28	3.10E-06	2234	2462	Medium
resD	ketoacyl-synt	PF00109.33	4.80E-57	45	230	High
resD	Ketoacyl-synt_C	PF02801.29	1.30E-38	238	353	Medium
resD	KAsynt_C_assoc	PF16197.12	6.50E-12	355	488	Medium
resD	Acyl_transf_1	PF00698.27	8.70E-42	538	845	Medium
resD	PKS_DH_N	PF21089.4	2.40E-14	931	1047	Medium
resD	PS-DH	PF14765.13	1.50E-19	1073	1239	Medium
resD	Methyltransf_12	PF08242.19	1.30E-09	1426	1533	Medium
resD	NAD-bd_HRPKS_sdrA	PF23114.1	1.90E-07	1658	1803	Medium
resD	ADH_zinc_N	PF00107.33	3.60E-13	1975	2100	Medium
resD	ADH_zinc_N_2	PF13602.13	9.60E-14	2009	2149	Medium
resD	KR	PF08659.17	9.30E-59	2172	2355	High
resD	adh_short	PF00106.32	3.50E-11	2173	2335	Medium
resD	ACP_SdgA_C	PF23297.2	0.00086	2458	2544	Low
resE	Fungal_trans	PF04082.24	4.00E-17	120	305	Medium
resF	MFS_1	PF07690.22	5.20E-34	58	435	Medium
resG	FAD_binding_4	PF01565.29	6.70E-25	45	181	Medium
